# *Deinococcus geothermalis*: The Pool of Extreme Radiation Resistance Genes Shrinks

**DOI:** 10.1371/journal.pone.0000955

**Published:** 2007-09-26

**Authors:** Kira S. Makarova, Marina V. Omelchenko, Elena K. Gaidamakova, Vera Y. Matrosova, Alexander Vasilenko, Min Zhai, Alla Lapidus, Alex Copeland, Edwin Kim, Miriam Land, Konstantinos Mavromatis, Samuel Pitluck, Paul M. Richardson, Chris Detter, Thomas Brettin, Elizabeth Saunders, Barry Lai, Bruce Ravel, Kenneth M. Kemner, Yuri I. Wolf, Alexander Sorokin, Anna V. Gerasimova, Mikhail S. Gelfand, James K. Fredrickson, Eugene V. Koonin, Michael J. Daly

**Affiliations:** 1 National Center for Biotechnology Information, National Library of Medicine, National Institutes of Health, Bethesda, Maryland, United States of America; 2 Department of Pathology, Uniformed Services University of the Health Sciences (USUHS), Bethesda, Maryland, United States of America; 3 US Department of Energy, Joint Genome Institute, Walnut Creek, California, United States of America; 4 US Department of Energy, Joint Genome Institute, Los Alamos National Laboratory, Los Alamos, New Mexico, United States of America; 5 Environmental Research Division and Advanced Photon Source, Argonne National Laboratory, Argonne, Illinois, United States of America; 6 Research Institute of Genetics and Selection of Industrial Microorganisms, Moscow, Russia; 7 Institute for Information Transmission Problems of RAS, Moscow, Russia; 8 Faculty of Bioengineering and Bioinformatics, M. V. Lomonosov Moscow State University, Moscow, Russia; 9 Biological Sciences Division, Pacific Northwest National Laboratory, Richland, Washington, United States of America; National Cancer Institute, United States of America

## Abstract

Bacteria of the genus *Deinococcus* are extremely resistant to ionizing radiation (IR), ultraviolet light (UV) and desiccation. The mesophile *Deinococcus radiodurans* was the first member of this group whose genome was completely sequenced. Analysis of the genome sequence of *D. radiodurans*, however, failed to identify unique DNA repair systems. To further delineate the genes underlying the resistance phenotypes, we report the whole-genome sequence of a second *Deinococcus* species, the thermophile *Deinococcus geothermalis*, which at its optimal growth temperature is as resistant to IR, UV and desiccation as *D. radiodurans*, and a comparative analysis of the two *Deinococcus* genomes. Many *D. radiodurans* genes previously implicated in resistance, but for which no sensitive phenotype was observed upon disruption, are absent in *D. geothermalis*. In contrast, most *D. radiodurans* genes whose mutants displayed a radiation-sensitive phenotype in *D. radiodurans* are conserved in *D. geothermalis*. Supporting the existence of a *Deinococcus* radiation response regulon, a common palindromic DNA motif was identified in a conserved set of genes associated with resistance, and a dedicated transcriptional regulator was predicted. We present the case that these two species evolved essentially the same diverse set of gene families, and that the extreme stress-resistance phenotypes of the *Deinococcus* lineage emerged progressively by amassing cell-cleaning systems from different sources, but not by acquisition of novel DNA repair systems. Our reconstruction of the genomic evolution of the *Deinococcus-Thermus* phylum indicates that the corresponding set of enzymes proliferated mainly in the common ancestor of *Deinococcus*. Results of the comparative analysis weaken the arguments for a role of higher-order chromosome alignment structures in resistance; more clearly define and substantially revise downward the number of uncharacterized genes that might participate in DNA repair and contribute to resistance; and strengthen the case for a role in survival of systems involved in manganese and iron homeostasis.

## Introduction

*Deinococcus geothermalis* belongs to the *Deinococcus*-*Thermus* group, which is deeply branched in bacterial phylogenetic trees and has putative relationships with cyanobacteria [1], [2]. The extremely radiation-resistant family *Deinococcaceae* is comprised of greater than twenty distinct species [3] that can survive acute exposures to ionizing radiation (IR) (10 kGy), ultraviolet light (UV) (1 kJ/m^2^), and desiccation (years) [4], [5]; and can grow under chronic IR (60 Gy/hour) [6]. *D. geothermalis* was originally isolated from a hot pool at the Termi di Agnano, Naples, Italy [7], and subsequently identified at other locations poor in organic nutrients including industrial paper machine water [8], deep ocean subsurface environments [9], and subterranean hot springs in Iceland [10].

*D. geothermalis* is distinct from most members of the genus *Deinococcus* in that it is a moderate thermophile, with an optimal growth temperature (T_opt_) of 50°C [7], is not dependent on an exogenous source of amino acids or nicotinamide for growth [11], [12], is capable of forming biofilms [8], and possesses membranes with very low levels of unsaturated fatty acids compared to the other species [7]. Based on the ability of wild-type and engineered *D. geothermalis* and *D. radiodurans* to reduce a variety of metals including U(VI), Cr(VI), Hg(II), Tc(VII), Fe(III) and Mn(III,IV) [11], [13], these two species have been proposed for bioremediation of radioactive waste sites maintained by the US Department of Energy (DOE) [11], [14], [15]. These characteristics were the impetus for whole-genome sequencing of *D. geothermalis* at DOE's Joint Genome Institute, and comparison with the mesophilic *D. radiodurans* (T_opt_, 32°C), to date the only other extremely IR-resistant bacterium for which a whole-genome sequence has been acquired [16].

Chromosomal and plasmid DNAs in extremely resistant bacteria are as susceptible to IR-induced DNA double strand breaks (DSBs) as in sensitive bacteria [5], [17]–[19] and broad-based experimental and bioinformatic studies have converged on the conclusion that *D. radiodurans* uses a conventional set of DNA repair and protection functions, but with a far greater efficiency than IR-sensitive bacteria [17], [20], [21]. This apparent contradiction is exemplified by work which showed that the repair protein DNA polymerase I (PolA) of *D. radiodurans* supports exceptionally efficient DNA replication at the earliest stages of recovery from IR, and could account for the high fidelity of RecA-mediated DNA fragment assembly [22]. Paradoxically, however, IR-, UV-, and mitomycin-C (MMC)-sensitive *D. radiodurans pol*A mutants are fully complemented by expression of the *pol*A gene from the IR-sensitive *Escherichia coli* [4].

The reason why repair proteins, either native or cloned, in *D. radiodurans* function so much better after irradiation than in sensitive bacteria is unknown. The prevailing hypotheses of extreme IR resistance in *D. radiodurans* fall into three categories: (i) chromosome alignment, morphology and/or repeated sequences facilitate genome reassembly [5], [21], [23], [24]; (ii) a subset of uncharacterized genes encode functions that enhance the efficiency of DNA repair [20]; and (iii) non-enzymic Mn(II) complexes present in resistant bacteria protect proteins, but not DNA, from oxidation during irradiation, with the result that conventional enzyme systems involved in recovery survive and function with far greater efficiency than in sensitive bacteria [17], [23]. The extraordinary survival of *Deinococcus* bacteria following irradiation has also given rise to some rather whimsical descriptions of their derivation, including that they evolved on Mars [25]. On the basis of whole-genome comparisons between two *Deinococcus* genomes and two *Thermus* genomes, we present a reconstruction of evolutionary events that are inferred to have occurred both before and after the divergence of the *D. radiodurans* and *D. geothermalis* lineages. We revise down substantially the number of potential genetic determinants of radiation resistance, predict a *Deinococcus* radiation response regulon, and consider the implications of these comparative-genomic findings for different models of recovery.

## Results and Discussion

### Resistance to IR and UV

**Figure 1:**
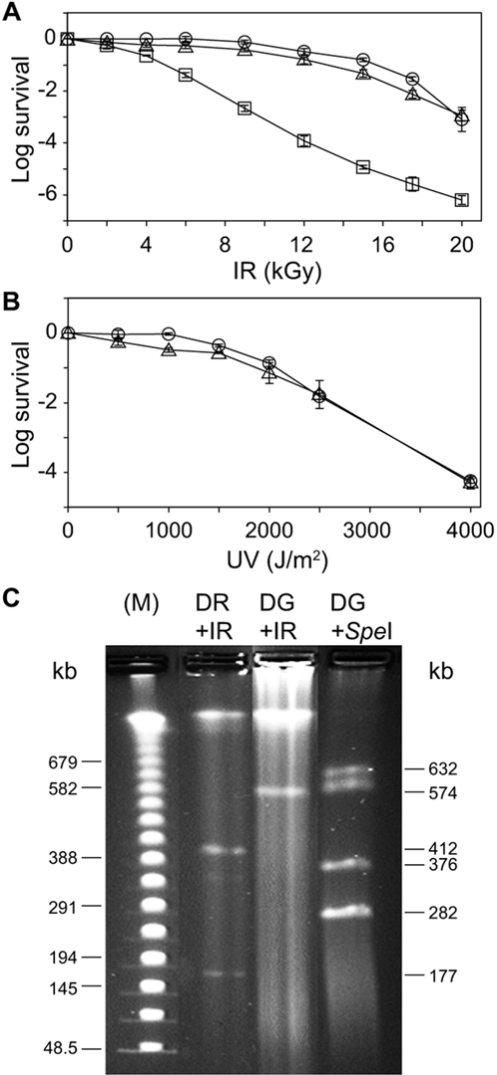
Radiation resistance and genome structure of *D. geothermalis* and *D. radiodurans*. A, IR (^60^Co, 5.5 kGy/h). B, UV (254 nm) (3 J/m^2^ s^−1^). Open circle, *D. radiodurans* (32°C); open triangle, *D. geothermalis* (50°C); and open square, *D. geothermalis* (32°C). Values are from three independent trials with standard deviations shown. At near-optimal growth temperatures, the 10% survival values (D_10_) following IR for *D. radiodurans* (32°C) and *D. geothermalis* (50°C) are 15 kGy; for *E. coli*, 0.7 kGy (37°C) [5]; and for *T. thermophilus* (HB27) 0.8 kGy (65°C) [27]. C, PFGE of genomic DNA prepared from irradiated (0.2 kGy) *D. radiodurans* (DR+IR) and *D. geothermalis* (DG+IR); and genomic DNA from non-irradiated *D. geothermalis* digested with *Spe*I (DG+*Spe*I). (M) PFGE DNA size markers. PFGE was as described previously [77].

One approach to delineating a minimal set of genes involved in extreme resistance is to compare the whole-genome sequences of two phylogenetically related but distinct species that are equally resistant, whereby genes that are unique to both organisms are ruled out, whereas shared genes are pooled as candidates for involvement in resistance. We show that D. geothermalis (DSM 11300) and D. radiodurans (ATCC BAA-816) are equally resistant to IR (^60^Co) (Figure 1A) and UV (254 nm) (Figure 1B) when pre-grown and recovered at their optimal growth temperatures, 50°C and 32°C, respectively. When recovered at 50°C, the survival of D. geothermalis exposed to 12 kGy was 1,000 times greater than at 32°C (Figure 1A) [7]. The extreme resistance to desiccation of D. geothermalis recovered at 50°C was demonstrated previously [5]. Thus, D. geothermalis and D. radiodurans are well-suited to defining a conserved set of genes responsible for extreme resistance.

### Genome Sequence and Structure: General Features

The random shotgun method [16] was used to acquire the complete sequence of the *D. geothermalis* (DSM 11300) genome, that is comprised of a main chromosome (2,467,205 base pairs (bp)), and two megaplasmids (574,127 bp and 205,686 bp). The general structure of the predicted *D. geothermalis* genome was tested by pulsed field gel electrophoresis (PFGE) of genomic DNA linearized *in vivo* by exposure to IR (0.2 kGy), and by restriction endonuclease (*Spe*I) cleavage (Figure 1C). The IR-treatment revealed the existence of a ∼570 kb megaplasmid in *D. geothermalis*, and the *Spe*I-treatment yielded the expected number of chromosomal bands: 3 singlets (632 kb, 376 kb and 282 kb) and one doublet (574/579 kb); the plasmids do not contain a *Spe*I site. In comparison, IR-treated *D. radiodurans* (ATCC BAA-816) subjected to PFGE displayed the presence of the DR412 (412 kb) and DR177 (177 kb) megaplasmids, previously observed [26]. The approximately 206 kb *D. geothermalis* megaplasmid was not visualized by PFGE although its size lies between the two *D. radiodurans* megaplasmids, which were readily observed (Figure 1C). Consistently, the abundance of DNA clones for the 206 kb megaplasmid was significantly lower than the 574 kb megaplasmid during construction of the *D. geothermalis* genome-library used for sequencing (data not shown). Thus, the 574 kb megaplasmid of *D. geothermalis* exists at higher copy-number than the 206 kb megaplasmid.

### Genome Comparison: General Features

Comparison of the general genome features of *D. geothermalis* and *D. radiodurans* revealed major differences in genome partitioning, and in the number of noncoding repeats (SNRs) (Table 1).

**Table 1:** General Characteristics

	*D. geothermalis* 3.27 Mbp	*D. radiodurans* 3.28 Mbp
**Main Chromosome**	2.46 Mbp (2,335 ORFs)	2.65 Mbp (2,629 ORFs)
**Megaplasmid-1^st^**	0.574 Mbp (522 ORFs)	0.412 Mbp (368 ORFs)
**Megaplasmid-2^nd^**	0.206 Mbp (205 ORFs)	0.177 Mbp (145 ORFs)
**Plasmid**	Not present	0.046 Mbp (39 ORFs)
**Prophages**	1 region (∼70 ORFs)	2 regions (∼75 ORFs)
**IS elements**	∼84 (∼80 kb)	52 (∼62 kb)
**CRISPRs**	6 regions (2 types)	Not present
**SNRs**	Not present	295 (at least 9 types)

#### Genome Partitioning

We previously demonstrated homologous relationships between the DR412 megaplasmid of *D. radiodurans* and the sole 233 kb megaplasmid (pTT27) of *T. thermophilus* [27]. Based on the gene contents of DR412 and pTT27, we concluded that these megaplasmids evolved from a common ancestor (Figure S1), are essential to the survival of both species, and appear to serve as a sink for horizontally transferred genes [27]. In contrast, the 574 kb megaplasmid (DG574) of *D. geothermalis* is distinct from pTT27, and appears to have been derived from a fusion of DR412 and DR177 (Table S1), followed by numerous rearrangements. Levels of gene order conservation for the *D. geothermalis* and *D. radiodurans* chromosomes and megaplasmids were determined by genomic dot plots [28] (Figure S2). The dot plots of the chromosomes showed a clear pattern characteristic of chromosomes of relatively closely related bacteria that retain significant colinearity of the gene order. The X-shape pattern is thought to arise from inversions of a chromosomal segment around the origin of replication [28]. By contrast, DR412 and DR177 did not display any discernable colinearity (Figure S2B), indicating substantially greater levels of rearrangement in the megaplasmids.

#### Repeats and Prophages

Dozens of small noncoding repeats (SNRs) of an unusual, mosaic structure have been identified in the *D. radiodurans* genome, suggesting a possible role in resistance [29]. In stark contrast, no mosaic-type SNRs were found in the *D. geothermalis* genome (Table 1), suggesting that SNRs are not involved in recovery from radiation or desiccation [26], [29], [30]. Further, there are about 20 DNA repeats in *D. radiodurans* that contain oligoG stretches (Figure S3). Such DNA sequences might adopt an ordered helical structure (G-quadruplex), predicted to form parallel four-stranded complexes capable of promoting chromosomal alignment [31]. However, the absence of such oligoG stretches in the G-rich sequence of *D. geothermalis* (G+C content, 66%) indicates that G-quartets are not essential for resistance. In contrast, the *D. geothermalis* genome contains CRISPR repeats [32], whereas *D. radiodurans* does not (Table 1). CRISPR repeats are part of a predicted RNA-interference-based system implicated in immunity to phages and integrative plasmids [33], [34]. Since no homologous prophages were identified in the two *deinococci*, and no CRISPR repeats are present in *D. radiodurans*, these sequences apparently have no role in determining levels of resistance either.

The 206 kb *D. geothermalis* megaplasmid (DG206), predicted by genome sequencing, is in lower copy-number than DG574 (Figure 1C). The presence of DG206 in genomic DNA preparations was confirmed in *D. geothermalis* (DSM 11300) DNA samples used for sequencing and from independent preparations by polymerase chain reaction (PCR) using DG206-specific primers that yielded DNA products of the predicted sizes (Figure S4). DG206 contains 205 predicted open reading frames (ORFs), of which 103 have significant similarity to genes in current databases; approximately 40 are identical to genes in either the *D. geothermalis* chromosome or DG574; and 28 have homologs in *D. radiodurans*, including 3 ORFs encoding highly diverged single-stranded DNA-binding proteins. Among other sequences of interest in DG206 are 22 transposon-related ORFs; 11 ORFs related to phage proteins; and 5 ORFs related to conjugal plasmid replication systems. In summary, DG206 is enriched for phage-, integrative plasmid- or transposon-related ORFs, but encodes no known metabolic enzymes and very few replication or repair proteins. Thus, DR206 seems to mimic the trend seen for ORFs in the smallest plasmid (46 kb) of *D. radiodurans* [16], [21], with no predicted genes implicated in resistance.

### The *Deinococcus*-*Thermus* Group: Gene-Gain and Gene-Loss

**Figure 2:**
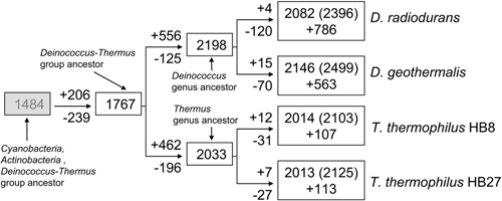
Whole genome evolutionary reconstructions for *D. radiodurans, D. geothermalis, T. thermophilus* (HB8) and *T. thermophilus* (HB27). For each internal node of tree (open boxes), the inferred number of tdCOGs is shown. For each tree branch the inferred number of tdCOGs lost (minus sign) and gained (plus sign) is shown. For the deep ancestor of the *Cyanobacteria, Actinobacteria* and *Deinococcus-Thermus* group (shaded box), the inferred number of COGs is shown. For the extant species, the number of tdCOGs, the number of proteins in tdCOGs (in parentheses), and the number of “free” (not assigned to tdCOGs) proteins (plus sign) are shown.

Our previous analysis of the major events in the evolution of the *Deinococcus*-*Thermus* group was based on *D. radiodurans* (ATCC BAA-816) and *T. thermophilus* strain HB27 [27]. The current study includes additional comparisons with *D. geothermalis* (DSM 11300) and a second strain of *T. thermophilus* (HB8). Based on the standard approach of COGs (clusters of orthologous groups of proteins) [35], [36], COGs for *Deinococcus* and *Thermus* (tdCOGs) were constructed (Table S2). The tdCOGs were used as a framework for the whole-genome comparisons and evolutionary reconstructions (Figure 2). Using a weighted parsimony method and distantly related bacteria as outgroups, the evolutionary reconstructions revealed significant and independent expansion of the repertoire of genes in the *Deinococcus* and *Thermus* lineages following their divergence from a common ancestor. The expansion appears to have occurred through both lineage-specific duplications and gene acquisition via horizontal gene transfer (HGT). The high level of protein family expansion (paralogy), and the larger complement of species-specific genes acquired principally by HGT, could account for the existence of 600–900 more genes in *Deinococcus* than *Thermus*.

### The Common Ancestor of the *Deinococcus* Lineage: Trends of Gene-Gain and Gene-Loss

Our previous comparative analysis of *T. thermophilus* and *D. radiodurans* identified several evolutionary trends that correlate with the distinct phenotypes of these bacterial lineages [27]. These trends were further refined through the analysis of the *D. geothermalis* sequence, and the unique features of the *Deinococcus* lineage were used to better define the pathways implicated in extreme radiation resistance (Table S2). One such trend in *Deinococcus*, in comparison to the inferred common ancestor of the *Deinococcus-Thermus* group, is the acquisition of a set of genes involved in transcriptional regulation and signal transduction. Examples of acquired transcriptional regulators include two proteins of the AsnC family, two proteins of the GntR family, and one protein of the IclR family. These families likely are involved in amino acid degradation and metabolism [37]–[39]. Further, the *Deinococcus* lineage acquired at least six TetR and MerR family regulators dedicated to diverse stress response pathways [40], [41]. Among the acquired signal transduction genes, the most notable examples are two-component regulators of the NarL family (four distinct tdCOGs) involved in the regulation of a variety of oxygen and nitrate-dependent pathways of *Escherichia coli* [42]; and the presence of several diguanylate cyclase (GGDEF) domain-containing proteins supports an increased role of cyclic diGMP in *Deinococci*. A second evolutionary trend in *Deinococcus* is the acquisition of genes encoding proteins involved in nucleotide metabolism, in particular, degradation and salvage [43]–[45]. For example, this group includes genes for xanthine dehydrogenase, urate oxidase, deoxynucleoside kinases, thymidine kinase, FlaR-like kinase, and two UshA family 5′-nucleotidases.

Other gene-gains in *Deinococcus* relative to *Thermus* include genes for enzymes of amino acid catabolism and the tricarboxylic acid (TCA) cycle (Table S2). Beyond the differences reported previously [11], [12], the new reconstructions indicate that several catabolic genes of *Deinococcus* were already present in the *Deinococcus-Thermus* common ancestor. Following their divergence, however, the *Thermus* lineage appears to have lost many of these systems, including all enzymes involved in histidine degradation. By contrast, the *Deinococcus* lineage not only retained a majority of the predicted ancestral catabolic functions, but acquired new pathways including ones involved in the degradation of tryptophan and lysine, and several peptidases (Table S2). A hallmark of the *Deinococcus* lineage is the presence of two predicted genes for malate synthase, an enzyme of the glyoxylate bypass which converts isocitrate into succinate and glyoxylate, allowing carbon that enters the TCA cycle to bypass the formation of α-ketoglutarate and succinyl-CoA [12]. It has been proposed that the strong upregulation of the glyoxylate bypass observed in *D. radiodurans* following irradiation facilitates recovery by limiting the production of metabolism-induced reactive oxygen species (ROS) [46]. Dgeo_2616/DRA0277 is the malate synthase ortholog present in the *Thermus* lineage, but the second predicted deinococcal malate synthetase (Dgeo_2611/DR1155) is unique and only distantly related to homologs in other bacteria. Although the two predicted deinococcal malate synthetases could have similar functions, the genomic context of Dgeo_2611/DR1155 indicates otherwise; Dgeo_2611/DR1155 are both located in a predicted operon with two cyclic amidases of unknown biochemical function.

In a broader context, the present reconstruction indicates that many expanded families of paralogous genes in *D. radiodurans* proliferated before the emergence of the common ancestor of the *Deinococci*, but the expansions were not present in the ancestor of the *Deinococcus-Thermus* group (Table 2). Such *Deinococcus*-specific expanded families include the Yfit/DinB family of proteins, acetyltransferases of the GNAT family, Nudix hydrolases, α/β superfamily hydrolases, calcineurin family phosphoesterases, and others. Many of these expansions are for predicted hydrolases, phosphatases in particular, but their substrate specificities are either unknown or the affinity of known substrates is extremely low [47]. It has been proposed, therefore, that the majority of these predicted enzymes perform cell-cleaning functions including degradation of damaged nucleic acids, proteins and lipids, and/or other stress-induced cytotoxins [47]. The global proliferation of these enzymes in the *Deinococcus* lineage (Table S3) supports the acquisition of chemical stress-resistance determinants early in its evolution; and the independent proliferation of determinants within these deinococcal species (*e.g*., calcinurin phosphatses, Figure S5) might represent secondary adaptations to specific stress environments. In summary, these findings indicate that the *Deinococcus* stress-resistance phenotypes evolved continuously, both by lineage-specific gene duplications and by HGT from various sources (Table S3, S4 and S5) [21].

**Table 2:** Ancestral expansions: paralogous gene families expanded in the *Deinococcus* lineage (DD) versus the *Thermus* lineage (TT) ancestors

Description	COG numbers	Number of tdCOGs: in DD only/in TT only/in TT and DD combined	Number of proteins DG/DR/TT(average)
**MutT-like phosphohydrolases (Nudix)**	COG0494 COG1051	3/2/6	12/18/8
**Calcineurin-like phosphoesterase**	COG0639 COG1408 COG1768 COG1692	7/0/4	12/11/4
**Lipase-like alpha/beta hydrolase**	COG0596 COG1073	6/0/6	13/16/5.5
**Subtilisin-like protease**	COG1404	2/0/4	7/10/3
**Acetyltrasferases GNAT family**	COG0454 COG1670	12/0/7	22/33/7
**DinB family (DNA damage and stress inducible proteins)**	COG2318 no COG	7/0/2	9/13/2

### Individual *Deinococcus* Species: Gene-Gain and Gene-loss

**Figure 3:**
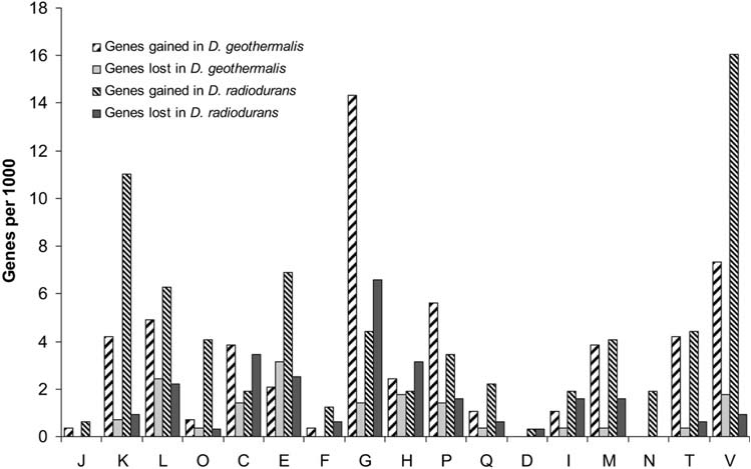
Gene-gain and gene-loss for different functional groups for *D. radiodurans* and *D. geothermalis.* Designations of functional groups (from the COG database): J–Translation, ribosomal structure and biogenesis; K–Transcription; L–DNA replication, recombination and repair; D–Cell division and chromosome partitioning; O–Posttranslational modification, protein turnover, chaperones; M–Cell envelope and outer membrane biogenesis; N–Cell motility and secretion; P–Inorganic ion transport and metabolism; T–Signal transduction mechanisms; C–Energy production and conversion; G–Carbohydrate transport and metabolism; E–Amino acid transport and metabolism; F–Nucleotide transport and metabolism; H–Coenzyme metabolism; I–Lipid metabolism; Q–Secondary metabolites biosynthesis, transport and catabolism; V–genes involved in stress response and microbial defense.

The comparison of gene-gain and gene-loss events in the *D. radiodurans* and *D. geothermalis* lineages reveals numerous differences, many of which correlate with their distinct metabolic phenotypes (Figure 3).

#### D. geothermalis

The most notable, distinctive feature of *D. geothermalis* is a greater abundance of genes for sugar metabolism enzymes, which could have been acquired after the divergence of the two *Deinococci*. The largest group within this set of genes is predicted to be involved in xylose utilization, needed for growth on plant material. D-xylose, which forms xylan polymers, is a major structural component of plant cell walls [48], and the presence of genes for aldopentose (xylose)-degradation explains why *D. geothermalis* is a persistent contaminant in paper mills [8]. Specifically, *D. geothermalis* contains genes encoding xylanases (Dgeo_2723; Dgeo_2722), an ABC-type xylose transport system (Dgeo_2699-Dgeo_2703), xylose isomerase (Dgeo_2375, Dgeo_2692, Dgeo_2693, Dgeo_2826), and xylose kinase (Dgeo_2691). Several of the genes that encode enzymes of xylose metabolism form paralogous families (Table S4), most of which form a cluster on the megaplasmid DG574 (Dgeo_2703-Dgeo_2687), which also contains two gene clusters predicted to be involved in carbohydrate utilization (Dgeo_2669-Dgeo_2693, Dgeo_2832-Dgeo_2812). By comparison, there are no large clusters of functionally related genes on the *D. geothermalis* chromosome; approximately 80 and 120 encoding proteins involved in sugar-metabolism were identified on DG574 and the chromosome, respectively. The putative xylose metabolism functions of *D. geothermalis* appear to represent an expansion of a pre-existing, broad and diverse set of functions underlying the saccharolytic phenotypes of all *Deinococci* [7], [11], [49], [50]. In contrast, *D. radiodurans* has a proteolytic lifestyle, where a loss of various amino acid biosynthetic pathways (Figure 3) [51] was accompanied by a gain of several predicted peptidases (DR0964, DR1070, DR2310, DR2503) and a urease system (DRA0311-DRA0319) [27]. Thus, the evolutionary processes underlying the emergence of extreme resistance in *Deinococci* appear not to be dependent on a particular set of genes for sugar- or nitrogen-metabolism. In summary, these findings support that DG574 is essential to the natural growth modes of *D. geothermalis*, which is a proficient saccharolytic organism [7], [49], [50] and strengthen the case that the megaplasmids in the *Deinococcus-Thermus* group are major receptacles of horizontally acquired genes, as proposed previously [27].

Further supporting the notion that a distinct set of metabolic genes is not a prerequisite for high levels of radioresistance, there are patent differences between sulfate and energy metabolism in *D. geothermalis* and *D. radiodurans*. In agreement with previously published results [7], [11], [51], the prototrophic *D. geothermalis* has orthologs of the *nadABCD* genes that are required for nicotinamide adenine dinucleotide (NAD) biosynthesis, whereas the auxotrophic *D. radiodurans* lacks these genes and is dependent on an exogenous source of this coenzyme [21], [51]. Another example illustrating the relationship in *D. radiodurans* between gene-loss and its growth requirements is that of cobalamine (vitamin B12). Whereas *D. geothermalis* and *T. thermophilus* are not dependent on B12 in minimal medium, *D. radiodurans* can utilize inorganic sulfate as the sole source of sulfur only when vitamin B12 is present [52]. Conversely, *D. geothermalis* has lost several genes for enzymes of protoheme biosynthesis (HemEZY) [53], which in *D. geothermalis* likely yields siroheme under the microaerophilic conditions which predominate at the T_opt_ of *D. geothermalis*; the solubility of dioxygen in water at 50°C is significantly lower than at 32°C, the T_opt_ of *D. radiodurans*.

There are also important differences between the systems for enzymes implicated in energy transformation in *D. geothermalis* and *D. radiodurans*. The *D. geothermalis* chromosome encodes two heme-copper cytochrome oxidases of types ba3 and caa3 [54]; and a cytochrome bd ubiquinol oxidase system (Dgeo_2707-Dgeo_2704), known to be expressed under oxygen-limiting conditions [55], is encoded by DG574. In contrast, *D. radiodurans* encodes only the caa3 oxidase system (DR2616-DR2620), which apparently was present in the *Deinococcus*-*Thermus* common ancestor. Furthermore, *D. geothermalis* encodes genes for proteins that comprise an assimilatory nitrite NAD(P)H reductase and a molybdopterin-cofactor-dependent nitrate reductase system (Dgeo2392-Dgeo_2389), which also is known to be expressed under anaerobic conditions [56], [57]; and *D. geothermalis* encodes several predicted multi-copper oxidases (Dgeo_2590, Dgeo_2559, Dgeo_2558) that are not present in *D. radiodurans* and are most similar to homologs from nitrogen-fixing bacteria. Since nitrogen fixation in *D. geothermalis* has not yet been studied, the possibility remains that these enzymes are involved in dissimilatory anaerobic reduction of nitrate or nitrite [58], [59]. *D. geothermalis*, but not *D. radiodurans*, also encodes a formate dehydrogenase, which is related to nitrate reductase and has a possible role in energy transfer under anaerobic conditions [60].

#### D. radiodurans

In general, the evolutionary trends in *D. radiodurans* lineage appear to mimic closely those of the *Deinococcus* lineage, which is evident from the analysis of expanded families of paralogous genes (Table S5). In particular, proliferation of genes for the Yfit/DinB family, Nudix enzymes, acetyltransferases of the GNAT superfamily, and the α/β hydrolase superfamily was observed (Table 2). Plausible resistance-related functions readily can be proposed for these and other expanded families of deinococci. For example, hydrolases might degrade oxidized lipids; Yfit/DinB proteins might be involved in cell damage-related pathways [21]; subtilisin-like proteases might degrade proteins oxidized during irradiation [17], [61]; and the Nudix-related hydrolase, diadenosine polyphosphatase (ApnA), yields adenosine, a molecule that has been implicated in cytoprotection from oxidative stress and radiation [62], [63].

Several families expanded in *D. radiodurans* are predicted to possess functions potentially relevant to stress response, but are not conserved in *D. geothermalis*; most likely, non-conserved families can be disqualified as major contributors to the extreme IR and desiccation resistance phenotypes. Families that are specifically expanded in *D. radiodurans* include the TerZ family of proteins, which are predicted to confer resistance to various DNA damaging agents [64], [65]; secreted proteins of the PR1 family, whose homologs are involved in the response to pathogens in plants, and resistance to hydrophilic organic solvents in yeast [66], [67]; PadR-like regulators, which are implicated in the regulation of amino acid catabolism and cellular response to chemical stress agents and drugs [68]–[70]; TetR/AcrR transcriptional regulators, which are involved in antibiotic resistance regulation [40]; and KatE-like catalases, which would decompose hydrogen peroxide [71]–[73]. In contrast, there are family expansions which are shared by *D. radiodurans* and *D. geothermalis*, but have no obvious role in radiation or desiccation resistance. These include SAM-dependent metyltransferases (COG0500) and an uncharacterized family of predicted P-loop kinases (COG0645). In some bacteria, homologs of these kinases are fused to phosphotransferases that mediate resistance to aminoglycosides [74].

Since the IR-, UV- and desiccation-resistance profiles of *D. radiodurans* and *D. geothermalis* are identical (Figure 1) [5], the subset of stress response genes in *D. radiodurans* that are not unique, but exist in excess compared to *D. geothermalis* are unlikely to be required for extreme resistance either (Figure 3). This subset includes two Cu-Zn superoxide dismutases (SOD), a peroxidase, two HslJ-like heat shock proteins, and many genes implicated in antibiotic resistance (Table S5). Consistently, SodA and KatA of *D. radiodurans* can be disrupted with almost no loss in radiation resistance [75], and antibiotics have little effect on survival following irradiation provided corresponding antibiotic resistance genes are present [18], [76]–[79].

#### The Deinococcus lineage

Considerable independent gene-gain was detected in both *D. geothermalis* and *D. radiodurans* lineages in several other functional categories including transcriptional regulation, signal transduction, membrane biogenesis, inorganic ions metabolism, and to a lesser extent DNA replication and repair (Figure 3). In general, regulatory functions mirror the metabolic and stress-response-related differentiation of these two species outlined above. For instance, among the 12 genes for predicted transcriptional regulators that apparently were acquired in the *D. geothermalis* lineage, five are similar to ones known to be involved in the regulation of sugar metabolism in other bacteria, two of the RpiR family and three of the AraC family [80], [81]. By contrast, *D. radiodurans* has at least 25 unique genes for transcriptional regulators: three of the ArcR family; 16 of the Xre family; one of the CopG/Arc/MetJ family; and five of a species-specific expanded family reported previously [61] that likely is responsible for stress-response control [82]-[85]. Other potentially independent gains involve genes predicted to be involved in signal transduction systems. *D. radiodurans*, for example, encodes photochromic histidine kinase, a protein that has been extensively studied in *D. radiodurans* and plays a role in the regulation of pigment biosynthesis [86], [87], but is missing in *D. geothermalis*. Alternatively, *D. geothermalis* encodes a putative negative regulator of sigma E, a periplasmic protein of the RseE/MucE family (Dgeo_2271). So far, RseE/MucE-members have been detected only in proteobacteria, where it regulates the synthesis of alginate, an extracellular polysaccharide which plays a key role in the formation of biofilms [88]. *D. geothermalis*, however, likely does not produce alginate itself since it has no orthologs of the genes of the alignate pathway [89]. On the other hand, *D. geothermalis* has clusters of genes implicated in exopolysaccharide biosynthesis, with the most notable cluster located on DG574 (Dgeo_2671-Dgeo_2646). It seems likely that this cluster is involved in the biosynthesis of exopolysaccharides, which might facilitate biofilm formation in *D. geothermalis*, and the Dgeo_2271 protein could be a regulator of this process. Overall, *D. radiodurans* encodes approximately 470 unique, uncharacterized proteins, for which no function could be predicted, compared to approximately 286 such proteins in *D. geothermalis*. Thus, an additional 756 unique, uncharacterized genes of the *Deinococcus* lineage can be excluded from the pool of putative determinants of the extreme IR, UV and desiccation resistance phenotype.

### Reassessment of the Genetic Determinants of Radiation Resistance

#### Evolutionary Provenance of the Genomic Features Previously Implicated in the Radiation Resistance of D. radiodurans

Over the last two decades, extensive experimental and comparative-genomic analyses have been dedicated to the identification and evolutionary origin of the genetic determinants of radiation resistance in *D. radiodurans*. Early on, it became evident that the survival mechanisms underlying extreme radiation resistance in *D. radiodurans* probably were not unique. In 1994, for example, IR-sensitive *D. radiodurans pol*A mutants were fully complemented by expression of the *polA* gene from the IR-sensitive *E. coli* [4]; and in 1996, UV-sensitve *D. radiodurans uvrA* mutants were complemented by *uvrA* from *E. coli* [90], suggesting that these recombination and excision repair genes are necessary but not sufficient to produce extreme DNA damage resistance. Following the whole-genome sequencing of *D. radiodurans* in 1999 [16], comparative-genomic analysis revealed many distinctive genomic features that subsequently became the focus of high throughput experiments, including the analysis of transcriptome and proteome dynamics of *D. radiodurans* recovering from IR [46], [91], [92]. Surprisingly, the cellular transcriptional response to IR in *D. radiodurans* appeared largely stochastic, and mutant analyses confirmed that many of the highly induced uncharacterized genes were unrelated to survival. So far, those correlative studies have failed to produce a coherent, comprehensive picture of the complex interactions between different genes and systems that have been thought to be important for the resistance phenotype.

**Figure 4:**
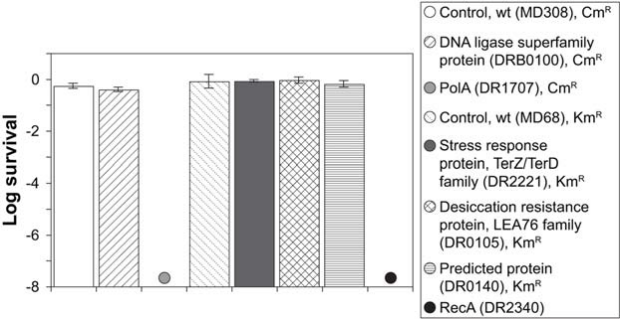
IR resistance of wild-type (ATCC BAA-816) and *D. radiodurans* mutants lacking orthologs in *D. geothermalis* (DSM 11300). Survival values following 9 kGy (^60^Co) are from three independent trials with standard deviations shown. The structure of the homozygous mutants DRB0100, DR2221, DR105 and DR0140 are presented in Figure S6.

The complete sets of orthologous genes in *D. radiodurans* and *D. geothermalis* are listed in Table S2. Within the subgroup of genes in *D. radiodurans* previously implicated in resistance by transcriptional induction following exposure to IR [46] (3 hours after irradiation and displaying more than a 2-fold induction), 45% have no othologs in *D. geothermalis*. This raises the possibility that many genes induced in irradiated *D. radiodurans* do not functionally participate in recovery, or that *D. geothermalis* carries a distinct set of resistance determinants. From the subgroup of putative resistance genes lacking counterparts in *D. geothermalis*, we constructed *D. radiodurans* knockouts of four representative genes: i) a ligase predicted to be involved in DNA repair (DRB0100) [46]; ii) a LEA76 desiccation resistance protein homolog (DR0105) [46]; iii) a predicted protein implicated in stress response (DR2221) [46]; and iv) a protein of unknown function (DR0140) [46]. Homozygous disruptions of each of these genes in *D. radiodurans* (Figure S6) had no significant effect on IR resistance (Figure 4).

By contrast, most of the genes whose mutants display radiation-sensitive phenotypes in *D. radiodurans* [4], [20], [46], [92], [93] are conserved in *D. geothermalis*. To date, 15 single-gene mutants of *D. radiodurans* have been reported to be moderately to highly radiation-sensitive; of these, 13 genes have orthologs in *D. geothermalis* (Table 3). The exceptions are DR0171 and DR1289, which encode the DNA helicase RecQ and a transcriptional regulator, respectively (Table 3). Remarkably, 10 of the 15 genes are conserved in other bacteria and are well-characterized components of DNA repair pathways. However, 5 of the 15 genes (DR0003, DR0070, DR0326, DR0423, DRA0346) are unique to the *Deinococcus* lineage, supporting the existence of at least a few novel resistance mechanisms.

**Table 3:** *D. radiodurans* genes implicated in radiation resistance

ADR gene	ADG ortholog	Homologs in other bacteria (COG number)	BReported Induction in Microarrays	CMutant phenotype	DReference	Description and Comments
DR0596 (ruvB)	Dgeo_0404	COG2255	+/+	+	[161] ; [46], [92]	Holliday junction resolvasome, helicase subunit, RuvB.
DR2340 (recA)	Dgeo_2138	COG0468	+/+	+	[46], [92]	RecA recombinase.
DR1289	-	COG0514	-/-	+	[127]	RecQ family of DNA helicase. The mutant is sensitive to IR, UV, H_2_O_2_ and MMC. In *D. geothermalis* there is a protein Dgeo_1226, which contains one Helicase superfamily C-terminal domain and one HDRC domain, which are similar to the corresponding domains of DR1289, but not the complete DR1289 ortholog.
DR1771 (uvrA)	Dgeo_0694	COG0178	+/+	+	[46], [92]	Excinuclease ATPase subunit, UvrA.
DR2275 (uvrB)	Dgeo_1890	COG0556	+/+	n/a	[46], [92]	Helicase subunit of the DNA excision repair complex, UvrB.
DR1913 (gyrA)	Dgeo_1016	COG0188	+/+	n/a	[46], [92]	DNA gyrase (topoisomerase II) A subunit.
DR0906 (gyrB)	Dgeo_0546	COG0187	+/+	n/a	[46], [92]	DNA gyrase (topoisomerase II) B subunit.
DR2220 (terB)	-	COG3793	+/+	n/a	[46], [92]	Tellurium resistance protein TerB.
DR2224 (terZ)	-	COG2310	+/-	n/a	[46], [92]	Tellurium resistance protein TerZ/TerD.
DR2221	-	COG2310	-/-	-	[46]; This work	Tellurium resistance protein TerZ/TerD.
DR2338 (cinA)	Dgeo_2136	COG1058/COG1546	+/-	n/a	[46], [92]	CinA ortholog, MoeA family.
DR2339 (ligT)	Dgeo_2137	COG1514	+/-	n/a	[46], [92]	2′→5′ RNA ligase, LigT.
DR1262 (rsr)	-	Bacteria Eukarya	+/-	-	[92]	Ro-like RNA binding protein.
DR1114	Dgeo_0505	COG0071	+/-	n/a	[92]	Molecular chaperone (small heat shock protein).
DR1709	Dgeo_0709	COG1914	+/-	n/a	[92]	NRAMP family membrane transporter.
DR0003 (ddrC)	Dgeo_0047	-	+/+	+	[46], [92]	Uncharacterized conserved protein, two low-complexity regions.
DR0070 (ddrB)	Dgeo_0295	-	+/+	+	[46], [92]	Uncharacterized conserved protein.
DR0167 (IrrE)	Dgeo_0395	COG2856	-/-	+	[108]	Regulatory Zn-dependent protease fused to HTH transcriptional regulator domain.
DR0194 (ddrE)	Dgeo_1282	COG2738	+/-	+	[92]	Zn-dependent protease, HTPX superfamily.
DR0219 (ddrF)	-	-	+/+	n/a	[46], [92]	Predicted protein.
DR0227 (ddrG)	-	-	+/-	n/a	[92]	Predicted protein, probably secreted.
DR0326 (ddrD)	Dgeo_2186	-	+/NA	+	[92]	Predicted low-complexity protein.
DR0423 (ddrA)	Dgeo_0977	COG4712	+/-	+	[46], [92]; [98]	Predicted DNA single-strand annealing protein, containing HHH motif, Rad42/Rad22/RecT/erf family.
DR0438 (ddrH)	-	-	+/-	n/a	[92]	Uncharacterized conserved protein, probably secreted.
DR0659 (frnE)	Dgeo_2073	COG2761	+/-	n/a	[92]	Uncharacterized DsbA-like thioredoxin fold protein.
DR0997 (ddrI)	Dgeo_1015	COG0664	+/-	n/a	[46], [92]	HTH transcription factor, CAP family.
DR1263 (ddrJ)	-	COG3236	+/+	n/a	[46], [92]	Uncharacterized protein conserved in bacteria.
DR1264 (ddrK)	-	-	+/+	n/a	[46], [92]	Predicted protein.
DR1439 (ddrL)	-	COG2010	+/-	n/a	[92]	Cytochrome C-related, CXXC motif.
DR1440 (ddrM)	Dgeo_0089	COG2217	+/NA	n/a	[92]	Cation-transporting ATPase.
DR2441 (ddrN)	Dgeo_0078	COG1670	+/-	n/a	[46], [92]	NH2-acetyltransferase.
DR2574 (ddrO)	Dgeo_0336	COG1396	+/+	n/a	[46], [92]	HTH transcription factor, phage type.
DRA0346 (pprA)	Dgeo_2628	-	+/+	+	[46], [92]	PprA protein, involved in DNA damage resistance mechanisms.
DRB0100 (ddrP)	-	Bacteria Archaea Eukarya	+/+	-	[46], [92] ; This work;	Homolog of eukaryotic DNA ligase III.
DRB0141 (hicB)	-	COG4226	+/-	n/a	[46], [92]	HicB family protein.
DR0171 (irrI)	-	Bacteria Archaea	-/+	+	[162] [46]	HTH transcriptonal regulator, specific for DR.
DR0690	Dgeo_2058	COG3569	-/-	n/a	[21]	Topoisomerase IB.
*DR1790*	-	*COG3386*	-/-	n/a	[21]	Yellow protein (*Drosophila*) or royal jelly protein (honey bee).
DR0166	Dgeo_0394	COG4281	-/-	n/a	[21]	Acyl-CoA-binding protein, ACBP.
DR1372	Dgeo_1551	Archaea Bacteria Eukarya	-/-	n/a	[21]	LEA14-like desiccation-induced protein.
*DRB0118*	*Dgeo_0097 Dgeo_1323*	Archaea Bacteria *Eukarya*	-/-	-	[21]; [163]	Desiccation-induced protein. The mutant is resistant to radiation but sensitive to desiccation.
DR1172	Dgeo_1473 Dgeo_1798	Bacteria Eukarya	-/-	-	[21]; [163]	LEA76/LEA26-like desiccation-induced protein. The mutant is resistant to radiation but sensitive to desiccation.
DR0105	-	Bacteria Eukarya	-/-	-	[21]; This work	LEA76/LEA26-like desiccation-induced protein.
DR0140	-	-	-/+	-	[21]; This work	Hypothetical protein.
*DR2209*	*Dgeo_0361*	*COG1718*	-/-	n/a	[21]	Protein kinase of RIO1 family.
DRA0145	-	COG5534	-/-	n/a	[21]	Peroxidase.
*DRA0339*	*Dgeo_2857*	*COG3483*	-/-	n/a	[21]	Tryptophan-2,3-dioxygenase.
DRA0338	Dgeo_1534	COG3844	-/-	n/a	[21]	L-kynurenine hydrolase.
DR0566	Dgeo_2026	COG2947	-/-	n/a	[21]	Homolog of a tymocyte protein cThy28kD.
DR0376	-	COG4636	-/-	n/a	[21]	Uncharacterized protein, uma2.
DR0467	Dgeo_1609	COG1796/COG1387	-/-	+	[134]	DNA polymerase of the X family with C terminal PHP hydrolase domain.
DR0189	Dgeo_1248	COG0353	-/-	-	[164]	RecR, the mutant is sensitive to DNA interstrand cross-linking agents but resistant to UV and IR.
DR1477	Dgeo_1194	COG0497	-/-	+	[165]	RecN.
DR1707	Dgeo_1666	COG0258/COG0749	-/-	+	[166]; [167]	DNA Polymerase A, PolA.
DRA0074	-		-/-	-	[168]; [169]	Diverged LexA homolog. Has a distinct DNA binding domain. Its mutant is slightly more resistant to IR.
DRA0344	Dgeo_1366	COG1974	-/-	-	[168]	LexA ortholog.
DR2444			-/-	-	[170]	PLP-binding enzyme fused to HRD domain.

A Abbreviations: DR, D. radiodurans; DG, D. geothermalis.

B Induction in DR whole-genome microarrays reported by Tanaka et al [92] versus DR microarray results by Liu et al [46]; +, induced; −, not induced; NA, microarray result is not available.

C Mutant phenotype: +, IR sensitive; −, IR resistant; n/a, not applicable. Corresponding mutant in D. radiodurans reported as referenced.

D References include original papers where the gene was inferred to be involved in radiation resistance or the corresponding mutant of the gene has been studied.

**Figure 5:**
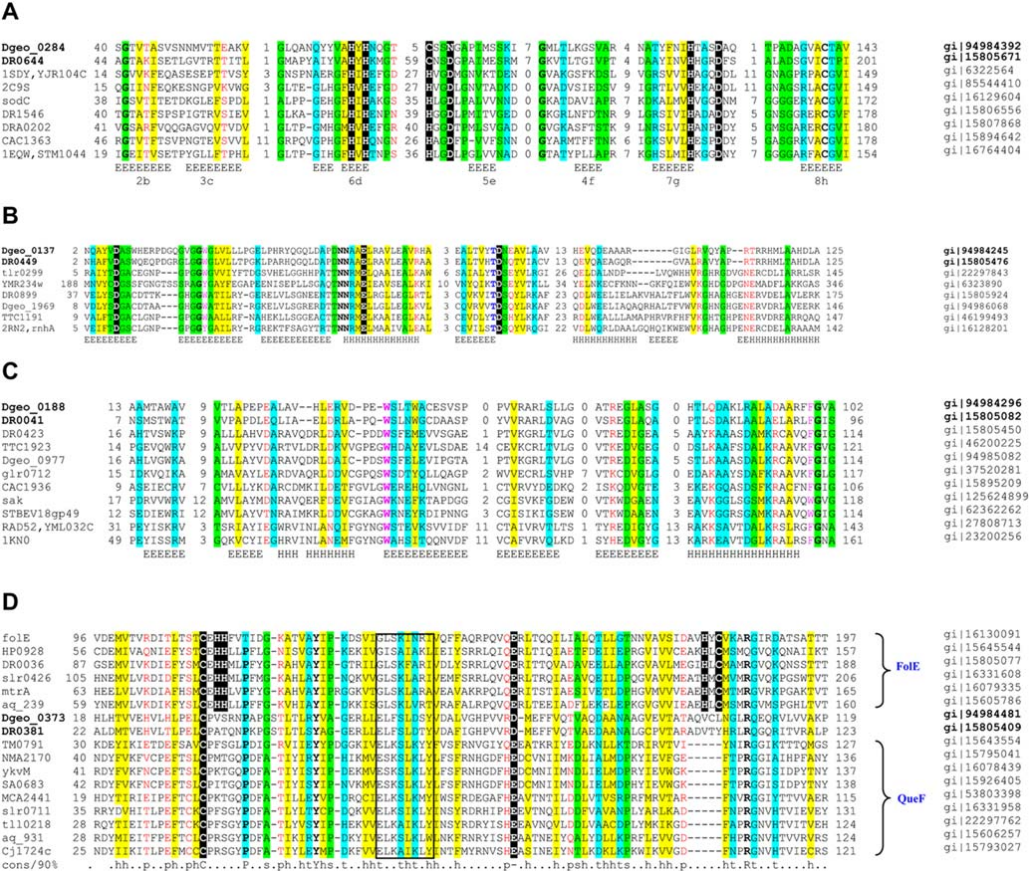
Multiple alignments of selected families conserved in two *Deinococcus* species. The multiple alignments were constructed for selected representative sets of sequences by the MUSCLE program [154]. Where necessary, alignments were modified manually on the basis of PSI-BLAST outputs [94]. The positions of the first and the last residue of the aligned region in the corresponding protein are indicated for each sequence. The numbers within the alignment refer to the length of inserts that are poorly conserved between all the families. Secondary structure elements are denoted as follows: E-β-strand; and H-α-helix. The coloring scheme is as follows: predominantly hydrophobic residues are high-lighted in yellow; positions with small residues are in green; positions with turn-promoting residues are in cyan; positions with polar residues are in red; hydroxyl-group containing residues are in blue; aromatic residues are in magenta; and invariant, highly conserved groups are in boldface. A, DR0644-Dgeo_0284 conserved pair of orthologs belong to the copper/Zinc superoxide dismutase family; shaded letters refer to amino acids that play an important role in the Cu^2+^/Zn^2+^ coordination environment and in the active site region. The bottom line shows the correspondence between the most conserved regions corresponding to the β-stand structural core and conserved in most family members as denoted in Bordo et al [157]. B, Dgeo_0137-DR0449 are highly specific for the *Deinococcus* lineage proteins that have an RNAse H-related domain. Catalytic residues conserved in the RNAse H family are shaded. Secondary structure elements are shown for *E. coli* RNase HI (PDB:2rn2). C, DR0041-Dgeo_0188 is another conserved pair (DdrA-related) of proteins belonging to the Rad52 family of DNA single-strand annealing proteins [97]. Secondary structure elements are shown for human RAD52 (PDB:1KN0) [158]. *sak* is a phage gene described previously [159]; D, DR0381-Dgeo_0373 are diverged homologs of NADPH-dependent nitrile reductase (GTP cyclohydrolase I family) that might be involved in nucleotide metabolism. The conserved Cys and Glu found in the substrate binding pocket of both protein families and specific zinc-binding and catalytic residues in the FolE family are shaded. The QueF family motif is boxed. Other catalytic residues in FolE not found in QueF are in yellow. Genbank Identifier (gi) numbers are listed on the right.

Given that the two *Deinococcus* species are equally resistant to IR (Figure 1A), genes dedicated specifically to the extreme radiation/desiccation response are expected to belong to the set of tdCOGs. *D. radiodurans* and *D. geothermalis* share 231 tdCOGs that are relatively uncommon in other prokaryotes, and 63 of these are unique to the *Deinococcus* lineage. Using the most sensitive methods available to predict function, we reanalyzed these tdCOGs by using a remote sequence similarity search, and genomic context analysis [94]–[96]. Interpretation of such analyses, however, is constrained by the complexity and ambiguities inherent in the approach, and by the knowledge base. In contrast, many cytosolic proteins (*e.g*., RecA, PolA, SodA and KatA) are known to be intimately involved in resistance, so we present functional predictions for 50 genes (Table S6). Among the predictions for cytosolic proteins, several are new and potentially relevant to resistance. For example, DR0644 (Figure 5A) is predicted to be a distinct Cu/Zn superoxide dismutase that could defend against metabolism-induced oxidative stress during recovery (Table S7); and DR0449 (Figure 5B) is a divergent member of the RNAse H family that is fused to a novel domain, a combination that is currently unique to *Deinococcus*. Other functional insights were for DR0041/Dgeo_0188, that is a paralog of DR0432 (DdrA) (Figure 5C); and a member of the RAD22/Rad52 family (Figure 5C) of single-stranded annealing proteins [97], that yields a moderately sensitive phenotype in *D. radiodurans* upon disruption [98]. Interestingly, the radiation-sensitive *T. thermophilus* encodes a homolog of DdrA (TTC1923), indicating that this protein had an ancestral role that was not directly related to radiation resistance. Notably, we continue to find proteins in *Deinococcus* species which are only remotely similar to well-characterized enzymes in other organisms, and it is difficult to predict their role in the cell or radiation resistance. For example, we have identified a protein that is conserved in both *D. geothermalis* and *D. radiodurans* and is distantly related to enzymes of the QueF/FolE family, which are involved in queuosine/folate biosynthesis (Figure 5D), but their role in the *Deinococci* remains undefined. Collectively, these results support the conclusion that many genes that are significantly induced in irradiated *D. radiodurans* are not involved in recovery (Table 3). Thus, the genome of *D. geothermalis* is a resource of major importance in delineating a reliable minimal set of resistance determinants, by corroborating those that are conserved and ruling out those which are unique.

#### Delineation of the Deinococcus Radiation Response Regulon

**Figure 6:**
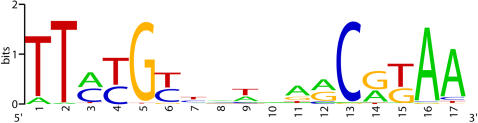
Sequence signature of a predicted site of a radiation response regulator. Four different nucleotides are shown by four letters (A, G, C, T) in different colors. The height of the letter is proportional to its contribution to the information content in the corresponding position of the multiple alignment used for “sequence logo” construction. The figure was constructed by the “sequence logo” program described previously [160].

A potential radiation-desiccation response regulon and the corresponding regulator common to *D. radiodurans* and *D. geothermalis* were identified using the approach developed by Mironov et al [99], [100]. In the search for such a regulator, we used a training-set comprised of sequences flanking *D. radiodurans* genes that were strongly upregulated by IR, and for which the corresponding mutants were radiosensitive (Table 3) [92]. The upstream regions of several genes from the training set (DR0326, *ddrD*; DR0423, *ddrA*; DRA0346, *pprA*; DR0070, *ddrB*) revealed a strong palindromic motif, designated the radiation/desiccation response motif (RDRM). Using a positional weight matrix, the RDRM was used to generate the initial profile and to scan the entire *D. radiodurans* genome. This genome survey picked up a similar motif in the upstream regions of other genes upregulated after irradiation [92]. The upstream regions with the highest scores (DR0219, DR0906, DR1913 and DR0659) were then used to better define the RDRM, and the complete genomes of *D. radiodurans* and *D. geothermalis* were scanned with the updated motif. Using moderately relaxed parameters (Materials and Methods), approximately 120 genes in each of the *Deinococcus* genomes were selected by the screen. The final, most conservative prediction of the radiation/desiccation response (RDR) regulon consisted of two groups: (i) a set of orthologous genes present in both *Deinococcus* species that contain the RDRM; and (ii) a set of unique genes of *D. radiodurans* that contain the RDRM and are upregulated during the recovery from irradiation [46], [92]. Since microarray data for *D. geothermalis* are not available, it was not possible to predict a set of unique RDRM-dependent genes for this species. Table 4 lists the set of genes predicted to comprise the regulon together with the corresponding RDRM sites (Figure 6). Collectively, the RDR regulon is predicted to consist of a minimum of 29 genes in *D. radiodurans* and 25 genes in *D. geothermalis*, contained within 20 operons in each species.

**Table 4:** The predicted radiation and desiccation resistance regulon of *Deinococci*

ADR gene	Position	Score	Site in DR	ADG ortholog	Position in DG	Score in DG	Site in DG	BTanaka et al	CLiu et al	Gene product name	Description and Comments
DR0070*	4	5.24	TTATGTTATTtACgTAA	Dgeo_0295	−27	5.24	TTATGTTATTtACgTAA	yes	yes	DdrB	Uncharacterized conserved protein
DR0099	−28	5.22	TTATGTcATTgACATAA	Dgeo_0165	−113	4.83	TTATGcTcTTgACgTAA	no	yes	Ssb	Single-stranded DNA-binding protein
DR0219*	−41	5.13	TTATGTTATatACgTAA	no				yes	yes	DdrF	Predicted protein
DR1913*	−116	5.02	TTAcGTgATTAACATAA	Dgeo_1016	−115	4.79	TTAcGccAAaAACATAA	yes	yes	GyrA	DNA gyrase (topoisomerase II) A subunit
					57	3.84	TTAcGcgATgAACgTgA				
DR1143	49	4.78	TTATGTTtTaAgCgTAA	no				no	yes		Similar to DR1142, but with a frameshift
DR0906*	−257	4.63	TTcTGTaAgagACgTAA	Dgeo_0546	15	4.85	TTAcGcTcATAACgTAA	yes	yes	GyrB	DNA gyrase (topoisomerase II) B subunit
DR0423*	−41	4.62	TTATGTctTgAcCgTAATTcTGTTcTaAACtaAA	Dgeo_0977	n/a			yes	no	DdrA	Predicted DNA single-strand annealing protein, containing a HHH motif, Rad42/Rad22/RecT/erf family
	−19	4.03									
DR0326*	−26	4.57	TTcTGcTAAaAACAgAA	Dgeo_2186	−26	4.81	TTcTGTcAAaAACAgAA	yes	no	DdrD	Predicted low complexity protein
DRA0346*	22	4.29	aTcTGTTcAgggCATAA	Dgeo_2628	−55	4.15	aTcTGTctAgggCATAA	yes	no	PprA	PprA protein, involved in DNA damage resistance mechanisms
DR2256	−180	4.29	TTcTGTctTTAcCggAA	Dgeo_2283	−213	3.78	TTcTGcTtcTggCATAt	no	yes	Tkt	Transketolase, Tkt
					−197	3.96	TTcTGTTgTTAcCggAA				
DR1039	−42	4.14	TTtcGcTcAgAACgTAA	Dgeo_1537	31	3.89	TTccGcccAcAACAgAAaaATGTctTgAAgggAA	no	no	DMutS	DNA mismatch repair ATPase MutS
					90	3.47					
DR1696	−17	3.92	aTATGcTcAcAACAgAA	Dgeo_1538				no	no	HexB/DMutL	DNA mismatch repair enzyme, Hexb/MutL
DR1289	−34	4.09	TTcTGcccAcAACgTAA	Dgeo_1226	−361	3.89	TTccGTccAcAgCAgAA	no	no	RecQ	RecQ helicase
DR1775	−40	4.09	TTAcGcTccTggCAgAA	Dgeo_0868	−49	3.5	TTATGccgccAACAgAA	no	yes	UvrD	UvrD Superfamily I helicase
DR2275	74	3.87	TTAcGcTgTgggCgTAA	Dgeo_1890	−87	4.79	TTATGTTtTTggCgTAA	no	yes	UvrB	Helicase subunit of the DNA excision repair complex, UvrB
DR0596	−25	3.84	TTtcGcaAATAgCgTAA	Dgeo_0404	−25	3.84	TTtcGcaAATAgCgTAA	yes	yes	RuvB	Holliday junction resolvasome, helicase subunit, RuvB
DR2338	−3	3.80	TTATGcTgcTAgCAgAA	Dgeo_2136	−3	4.23	TTATGcTtcTAgCAgAA	yes	yes	CinA	CinA ortholog, MoeA family, first gene in operon containing RNA ligase ligT and RecA
										LigT	
										RecA	
DR1771	−63	3.79	TTAcGcgccTgcCgTAA	Dgeo_0694	−70	3.6	TTAcGcgAAcAgCAgAA	yes	yes	UvrA	Excinuclease ATPase subunit, UvrA
DR2574	−149	3.75	TTcTGTatTgAcCgTAg	Dgeo_0336	−158	3.75	TTcTGTatTgAcCgTAc	yes	yes	DdrO	HTH transcription factor, phage type
DRA0151	−115	3.70	TTccGgatAgtgCggAA	Dgeo_2735	−102	3.55	TTccGggATatgCggAA	no	yes	HutU	Urocanate hydratase (and three more genes in the same operon for histidine degradation)
										HutH	
										HutI	
										HutG	
DR0659*	−29	3.65	TTATtTTcTaAACtgAt	Dgeo_2073	n/a			yes	no	FrnE	Uncharacterized DsbA-like thioredoxin fold protein
DR1921	61	3.56	TTccGTcATgcgCgTAc	Dgeo_0824	−8	3.56	TTccGTcATgcgCgTAc	no	no	SbcD	SbcD, DNA repair exonuclease
DR0171	−19	3.52	aTcTGgccTgtACtgAA	no				no	yes	IrrI	HTH transcriptional regulator
DR1262	−122	3.52	TTccGTctgTtgCgTcA	no				yes	no	Rsr	Ro-like RNA binding protein

A Abbreviations: DR, D. radiodurans; DG, D. geothermalis.

B Induction in whole-genome microarrays reported by Tanaka et al [92].

C Induction in whole-genome microarrays reported by Liu et al [46].

D In D. geothermalis, MutS and MutL are in the same operon, therefore RDRM information is shown only for Dgeo_1537 (the first gene in the operon).

* RDRM sites included in the final profile were used to scan the genomes of D. radiodurans and D. geothermalis.

The RDR regulon is dominated by DNA repair genes, including the recombinational repair proteins RecA and RecQ [101], [102]; the mismatch repair proteins MutS and MutL, that are located in one operon in *D. geothermalis*; and the UvrB and UvrC proteins, which are involved in nucleotide excision repair (Table 4). In addition, the predicted RDR regulon includes the transketolase gene. In bacteria, transketolase is a key enzyme of the pentose-phosphate pathway for carbohydrate metabolism and is known to be induced by a variety of stress conditions including cold shock, and mutagens that trigger the SOS response [103]. Moreover, the pentose-phosphate pathway in *D. radiodurans* is reported to facilitate DNA excision repair induced by UV irradiation and hydrogen peroxide (H_2_O_2_) [104]. The RDRM also precedes a conserved histidine catabolism operon [105]. Several bacterial biodegradative and related operons are known to be differentially induced in response to a decline in biosynthetic and energy-generating activities under oxidative stress [106]. For example, the TCA cycle in *D. radiodurans* is strongly down-regulated following irradiation [46], whereas the glyoxylate bypass of the TCA cycle, and the His operon are induced [46]. Several studies have provided direct evidence that survival of *D. radiodurans* following exposure to IR depends on a coordinated metabolic response and a high level of respiratory control [46], [107].

The regulation of gene expression in *D. radiodurans* during recovery from IR has been the subject of considerable investigation. Recently, it has been shown that the induction of *recA* in irradiated *D. radiodurans* is regulated by the IrrE/PprI protein [108], [109], which consists of two domains, a Xre-like HTH domain and a Zn-dependent protease. In both *D. radiodurans* and *D. geothermalis*, the *irrE* gene is located upstream of the folate biosynthesis operon, but appears to be regulated independently [110]. Since *recA* in *D. radiodurans* is strongly induced following irradiation [46], [111], it was surprising that the *irrE* gene of *D. radiodurans* was constitutively expressed, showing no post-irradiation induction [46], [92], [110]. Furthermore, the IrrE/PprI protein has an unusual domain structure and does not appear to bind the promoter region of *recA* or other induced genes [110].

Compared to radiosensitive bacteria, the regulatory mechanisms underlying the response to radiation in *D. radiodurans* are still poorly characterized. For example, the LexA-regulated SOS-dependent radiation response regulon of *E. coli* is well-defined [103], [112]–[115], but an equivalent system in *D. radiodurans* has not been identified. *D. geothermalis* has one *lexA* gene (DG1366) and *D. radiodurans* has two *lexA* paralogs (DRA0344, DRA0074). However, the *lexA* genes in *D. radiodurans* are not induced after irradiation, are not involved in RecA induction [116], and are not preceded by RDRM sites [46], [92]. Therefore, LexA is not a candidate for the role of the regulator of the *Deinococcus* RDR regulon. In the microarray experiments of Liu et al, several putative regulators were upregulated in *D. radiodurans* following exposure to 15,000 Gy [46]. In contrast, at lower doses (3,000 Gy), the *D. radiodurans* microarray experiments of Tanaka et al detected only one upregulated putative regulator (DdrO) (DR2574) [92]. An orthologous gene for DdrO is present in *D. geothermalis* (Dgeo_0336). DdrO is a Xre family protein and is the only *Deinococcus* gene for a predicted regulator that is preceded by a RDRM site (Table 4). This arrangement is common to many stress response regulators, e.g., the *lexA* genes of many species [117]. Thus, we propose that DdrO is the global regulator of the RDR regulon in the *Deinococcus* lineage.

### Impact of the comparative-genomic analysis of the two *Deinococcus* genomes on Resistance Models

In 1971, Moseley and Mattingly reported the first mutant analyses for *D. radiodurans* that showed that its recovery from radiation is dependent on DNA repair [118]. Subsequent research confirmed that DNA repair enzymes, which are central to recovery of irradiated bacteria in general, were key to *D. radiodurans* survival. Remarkably, several highly radiation-sensitive *D. radiodurans* DNA repair mutants were fully complemented by expression of orthologous genes from radiosensitive bacteria [4], [90], [119]–[121]. Thus, the extreme resistance phenotype appeared to be dependent, at least in part, on a conventional set of DNA repair functions [5], [17], [21]. Generally, this view has been supported by the analysis of the complete genome sequence of *D. radiodurans* [16], and subsequently, by whole-transcriptome and whole-proteome analyses for *D. radiodurans* recovering from IR [46], [91], [92]. Central to current models of extreme resistance are hypotheses that aim to reconcile the seemingly paradoxical findings that DNA repair proteins in *D. radiodurans* function extremely efficiently, yet appear structurally unremarkable, and often can be complemented by orthologs from radiosensitive bacteria. Within this conceptual framework, we examined the impact of the inferences on gene-gain and gene-loss derived from the comparative-genomic analysis of the two *Deinococcus* species on prevailing models of extreme radiation and desiccation resistance.

#### Hypothesis I: Chromosome Alignment, Morphology and Repeated Sequences Facilitate Genome Reassembly

*recA*-dependent homologous recombination occurs at hundreds of IR-induced DSB sites in *D. radiodurans* during recovery from 17.5 kGy IR [18], [76]–[79]. In *D. radiodurans*, the alignment of its multiple identical chromosomes is often tacitly assumed as the starting point for a given repair model, yet little is known about how, or even if, such chromosomal alignment occurs. The first model that considered this possibility in the recovery of *D. radiodurans* was published by Minton and Daly in 1995 [122]. The model built on the idea that alignment of identical chromosomes is a natural and early consequence of semi-conservative replication, where persistent chromosomal pairing was predicted to facilitate the ‘search for homology’ that precedes homologous recombination. The model made two major predictions: first, transmission electron microscopy (TEM) of chromosomal DNA from *D. radiodurans* should reveal evidence of structures linking chromosomes; and second, *recA*-dependent recombination between homologous DNA fragments inserted at widely separated genomic locations should show strong positional effects upon irradiation. Both predictions have been tested and refuted: no linking structures have been observed by TEM-based optical mapping [26], and molecular studies have shown high levels of recombination between homologous DSB fragments irrespective of their genomic origin [76]–[79], [122]. Thus, it has been concluded that IR-induced DSB fragments in *D. radiodurans* are mobile and that the structural form of its nucleoids does not play a key role in radioresistance. These conclusions were subsequently strengthened by cryoelectron microscopy of vitreous sections of *D. radiodurans* [123], [124], and by nucleoid morphology studies [5], [12], [24], [125].

The genome of *D. radiodurans* contains numerous, unusual, mosaic-type SNRs [16], [21], [29] which potentially could contribute to genome assembly by holding together homologous DSB pairs [26]. TEM optical mapping of *D. radiodurans* recovering from IR, however, showed that IR-induced DSB fragments in *D. radiodurans* were not linked [26]. Consistently, the present whole-genome comparison detected none of these repeats in *D. geothermalis*, nor any other expanded repeat families, including G-quadruplex sequences (Table 1) (Figure S3). We did not identify any unusual features in chromosome-binding proteins that are conserved in the two *Deinococcus* genomes compared to the orthologous proteins from other bacteria [21] (Table S7 and S8). Thus, our comparative analysis does not seem to support Hypothesis I. More broadly, there is currently no convincing experimental evidence supporting the idea that structural alignment, aggregation or organization of the *D. radiodurans* chromosomes has a significant effect on radiation/desiccation resistance. However, we cannot rule out the possibility that the genomes of sensitive bacteria have structural characteristics that predispose them to inefficient genome reassembly.

#### Hypothesis II: A Subset of Uncharacterized Genes Encode Functions that Enhance the Efficiency of DNA repair

In general, bioinformatic and experimental studies suggest that genome configuration and copy-number or the protection and repair functions of sensitive bacteria do not have unique properties that predispose them to DNA damage or inefficient DNA repair [5], [20], [21]. More specifically, chromosomes in sensitive and resistant bacteria are equally susceptible to IR-induced DSB damage [5], [19] and UV-induced base damage [126]; and DNA repair and protection genes of *T. thermophilus*, a radio-sensitive representative of the *Deinococcus*-*Thermus* group, and *E. coli* do not show obvious differences from their counterparts in *D. radiodurans* or *D. geothermalis* [5], [21], [27] (Table S8). Furthermore, several *E. coli* DNA repair genes, including *polA* and *uvrA*, have been shown to restore the corresponding radiation-sensitive *D. radiodurans* mutants to wild-type levels of *D. radiodurans* resistance [4], [90], [120]; and the products of interchromosomal recombination in *D. radiodurans* following irradiation are consistent with the canonical version of the DSB repair model [76]–[79]. It has been proposed that *D. radiodurans* uses a core set of conventional DNA repair enzymes in novel ways, where conventional repair activities are enhanced by as yet uncharacterized proteins. For example, Zahradka et al have recently proposed a model called extended synthesis dependent strand annealing (ESDSA) that utilizes PolA in an unprecedented way [22].

Under the ESDSA, DSB fragments formed in irradiated *D. radiodurans* are first subject to a 5′→3′ exonuclease resection mechanism that generates overhanging 3′ tails. A 3′ tail then invades a homologous DSB fragment derived from a different chromosomal copy, displacing the corresponding 5′ strand as a loop. Synthetic extension of the priming 3′ terminus might then proceed to the end of the invaded fragment, followed by annealing of the newly synthesized long 3′ extension with a complementary strand of another fragment engaged in ESDSA (Figure S7). For example, if the sequences of two priming fragments were *ABCD* and *GHIJ*, then a bridging and templating fragment could be *DEFG*, and the sequence of the assembled contig would be *ABCDEFGHIJ* [22]. The ESDSA model accounts for the formation of large, interspersed blocks of old and new DNA observed in repaired *D. radiodurans* chromosomes. Some aspects of the ESDSA model, however, are difficult to reconcile with earlier experimental findings for *recA*-independent single-stranded annealing (SSA) mechanisms in irradiated *D. radiodurans* [78] (Figure S7). Zharadka et al conceded that the SSA model is a potential alternative to ESDSA and could perhaps generate small blocks of old and new DNA [22], but pointed out that the *E. coli* PolA Klenow fragment, that lacks the 5′→3′ exonuclease, fully complements *D. radiodurans polA* mutants for resistance to γ-radiation. The present analysis shows that, although *D. radiodurans* and *D. geothermalis* do not encode *recBCE*, they both encode *recJ*, a putative 5′→3′exonuclease that could potentially provide nuclease activity missing in the Klenow fragment (Table S8).

The possibility that extreme resistance in *D. radiodurans* is determined by novel genes that enhance conventional repair functions has also been examined [20], [46], [98]. At least 12 genes of *D. radiodurans*, which were implicated in resistance by transcriptional profiling following IR, have been knocked out and the resulting mutants were characterized for IR resistance (Table 3). Remarkably, for most of the novel mutants, the IR resistances remained high [20], [46], [98], indicating that few of the uncharacterized genes, at least individually, makes a substantial contribution to the recovery of irradiated *D. radiodurans*. For example, the DR0423 protein has been reported to bind 3′ ends of single-stranded DNA molecules, perhaps, protecting 3′ termini generated by SSA or ESDSA from nuclease degradation. A DR0423 knockout mutant, however, retained approximately half of the wild-type level of IR resistance [92], [98]. To date, only a few of the uncharacterized genes selected for disruption analysis have contained the RDRM (Table 3 and 4).

At least three *Deinococcus* proteins involved in repair show features that stand out against the overall, “garden-variety” of bacterial repair systems. First, *D. radiodurans* encodes a protein (DR1289) of the RecQ helicase family, which contains three Helicase and RNase D C-terminal (HRDC) domains, whereas most of the other bacterial RecQ proteins have a single HRDC domain. A *D. radiodurans recQ* knockout mutant is sensitive to IR, UV, H_2_O_2_, and MMC, and it has been reported that all three HRDC domains contribute to resistance [127]. However, *D. geothermalis* has no ortholog of the *D. radiodurans* RecQ, but does encode the Dgeo_1226 protein that contains a helicase superfamily II C-terminal domain and a second HDRC domain that has high similarity to the corresponding domains of DR1289. Both DR1289 and Dgeo_1226 belong to the predicted resistance regulon (Table 4). A second exceptional case is RecA, the key repair protein that is required for homologous DNA recombinational repair following irradiation [20]. The DNA strand-exchange reactions promoted by the RecA proteins from all other bacteria studied to date are ordered such that the single-stranded DNA is bound first, followed by the double-stranded DNA. In contrast, the *D. radiodurans* RecA binds the DNA duplex first and the homologous single-stranded DNA substrate second [128]. It seems likely, however, that these unusual properties of RecA are ancestral to the *Deinococcus-Thermus* group. Indeed, most of the amino acid residues that are distinct in *Deinococcus* and could be responsible for the structural and functional differences between the RecA proteins of *Deinococcus* and other bacteria are also present in the RecA sequence of *Thermus* (Figure S8). In this context, early work by Carroll *et al* [111] reported that *E. coli* RecA did not complement an IR-sensitive *D. radiodurans recA* point-mutant (rec30) and that expression of *D. radiodurans* RecA in *E. coli* was lethal. More recently, however, it has been reported that *E. coli recA* can provide partial complementation to a *D. radiodurans recA* null mutant [121], and that *D. radiodurans recA* fully complements *E. coli recA* mutants [129]. This suggests that the *D. radiodurans* RecA protein is not as unusual as initially believed, but rather is more analogous to *polA* and *uvrA* of *D. radiodurans*, which can be functionally replaced by *E. coli* orthologs [4], [90], [93], [120]. A third example, the *Deinococcus* single-stranded binding protein (Ssb) has a distinct structure, with two OB-fold domains in a monomer, but this feature was apparently already present in the common ancestor of *Deinococcus/Thermus* group and therefore cannot be linked to radiation resistance directly [130].

It has been repeatedly proposed that nonhomologous end-joining (NHEJ) occurs in *D. radiodurans* [20], [131]–[136]. However, experiments specifically designed to test for the occurrence of NHEJ in *D. radiodurans* have shown that NHEJ of irradiation-induced DSB fragments is extremely rare, if not absent [78]. More recent work also supports this conclusion [22]. In the present and a previous study, we did not identify any orthologs of genes from other organisms that might encode NHEJ in *D. geothermalis* or *D. radiodurans* [21]. However, it cannot be ruled out that *Deinococcus* encodes a unique NHEJ system. For example, DRB0100 encodes an ATP-dependent ligase that contains domains that could potentially contribute to NHEJ, namely, a predicted phosphatase of the H2Macro superfamily and an HD family phosphatase and polynucleotide kinase [46], [92]. Furthermore, DRB0100 belongs to a set of three genes comprising a putative operon (DRB0098-0100) that is strongly induced by IR. A homozygous disruption of the DRB0100 gene, however, is fully IR-resistant (Table 3) (Figure 4), and genome comparison showed that *D. geothermalis* has no orthologs of DRB0100 or any functionally related operons. Despite the strong induction of DRB0100 following irradiation and the apparent relevance of the predicted function of this protein to *D. radiodurans* repair, DRB0100 appears not to contribute to resistance (Figure 4), and when purified, does not display DNA or RNA ligase activity *in vitro* [137]. These findings, therefore, reflect a broader paradox of *Deinococcus*: whereas computational analyses have revealed an increasing number of new proteins potentially involved in the extreme resistance phenotype, very few of the corresponding *D. radiodurans* mutants tested so far have had a significant effect on its IR resistance. The present work leads to further shrinking of the set of genes implicated as major contributors to the resistance phenotype by showing that many of the original candidates are not conserved between *D. geothermalis* and *D. radiodurans*. Thus, our comparative analysis appears to be inconsistent with Hypothesis II, and reinforces inferences from a growing body of experimental work on *Deinococcus* species, which support that these organisms rely on a relatively conventional set of DNA repair functions.

#### Hypothesis III: The level of Oxidative Protein Damage during Irradiation Determines Survival

Over the past decade, several observations have challenged the DNA-centered view of IR toxicity in eukaryotes and prokaryotes [5], [17], [23], [138], including (i) IR-induced bystander-effects in mammalian cells, defined as cytotoxic effects elicited in non-irradiated cells by irradiated cells, or following microbeam irradiation of cells where the cytoplasm but not the nucleus is directly traversed by radiation [139]; (ii) the genomes of radiation-sensitive bacteria revealed nothing obviously lacking in their repertoire of DNA repair and protection systems compared to resistant bacteria [12], [21]; and (iii) for a group of phylogenetically diverse bacteria at the opposite ends of IR resistance, the amount of protein damage, but not DNA DSB damage, was quantifiably related to radioresistance [5], [17]. Thus, while the etiological radicals underlying different oxidative toxicities appear closely related [140], the pathway connecting the formation of IR-induced ROS with endpoint biological damage is still not definitively established [23]. It has been proposed recently that proteins in IR-sensitive cells are major initial targets, where cytosolic proteins oxidized by IR might actively promote mutation by transmitting damage to DNA [141], and IR-damaged DNA repair enzymes might passively promote mutations by repair malfunction [17]. In comparison, Mn-dependent radioprotective complexes in IR-resistant bacteria [17] appear to protect proteins from oxidation during irradiation, with the result that enzymatic systems involved in recovery survive and function with great efficiency [17]. The proposed mechanism of extreme IR resistance requires a high intracellular Mn/Fe concentration ratio, where redox-cycling of Mn(II) complexes in resistant bacteria [5], [17] scavenge a subset of IR-induced ROS that target proteins. Because the formation of ROS during irradiation is extremely rapid [140], an intracellular protection system that is ubiquitous, but not highly dependent on the induction of enzymes, stage of growth, or temperature over a range at which cells are metabolically active, could provide a selective advantage to the host in diverse settings.

**Figure 7:**
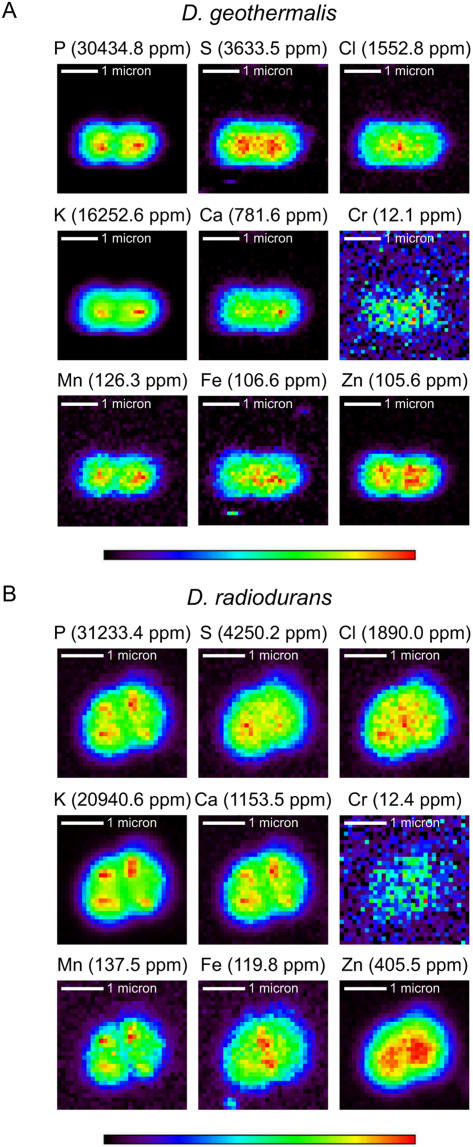
X-ray fluorescence (XRF) microprobe element distribution maps [142]. A, *D. geothermalis* (diplococcus). B, *D. radiodurans* (tetracocus). Cells were harvested from mid-logarithmic cultures in undefined rich medium, imaged, and quantified as described previously [17]. The element distribution images are plotted to different scales designated by a single color-box, where red represents the highest concentration and black the lowest. ppm values in parentheses next to the element symbol correspond to red. XRF microprobe analysis measurements were made at beamline 2ID-D at the Advanced Photon Source, Argonne National Laboratory as described recently [17].

Since high intracellular Mn/Fe ratios have been implicated in radiation and desiccation resistance [5], [12], [17], [23], we examined the intracellular concentrations and distributions of Mn, Fe and seven other elements in *D. geothermalis* compared to *D. radiodurans*, determined by x-ray fluorescence (XRF) microscopy (Figure 7) [142]. The XRF analyses showed that the intracellular levels of Mn and Fe and their locations in *D. geothermalis* are essentially the same as *D. radiodurans* [17], but very different from the concentrations and distributions in IR-sensitive bacteria [5], [142]. In this context, both *D. radiodurans* and *D. geothermalis* encode the Mn(II) transporter Nramp (DR1709) and a putative Mn-dependent transcriptional regulator TroR (DR2539) [5], but lack many genes for Fe homeostasis common in other bacteria, including for siderophore biosynthesis (COG3486, COG4264, COG4771) and Fe transport (COG1629, COG0810) (Table S9) [12]. Consistently, *D. radiodurans* and *D. geothermalis* do not secrete siderophores (Figure S9), the *nramp* gene of *D. radiodurans* is essential and could not be disrupted, and the Fe uptake regulator (Fur) in *D. radiodurans* was dispensable (Figure S10); a system for gene disruption in *D. geothermalis* has not been developed. Other recent work that has strengthened the argument for a critical role of Mn(II) in the extreme resistance phenotypes of *D. radiodurans* includes *in vitro* studies of Heinz and Marx [143]. They have shown that purified *D. radiodurans* PolA and *E. coli* PolA can bypass certain forms of IR-induced DNA damage during replication in the presence but not in the absence of 1 mM Mn(II), and suggested that Mn(II) ions might serve as important modulators of enzyme function [143]. In summary, we conclude that our genome comparison (Table S9), gene knockout (Figure S10) and element analyses (Figure 7) appear to be consistent with Hypothesis III, whereby survival is facilitated by systems which regulate the concentration and distribution of intracellular Mn and Fe. Based on recent work, it appears that the presence of globally-distributed intracellular nonenzymic Mn(II) complexes in resistant bacteria facilitates recovery by preventing a form of IR-induced Fe-catalyzed protein oxidation known as carbonylation [17].

### Conclusions

Based on their identical radiation resistance characteristics and close phylogenetic relationship, *D. geothermalis* and *D. radiodurans* are well-suited to defining a minimal set of conserved genes that could be responsible for extreme resistance. The two major findings of this analysis are (i) the characterization of the evolutionary trends that led to the emergence of extreme stress resistance in the *Deinococcus* lineage, in particular the finding that many families of paralogous genes, previously shown to be expanded in *D. radiodurans*, proliferated before the emergence of the common ancestor of the *Deinococci*, but were not present in the ancestor of the *Deinococcus-Thermus* group (Table 2); and (ii) delineation of a set of genes that comprise the predicted *Deinococcus* radiation and desiccation response regulon, which defines a new subgroup of targets for investigation in the *Deinococci* (Table 4). These findings have strengthened the view that *Deinococci* rely more heavily on the high efficiency of their detoxifying systems, including enzymic and nonenzymic ROS scavengers, than on the number and specificity of their DNA repair systems (Table 3). Our findings, however, do not rule out the possibility that the exceptional efficiency of DNA repair processes in both *Deinococcus* species is, at least in part, due to modifications of a set of universal repair genes. With respect to the impact of the whole-genome sequence of *D. geothermalis* on prevailing models of extreme IR resistance, the results of the comparative analysis weaken the arguments for a role of higher-order chromosome alignment structures (Hypothesis I); more clearly define and substantially revise downward the number of uncharacterized genes that might participate in DNA repair and contribute to resistance (Hypothesis II); and are consistent with the notion of a predominant role in resistance of systems involved in cellular protection and detoxification (cell-cleaning) (Hypothesis III).

In the hierarchy of DNA lesions caused *in vivo* by radiation, DSBs are the least frequent ones, but the most lethal [140]. Since the number of genomic DSBs induced by a given dose of IR in resistant and sensitive bacteria is about the same [5], [19], a legitimate question is whether resistant and sensitive bacteria are also equally susceptible to DNA base damage. Setlow and Duggan showed that *D. radiodurans* and *E. coli* are similarly susceptible to DNA thymine-dimers caused by UV [126]. For IR and UV, the differences reported in resistance of DNA to radiation damage are not nearly sufficient to account for the relative resistance of *D. radiodurans*. Thus, it seems surprising that the recombination and excision repair systems of *D. geothermalis* and *D. radiodurans* did not proliferate compared to sensitive cells [5]. The DNA repair and damage signaling systems of these radiation reisistant bacteria appear quantitatively and qualitatively even less complex and diverse than those reported for some sensitive bacteria [5], [144]. Instead, the stress-resistance phenotypes of the *Deinococcus* lineage appear to have evolved progressively by accumulation of cell-cleaning systems which eliminate organic and inorganic cell components that become toxic under radiation or desiccation [12], [23], [46], [92]. In *D. geothermalis* and *D. radiodurans*, this form of cell-cleaning appears to manifest itself as protein protection during exposure to IR [17] or desiccation [JFK, EKG, MJD, unpublished], where proteins in *Deinococci* are substantially more resistant to oxidative damage than proteins in sensitive bacteria [17]. Our finding that many genes in the predicted *Deinococcus* damage response regulon are the same as those found in SOS regulons of sensitive bacteria, but are regulated differently, is easily reconciled with the idea that enzymes and biochemical pathways in resistant bacteria survive and function more efficiently because they are less prone to interference from the toxic byproducts of IR and desiccation [12], [17], [23].

More generally, our findings place constraints on the degree to which functional inferences can be made from whole-genome transcriptome analyses based on a single organism. For example, two independent analyses of gene induction in *D. radiodurans* recovering from different IR doses revealed numerous genes that are upregulated during the post-irradiation recovery, many of which were viewed as plausible candidates for a significant role in resistance [46], [92]. The hierarchy of induced genes in both transcriptome analyses was very similar, however, most of the highly induced *D. radiodurans* genes have no orthologs in *D. geothermalis*, and knockout of many of the uncharacterized unique *D. radiodurans* genes that were strongly induced by IR had little effect on IR resistance. A similar paradigm is emerging from the analysis of other systems, where the cellular transcriptional response to stress was largely stochastic, frequently involving genes known to be unrelated to the mechanisms under investigation [145]-[147]. Thus, it stands to reason that any comprehensive bioinformatics effort aimed at deciphering a complex, multi-gene phenotype using whole-genome, transcriptome and proteome approaches should aim to study at least two closely-related species. In the present context of understanding the genomic basis of extreme resistance phenotypes and the nature of the common ancestor of the *Deinococcus*-*Thermus* group, we consider *Truepera radiovictrix* an appropriate next candidate for whole-genome sequencing. *T. radiovictrix* is a recently discovered, deeply branching representative of the *Deinococcus* branch that is both thermophilic and extremely IR-resistant [148].

## Materials and Methods

### Strains

The strains used were as follows: *Deinococcus radiodurans* (ATCC BAA-816), *Deinococcus geothermalis* (DSM 11300), and *Escherichia coli* (K-12) (MG1655).

### Cell Growth, Irradiation, Mutant Construction, and PCR

*D. radiodurans* strain ATCC BAA-816 was grown at 32°C in undefined liquid nutrient-rich medium TGY (1% tryptone/0.1% glucose/0.5% yeast extract) or on TGY solid medium [17]. In liquid culture, cell density was determined at 600 nm by a Beckman Coulter spectrophotometer. For acute IR (^60^Co Gammacell irradiation unit, J. L. Shepard and Associates, Model 109) or UV (254 nm) exposures, late logarithmic-phase *D. radiodurans* cultures [OD_600_ = 0.9, 1×10^8^ colony-forming units (cfu)/ml] were irradiated to the indicated doses (Figure 1). Cell viability and cell numbers were determined by plate assay as described previously [17]. Three independent cell cultures and irradiation treatments of the same kind were performed and served as biological replicates for determining irradiation resistance profiles. To test the predicted involvement of the indicated genes, a mutant (Figure S6) was generated using previously developed *D. radiodurans* disruption protocols [75]. PCR was carried out as described previously [46].

### Whole-Genome Sequencing, Assembly and Structural Analysis

The complete genome of *D. geothermalis* (DSM 11300) was sequenced at the Joint Genome Institute (JGI) using a combination of 3 kb-, 8 kb- and fosmid- (40 kb) libraries. Library construction, sequencing, finishing, and automated annotation steps were carried out as follows.

#### DNA shearing and sub-cloning

Approximately 3–5 µg of isolated DNA was randomly sheared to 3 kb fragments in a 100 µl volume using a HydroShear™ (Genomic Solutions, Ann Arbor, MI). The sheared DNA was immediately blunt end-repaired at room temperature for 40 min using 6 U of T4 DNA Polymerase (Roche Diagnostics, Indianapolis, IN), 30 U of DNA Polymerase I Klenow Fragment (NEB, Beverly, MA), 10 µl of 10 mM dNTP mix (GE Healthcare, Piscataway, NJ), and 13 µl of 10× Klenow Buffer in a 130 µl total volume. After incubation, the reaction was heat-inactivated for 15 min at 70°C, cooled to 4°C for 10 min, and then frozen at −20°C for storage. The end-repaired DNA was run on a 1% Tris/Borate/EDTA (TBE) agarose gel for ∼60 min at 120 volts. Using ethidium bromide stain and UV illumination, 3 kb sheared fragments were extracted from the agarose gel and purified using QIAquick™ Gel Extraction Kit (QIAGEN, Valencia, CA). Approximately 300 ng of purified fragment was blunt-end-ligated overnight at 16°C into the *Sma* I site of 100 ng of pUC18 cloning vector (Roche) using 12 U T4 DNA Ligase, 3.2 µl 10× buffer (Roche), and 4.8 µl 30% PEG in a 32 µl total reaction volume. A very similar process was carried out to create an 8 kb library in pMCL200 with 10 µg of isolated genomic DNA.

Following standard protocols, 1 µl of each ligation product (3 kb or 8 kb) was electroporated into DH10B Electromax™ cells (Invitrogen, Carlsbad, CA) using the GENE PULSER® II electroporator (Bio-Rad, Hercules, CA). Transformed cells were transferred into 1 mL of SOC medium and incubated at 37°C in a rotating wheel for 1 h. Cells (usually 20–50 µl) were spread on 22×22 cm LB agar plates containing 100 µg/mL of ampicillin (pUC19) or 20 µg/mL of chloramphenicol (pMCL200), 120 µg/mL of IPTG, and 50 µg/mL of X-GAL. Colonies were grown for 16 h at 37°C. Individual white recombinant colonies were selected and picked into 384-well microtiter plates containing LB/glycerol (7.5% v/v) media containing 50 µg/mL of ampicillin or 20 µg/mL of chloramphenicol using the Q-Bot™ multitasking robot (Genetix, Dorset, U.K.). To test the quality of the library, 48 colonies were directly PCR-amplified with pUC m13–28 and –40 primers using standard protocols. Libraries passed PCR quality control if they had >90% 3 kb inserts or 8 kb inserts, respectively. For more details, see research protocols at www.jgi.doe.gov.

#### Plasmid amplification

One µl-aliquots of saturated *E. coli* cultures (DH10B) containing (pUC19 vector with random 3 kb DNA inserts or pMCL200 vector with random 8 kb DNA inserts) were added to 5 µl of a 10 mM Tris-HCl pH 8.2 and 0.1 mM EDTA denaturation buffer. The mixtures were heat-lysed at 95°C for 5 min then placed at 4°C for 5 min. To these denatured products, 4 µl of a rolling circle amplification (RCA) reaction mixture (Templiphi™ DNA Sequencing Template Amplification Kit, GE Healthcare) were added. The amplification reactions were carried out at 30°C for 12–18 h. The amplified products were heat-inactivated at 65°C for 10 min then placed at 4°C until used as template for sequencing [149].

#### Plasmid sequencing

Aliquots of the 10 µl amplified plasmid RCA products were sequenced with standard pUC m13–28 or –40 primers. The reactions typically contained 1 µl of the RCA product, sequenced with 4 pmoles (1 µl) of standard M13–28 or –40 primers, 0.5 µl 5×buffer, 1.75 µl H_2_O, and 0.75 µl BigDye sequencing kit (Applied Biosystems) at 1 min denaturation and 25 cycles of 95°C for 30 sec, 50°C for 20 sec, 60°C for 4 min, and finally held at 4°C. The reactions were then purified by a magnetic bead protocol (see research protocols, www.jgi.doe.gov) and run on an ABI PRISM 3730xl (Applied Biosystems) capillary DNA sequencer.

#### Fosmid Library Construction

Approximately 15–20 µg of isolated DNA was randomly sheared to 40 kb fragments (25 cycles at speed code 17 using the large assembly, part # JHSH204007) in a 60 µL volume using a HydroShear™ (GeneMachines, San Carlos, CA). The sheared DNA was immediately blunt end-repaired at room temperature for 45 min using the End-It end-repair kit (Epicentre, Madison, WI). The end-repair reaction contained 60 µL sheared DNA, 8 µL of 10×End-It buffer, 8 µL of 2.5 mM End-It dNTP mix, 8 µL of 10 mM End-It ATP, and 4 µL of End-It Enzyme mix in a 80 µL total volume. After 45 min of incubation, the reaction was heat-inactivated for 10 min at 70°C, cooled to 4°C for 10 min and then frozen at −20°C for storage. The end-repaired DNA was run on a 1% TBE low melting point agarose gel for 13 hours using the following conditions (Temperature: 14°C, Voltage: 4.5 V/cm, Pulse initial: 1.0–final: 7.0 sec, Angle: 120°) on a BioRad Chef-DR III™ System PFGE system. Using standard procedures, the gel was stained with ethidium bromide, destained, and visualized under UV for less than 10 seconds while the 40 kb band was excised. DNA was extracted from the agarose gel and blunt-end ligated into pCC1FOS following the Copy Control Fosmid Kit (Epicentre) protocol. With minimal modifications to the Copy Control Fosmid Kit (Epicentre) protocol, the ligated DNA was packaged, infected and plated for picking and end-sequencing. For detailed JGI protocols used, please see research protocols at www.jgi.doe.gov.

#### Assembly and Structural Analysis

Draft assemblies were based on 34,919 total reads. The Phred/Phrap/Consed software package (http://www.phrap.com) was used for sequence assembly and quality assessment [150], [151]. After the whole-genome shotgun stage, sequence reads were assembled with parallel Phrap (High Performance Software, LLC). All mis-assemblies were corrected by editing in Consed [152], and gaps between contigs were closed by custom primer walk or PCR amplification (Roche Applied Science, Indianapolis, IN). The completed genome sequence of *D. geothermalis* (DSM 11300) contained 36,718 reads, achieving an average of 8-fold sequence coverage per base with an error rate less than 1 in 100,000. The *D. geothermalis* genome sequence can be accessed at GenBank, or at the JGI Integrated Microbial Genomes website (http://img.jgi.doe.gov). Predicted coding sequences were manually analyzed and evaluated using an Integrated Microbial Genomes (IMG) annotation pipeline (http://img.jgi.doe.gov). The general structure of the predicted *D. geothermalis* genome was examined by PFGE as described previously for *D. radiodurans* [77], [78]. For structural analysis, *D. geothermalis* was exposed to 0.2 kGy, which introduces approximately 0.013 DSB/Gy per genome, and the cells were then embedded and lysed in agarose. For PFGE of genomic DNA subjected to restriction endonuclease analysis, non-irradiated *D. geothermalis* cells were used.

### Orthologous Clusters and Evolutionary Reconstructions

Reconstructed clusters of orthologous genes for the *Deinococcus* and *Thermus* genomes (tdCOGs) were constructed using a technique based on the standard COG approach [35], [36], [153]. First, a coarse-grained classification was obtained by assigning predicted genes to the NCBI Clusters of Orthologous Groups of proteins (COGs) using the COGNITOR method [35]. Then, the genes were organized into tight clusters, based on triangles of best hits [36]. Proteins belonging to the same cluster were aligned using the MUSCLE program [154]; alignments were converted into PSI-BLAST PSSMs [94]. Subsequent PSI-BLAST searches using these PSSMs against a database of *Deinococcus* and *Thermus* proteins were used to merge homologous clusters and previously unclustered proteins into tdCOGs. Cases when proteins assigned to different COGs were automatically clustered into one tdCOG were resolved by manual curation (either COG or tdCOG assignment was changed to remove the contradiction).

Evolutionary events in the history of the *Deinococcus-Thermus* group were reconstructed using an *ad hoc* parsimony approach [27], [155], [156]. Presence/absence data from COG-based reconstruction of the deep ancestor of *Cyanobacteria, Actinobacteria* and *Deinococcus*-*Thermus* group [27] were added to the tdCOG phyletic patterns. Simple parsimony rules were used to infer the ancestral states and the evolutionary events in the history of the *Deinococcus* and *Thermus* genomes (e.g. a gene present in both *Deinococci* and in the deep ancestor but absent in both *Thermus* species was considered to be present in the *Deinococcus*-*Thermus* group ancestor and in the *Deinococcus* genus ancestor, but lost by the *Thermus* genus ancestor). The only departure from the straightforward parsimony inference was made for homologous tdCOGs that form clade-specific expanded families, e.g. there are several tdCOGs, all assigned into the same ancestral COG, with genes present in both *Deinococci* but in neither of the *Thermus* species. In this case, contrary to the formal parsimony assumption of multiple losses in the *Thermus* ancestor, the scenario was interpreted as multiple gains (due to duplications) in the *Deinococcus* ancestor (Table S10).

### X-Ray Fluorescence

XRF microscopy measurements were made at beamline 2ID-D at the APS as described previously [17]. Briefly, the 2ID-D is an undulator beamline with Fresnel zone plates focusing optics that produced a focal spot with a FWHM (full width at half maximum) spatial resolution of approximately 120 nm for these experiments. For each pixel, the full XRF spectrum between approximately 2 keV and 10 keV was measured using a silicon drift detector. Thus, the distribution of elements between phosphorus and zinc on the periodic table of elements could be measured with 120-nm resolution throughout a cell and its periphery (Figure 7). XRF microprobe measurements were made on *D. geothermalis* cells grown in TGY to OD_600_ 0.3 at 50°C; and *D. radiodurans* cells were grown in TGY to OD_600_ 0.3 at 32°C. The cells were deposited on grids as suspensions in TGY liquid medium, which served to help maintain the structure and viability of the cells as they dried.

## Supporting Information

Figure S1 Proposed evolutionary history of genome partitions in the Deinococcus-Thermus group.
(0.08 MB DOC)

Figure S2 Genome dot plots for homologous genome partitions of D. radiodurans and D. geothermalis.
(0.06 MB DOC)

Figure S3 Guanine quadruplet repeats in D. radiodurans.
(0.03 MB DOC)

Figure S4 Verification of the presence of megaplasmid DG206 in D. geothermalis (DSM11300).
(0.12 MB DOC)

Figure S5 Phylogenetic relationships of tdCOGs of the calcineurin-like phosphoesterase subfamily of COG0639 with proteins from other organisms represented by this COG.
(0.06 MB DOC)

Figure S6 Structure of D. radiodurans homozygous mutants.
(0.25 MB DOC)

Figure S7 The ESDSA model does not fully explain the early formation of covalently closed circular (ccc) derivatives of tandem duplications in irradiated D. radiodurans.
(0.08 MB DOC)

Figure S8 Multiple alignment comparisons for RecA proteins of the Thermus-Deinococcus group with selected representatives of other bacteria.
(0.05 MB DOC)

Figure S9 Chrome azurol S agar plate assay for siderophore production.
(0.13 MB DOC)

Figure S10 Whereas the nramp gene of D. radiodurans is essential, the fur gene is dispensable.
(0.13 MB DOC)

Table S1 Homology between the D. radiodurans and D. geothermalis megaplasmids.
(0.04 MB DOC)

Table S2 Clusters of orthologous groups of proteins for Deinococcus and Thermus (tdCOGs).
(0.24 MB TXT)

Table S3 Lineage specific expansion of selected families in D. geothermalis (DG), D. radiodurans (DR), T. thermophilus HB27 (TT27), and T. thermophilus HB8 (TT8).
(0.05 MB DOC)

Table S4 Protein families expanded in D. geothermalis.
(0.05 MB DOC)

Table S5 Protein families expanded in D. radiodurans.
(0.07 MB DOC)

Table S6 Gene context and motifs of predicted cytoplasmic proteins shared by two Deinococcus species, but for which homologs outside the lineage do not exist.
(0.17 MB DOC)

Table S7 Stress response-related genes in D. radiodurans (DR), D. geothermalis (DG) and T. thermophilus (TT).
(0.23 MB DOC)

Table S8 Genes coding for replication, repair and recombination functions in E. coli, D. radiodurans and T. thermophilus.
(0.15 MB DOC)

Table S9 Manganese- and iron-related homeostasis genes.
(0.08 MB DOC)

Table S10 Parsimony pattern rules for reconstruction of evolutionary events in the Deinococcus/Thermus lineage.
(0.11 MB DOC)

## References

[1] Gupta RS (1998). Protein phylogenies and signature sequences: A reappraisal of evolutionary relationships among archaebacteria, eubacteria, and eukaryotes.. Microbiol Mol Biol Rev.

[2] Wolf YI, Rogozin IB, Grishin NV, Tatusov RL, Koonin EV (2001). Genome trees constructed using five different approaches suggest new major bacterial clades.. BMC Evol Biol.

[3] Lai WA, Kampfer P, Arun AB, Shen FT, Huber B (2006). *Deinococcus ficus* sp. nov., isolated from the rhizosphere of *Ficus religiosa* L.. Int J Syst Evol Microbiol.

[4] Gutman PD, Fuchs P, Minton KW (1994). Restoration of the DNA damage resistance of *Deinococcus radiodurans* DNA polymerase mutants by *Escherichia coli* DNA polymerase I and Klenow fragment.. Mutat Res.

[5] Daly MJ, Gaidamakova EK, Matrosova VY, Vasilenko A, Zhai M (2004). Accumulation of Mn(II) in *Deinococcus radiodurans* facilitates gamma-radiation resistance.. Science.

[6] Daly MJ (2000). Engineering radiation-resistant bacteria for environmental biotechnology.. Curr Opin Biotechnol.

[7] Ferreira AC, Nobre MF, Rainey FA, Silva MT, Wait R (1997). *Deinococcus geothermalis* sp. nov. and *Deinococcus murrayi* sp. nov., two extremely radiation-resistant and slightly thermophilic species from hot springs.. Int J Syst Bacteriol.

[8] Saarimaa C, Peltola M, Raulio M, Neu TR, Salkinoja-Salonen MS (2006). Characterization of adhesion threads of *Deinococcus geothermalis* as type IV pili.. J Bacteriol.

[9] Kimura H, Asada R, Masta A, Naganuma T (2003). Distribution of microorganisms in the subsurface of the manus basin hydrothermal vent field in Papua New Guinea.. Appl Environ Microbiol.

[10] Marteinsson VT, Hauksdottir S, Hobel CF, Kristmannsdottir H, Hreggvidsson GO (2001). Phylogenetic diversity analysis of subterranean hot springs in Iceland.. Appl Environ Microbiol.

[11] Brim H, Venkateswaran A, Kostandarithes HM, Fredrickson JK, Daly MJ (2003). Engineering *Deinococcus geothermalis* for bioremediation of high-temperature radioactive waste environments.. Appl Environ Microbiol.

[12] Ghosal D, Omelchenko MV, Gaidamakova EK, Matrosova VY, Vasilenko A (2005). How radiation kills cells: survival of *Deinococcus radiodurans* and *Shewanella oneidensis* under oxidative stress.. FEMS Microbiol Rev.

[13] Fredrickson JK, Kostandarithes HM, Li SW, Plymale AE, Daly MJ (2000). Reduction of Fe(III), Cr(VI), U(VI), and Tc(VII) by *Deinococcus radiodurans* R1.. Appl Environ Microbiol.

[14] Brim H, McFarlan SC, Fredrickson JK, Minton KW, Zhai M (2000). Engineering *Deinococcus radiodurans* for metal remediation in radioactive mixed waste environments.. Nat Biotechnol.

[15] Brim H, Osborne JP, Kostandarithes HM, Fredrickson JK, Wackett LP (2006). *Deinococcus radiodurans* engineered for complete toluene degradation facilitates Cr(VI) reduction.. Microbiology.

[16] White O, Eisen JA, Heidelberg JF, Hickey EK, Peterson JD (1999). Genome Sequence of the Radioresistant Bacterium *Deinococcus radiodurans* R1.. Science.

[17] Daly MJ, Gaidamakova EK, Matrosova VY, Vasilenko A, Zhai M (2007). Protein Oxidation Implicated as the Primary Determinant of Bacterial Radioresistance.. PLoS Biol.

[18] Daly MJ, Ouyang L, Fuchs P, Minton KW (1994). *In vivo* damage and *recA*-dependent repair of plasmid and chromosomal DNA in the radiation-resistant bacterium *Deinococcus radiodurans*.. J Bacteriol.

[19] Gerard E, Jolivet E, Prieur D, Forterre P (2001). DNA protection mechanisms are not involved in the radioresistance of the hyperthermophilic archaea *Pyrococcus abyssi* and *P. furiosus*.. Mol Genet Genomics.

[20] Cox MM, Battista JR (2005). *Deinococcus radiodurans*-the consummate survivor.. Nat Rev Microbiol.

[21] Makarova KS, Aravind L, Wolf YI, Tatusov RL, Minton KW (2001). Genome of the extremely radiation-resistant bacterium *Deinococcus radiodurans* viewed from the perspective of comparative genomics.. Microbiol Mol Biol Rev.

[22] Zahradka K, Slade D, Bailone A, Sommer S, Averbeck D (2006). Reassembly of shattered chromosomes in *Deinococcus radiodurans*.. Nature.

[23] Daly MJ (2006). Modulating radiation resistance: Insights based on defenses against reactive oxygen species in the radioresistant bacterium *Deinococcus radiodurans*.. Clin Lab Med.

[24] Zimmerman JM, Battista JR (2005). A ring-like nucleoid is not necessary for radioresistance in the *Deinococcaceae*.. BMC Microbiol.

[25] Pavlov AK, Kalinin VL, Konstantinov AN, Shelegedin VN, Pavlov AA (2006). Was Earth ever infected by martian biota? Clues from radioresistant bacteria.. Astrobiology.

[26] Lin J, Qi R, Aston C, Jing J, Anantharaman TS (1999). Whole-genome shotgun optical mapping of *Deinococcus radiodurans*.. Science.

[27] Omelchenko MV, Wolf YI, Gaidamakova EK, Matrosova VY, Vasilenko A (2005). Comparative genomics of *Thermus thermophilus* and *Deinococcus radiodurans*: divergent routes of adaptation to thermophily and radiation resistance.. BMC Evol Biol.

[28] Eisen JA, Heidelberg JF, White O, Salzberg SL (2000). Evidence for symmetric chromosomal inversions around the replication origin in bacteria.. Genome Biol.

[29] Makarova KS, Wolf YI, White O, Minton K, Daly MJ (1999). Short repeats and IS elements in the extremely radiation-resistant bacterium *Deinococcus radiodurans* and comparison to other bacterial species.. Res Microbiol.

[30] Makarova KS, Aravind L, Galperin MY, Grishin NV, Tatusov RL (1999). Comparative genomics of the Archaea (Euryarchaeota): evolution of conserved protein families, the stable core, and the variable shell.. Genome Res.

[31] Sen D, Gilbert W (1988). Formation of parallel four-stranded complexes by guanine-rich motifs in DNA and its implications for meiosis.. Nature.

[32] Mojica FJ, Diez-Villasenor C, Soria E, Juez G (2000). Biological significance of a family of regularly spaced repeats in the genomes of Archaea, Bacteria and mitochondria.. Mol Microbiol.

[33] Barrangou R, Fremaux C, Deveau H, Richards M, Boyaval P (2007). CRISPR provides acquired resistance against viruses in prokaryotes.. Science.

[34] Makarova KS, Grishin NV, Shabalina SA, Wolf YI, Koonin EV (2006). A putative RNA-interference-based immune system in prokaryotes: computational analysis of the predicted enzymatic machinery, functional analogies with eukaryotic RNAi, and hypothetical mechanisms of action.. Biol Direct.

[35] Tatusov RL, Fedorova ND, Jackson JD, Jacobs AR, Kiryutin B (2003). The COG database: an updated version includes eukaryotes.. BMC Bioinformatics.

[36] Tatusov RL, Koonin EV, Lipman DJ (1997). A genomic perspective on protein families.. Science.

[37] Yokoyama K, Ishijima SA, Clowney L, Koike H, Aramaki H (2006). Feast/famine regulatory proteins (FFRPs): *Escherichia coli* Lrp, AsnC and related archaeal transcription factors.. FEMS Microbiol Rev.

[38] Gerischer U (2002). Specific and global regulation of genes associated with the degradation of aromatic compounds in bacteria.. J Mol Microbiol Biotechnol.

[39] Molina-Henares AJ, Krell T, Eugenia Guazzaroni M, Segura A, Ramos JL (2006). Members of the IclR family of bacterial transcriptional regulators function as activators and/or repressors.. FEMS Microbiol Rev.

[40] Ramos JL, Martinez-Bueno M, Molina-Henares AJ, Teran W, Watanabe K (2005). The TetR family of transcriptional repressors.. Microbiol Mol Biol Rev.

[41] Hobman JL, Wilkie J, Brown NL (2005). A design for life: prokaryotic metal-binding MerR family regulators.. Biometals.

[42] Bearson SM, Albrecht JA, Gunsalus RP (2002). Oxygen and nitrate-dependent regulation of *dmsABC* operon expression in *Escherichia coli*: sites for Fnr and NarL protein interactions.. BMC Microbiol.

[43] Sandrini MP, Clausen AR, Munch-Petersen B, Piskur J (2006). Thymidine kinase diversity in bacteria.. Nucleosides Nucleotides Nucleic Acids.

[44] Xi H, Schneider BL, Reitzer L (2000). Purine catabolism in *Escherichia coli* and function of xanthine dehydrogenase in purine salvage.. J Bacteriol.

[45] Knofel T, Strater N (1999). X-ray structure of the *Escherichia coli* periplasmic 5′-nucleotidase containing a dimetal catalytic site.. Nat Struct Biol.

[46] Liu Y, Zhou J, Omelchenko MV, Beliaev AS, Venkateswaran A (2003). Transcriptome dynamics of *Deinococcus radiodurans* recovering from ionizing radiation.. Proc Natl Acad Sci U S A.

[47] Galperin MY, Moroz OV, Wilson KS, Murzin AG (2006). House cleaning, a part of good housekeeping.. Mol Microbiol.

[48] Shallom D, Shoham Y (2003). Microbial hemicellulases.. Curr Opin Microbiol.

[49] Kolari M, Nuutinen J, Rainey FA, Salkinoja-Salonen MS (2003). Colored moderately thermophilic bacteria in paper-machine biofilms.. J Ind Microbiol Biotechnol.

[50] Vaisanen OM, Weber A, Bennasar A, Rainey FA, Busse HJ (1998). Microbial communities of printing paper machines.. J Appl Microbiol.

[51] Venkateswaran A, McFarlan SC, Ghostal D, Minton KW, Vasilenko A (2000). Physiologic determinants of radiation resistance in *Deinococcus radiodurans*.. ApplEnvironMicrobiol.

[52] Holland AD, Rothfuss HM, Lidstrom ME (2006). Development of a defined medium supporting rapid growth for *Deinococcus radiodurans* and analysis of metabolic capacities.. Appl Microbiol Biotechnol.

[53] Frankenberg N, Moser J, Jahn D (2003). Bacterial heme biosynthesis and its biotechnological application.. Appl Microbiol Biotechnol.

[54] Pereira MM, Santana M, Teixeira M (2001). A novel scenario for the evolution of haem-copper oxygen reductases.. Biochim Biophys Acta.

[55] Junemann S (1997). Cytochrome bd terminal oxidase.. Biochim Biophys Acta.

[56] Harborne NR, Griffiths L, Busby SJ, Cole JA (1992). Transcriptional control, translation and function of the products of the five open reading frames of the *Escherichia coli nir* operon.. Mol Microbiol.

[57] Cabello P, Pino C, Olmo-Mira MF, Castillo F, Roldan MD (2004). Hydroxylamine assimilation by *Rhodobacter capsulatus* E1F1. requirement of the *hcp* gene (hybrid cluster protein) located in the nitrate assimilation *nas* gene region for hydroxylamine reduction.. J Biol Chem.

[58] Zumft WG (1997). Cell biology and molecular basis of denitrification.. Microbiol Mol Biol Rev.

[59] Nakamura K, Kawabata T, Yura K, Go N (2003). Novel types of two-domain multi-copper oxidases: possible missing links in the evolution.. FEBS Lett.

[60] Hille R (2002). Molybdenum and tungsten in biology.. Trends Biochem Sci.

[61] Makarova KS, Aravind L, Daly MJ, Koonin EV (2000). Specific expansion of protein families in the radioresistant bacterium *Deinococcus radiodurans*.. Genetica.

[62] Fisher DI, Cartwright JL, McLennan AG (2006). Characterization of the Mn(2+)-stimulated (di)adenosine polyphosphate hydrolase encoded by the *Deinococcus radiodurans* DR2356 nudix gene.. Arch Microbiol.

[63] Hou B, Xu ZW, Yang CW, Gao Y, Zhao SF (2007). Protective effects of inosine on mice subjected to lethal total-body ionizing irradiation.. J Radiat Res (Tokyo).

[64] Azeddoug H, Reysset G (1994). Cloning and sequencing of a chromosomal fragment from *Clostridium acetobutylicum* strain ABKn8 conferring chemical-damaging agents and UV resistance to *E. coli recA* strains.. Curr Microbiol.

[65] Jobling MG, Ritchie DA (1988). The nucleotide sequence of a plasmid determinant for resistance to tellurium anions.. Gene.

[66] Kitajima S, Sato F (1999). Plant pathogenesis-related proteins: molecular mechanisms of gene expression and protein function.. J Biochem (Tokyo).

[67] Miura S, Zou W, Ueda M, Tanaka A (2000). Screening of genes involved in isooctane tolerance in *Saccharomyces cerevisiae* by using mRNA differential display.. Appl Environ Microbiol.

[68] Brinkrolf K, Brune I, Tauch A (2006). Transcriptional regulation of catabolic pathways for aromatic compounds in *Corynebacterium glutamicum*.. Genet Mol Res.

[69] Gury J, Barthelmebs L, Tran NP, Divies C, Cavin JF (2004). Cloning, deletion, and characterization of PadR, the transcriptional repressor of the phenolic acid decarboxylase-encoding *padA* gene of *Lactobacillus plantarum*.. Appl Environ Microbiol.

[70] Huillet E, Velge P, Vallaeys T, Pardon P (2006). LadR, a new PadR-related transcriptional regulator from *Listeria monocytogenes*, negatively regulates the expression of the multidrug efflux pump MdrL.. FEMS Microbiol Lett.

[71] Kobayashi I, Tamura T, Sghaier H, Narumi I, Yamaguchi S (2006). Characterization of monofunctional catalase KatA from radioresistant bacterium *Deinococcus radiodurans*.. J Biosci Bioeng.

[72] Yun EJ, Lee YN (2000). Production of two different catalase-peroxidases by *Deinococcus radiophilus*.. FEMS Microbiol Lett.

[73] Imlay JA (2003). Pathways of oxidative damage.. Annu Rev Microbiol.

[74] Nurizzo D, Shewry SC, Perlin MH, Brown SA, Dholakia JN (2003). The crystal structure of aminoglycoside-3′-phosphotransferase-IIa, an enzyme responsible for antibiotic resistance.. J Mol Biol.

[75] Markillie LM, Varnum SM, Hradecky P, Wong KK (1999). Targeted mutagenesis by duplication insertion in the radioresistant bacterium *Deinococcus radiodurans*: radiation sensitivities of catalase (*katA*) and superoxide dismutase (*sodA*) mutants.. J Bacteriol.

[76] Daly MJ, Ling O, Minton KW (1994). Interplasmidic recombination following irradiation of the radioresistant bacterium *Deinococcus radiodurans*.. J Bacteriol.

[77] Daly MJ, Minton KW (1995). Interchromosomal recombination in the extremely radioresistant bacterium *Deinococcus radiodurans*.. J Bacteriol.

[78] Daly MJ, Minton KW (1996). An alternative pathway of recombination of chromosomal fragments precedes *recA*-dependent recombination in the radioresistant bacterium *Deinococcus radiodurans*.. J Bacteriol.

[79] Daly MJ, Minton KW (1997). Recombination between a resident plasmid and the chromosome following irradiation of the radioresistant bacterium *Deinococcus radiodurans*.. Gene.

[80] Gallegos MT, Schleif R, Bairoch A, Hofmann K, Ramos JL (1997). Arac/XylS family of transcriptional regulators.. Microbiol Mol Biol Rev.

[81] Sorensen KI, Hove-Jensen B (1996). Ribose catabolism of *Escherichia coli*: characterization of the *rpiB* gene encoding ribose phosphate isomerase B and of the *rpiR* gene, which is involved in regulation of *rpiB* expression.. J Bacteriol.

[82] Labie C, Bouche F, Bouche JP (1989). Isolation and mapping of *Escherichia coli* mutations conferring resistance to division inhibition protein DicB.. J Bacteriol.

[83] Pennella MA, Giedroc DP (2005). Structural determinants of metal selectivity in prokaryotic metal-responsive transcriptional regulators.. Biometals.

[84] Buts L, Lah J, Dao-Thi MH, Wyns L, Loris R (2005). Toxin-antitoxin modules as bacterial metabolic stress managers.. Trends Biochem Sci.

[85] Gerdes K, Christensen SK, Lobner-Olesen A (2005). Prokaryotic toxin-antitoxin stress response loci.. Nat Rev Microbiol.

[86] Bhoo SH, Davis SJ, Walker J, Karniol B, Vierstra RD (2001). Bacteriophytochromes are photochromic histidine kinases using a biliverdin chromophore.. Nature.

[87] Davis SJ, Vener AV, Vierstra RD (1999). Bacteriophytochromes: phytochrome-like photoreceptors from nonphotosynthetic eubacteria.. Science.

[88] Boyd A, Chakrabarty AM (1995). *Pseudomonas aeruginosa* biofilms: role of the alginate exopolysaccharide.. J Ind Microbiol.

[89] Gacesa P (1998). Bacterial alginate biosynthesis–recent progress and future prospects.. Microbiology.

[90] Agostini HJ, Carroll JD, Minton KW (1996). Identification and characterization of *uvrA*, a DNA repair gene of *Deinococcus radiodurans*.. J Bacteriol.

[91] Lipton MS, Pasa-Tolic L, Anderson GA, Anderson DJ, Auberry DL (2002). Global analysis of the *Deinococcus radiodurans* proteome by using accurate mass tags.. Proc Natl Acad Sci U S A.

[92] Tanaka M, Earl AM, Howell HA, Park MJ, Eisen JA (2004). Analysis of *Deinococcus radiodurans*'s transcriptional response to ionizing radiation and desiccation reveals novel proteins that contribute to extreme radioresistance.. Genetics.

[93] Gutman PD, Carroll JD, Masters CI, Minton KW (1994). Sequencing, targeted mutagenesis and expression of a *recA* gene required for the extreme radioresistance of *Deinococcus radiodurans*.. Gene.

[94] Altschul SF, Koonin EV (1998). Iterated profile searches with PSI-BLAST–a tool for discovery in protein databases.. Trends Biochem Sci.

[95] Schultz J, Copley RR, Doerks T, Ponting CP, Bork P (2000). SMART: a web-based tool for the study of genetically mobile domains.. Nucleic Acids Res.

[96] Marchler-Bauer A, Panchenko AR, Shoemaker BA, Thiessen PA, Geer LY (2002). CDD: a database of conserved domain alignments with links to domain three-dimensional structure.. Nucleic Acids Res.

[97] Iyer LM, Koonin EV, Aravind L (2002). Classification and evolutionary history of the single-strand annealing proteins, RecT, Redbeta, ERF and RAD52.. BMC Genomics.

[98] Harris DR, Tanaka M, Saveliev SV, Jolivet E, Earl AM (2004). Preserving genome integrity: the DdrA protein of *Deinococcus radiodurans* R1.. PLoS Biol.

[99] Mironov AA, Koonin EV, Roytberg MA, Gelfand MS (1999). Computer analysis of transcription regulatory patterns in completely sequenced bacterial genomes.. Nucleic Acids Res.

[100] Mironov AA, Vinokurova NP, Gel'fand MS (2000). [Software for analyzing bacterial genomes].. Mol Biol (Mosk).

[101] Kunkel TA, Erie DA (2005). DNA mismatch repair.. Annu Rev Biochem.

[102] Kuzminov A (1999). Recombinational repair of DNA damage in *Escherichia coli* and bacteriophage lambda.. Microbiol Mol Biol Rev.

[103] Touati E, Laurent-Winter C, Quillardet P, Hofnung M (1996). Global response of *Escherichia coli* cells to a treatment with 7-methoxy-2-nitronaphtho[2,1-b]furan (R7000), an extremely potent mutagen.. Mutat Res.

[104] Zhang YM, Liu JK, Wong TY (2003). The DNA excision repair system of the highly radioresistant bacterium *Deinococcus radiodurans* is facilitated by the pentose phosphate pathway.. Mol Microbiol.

[105] Chasin LA, Magasanik B (1968). Induction and repression of the histidine-degrading enzymes of *Bacillus subtilis*.. J Biol Chem.

[106] Kannan K, Janiyani KL, Shivaji S, Ray MK (1998). Histidine utilisation operon (*hut*) is upregulated at low temperature in the antarctic psychrotrophic bacterium *Pseudomonas syringae*.. FEMS Microbiol Lett.

[107] Bruce AK, Berner JD (1976). Respiratory activity as a determinant of radiation survival response.. Can J Microbiol.

[108] Earl AM, Mohundro MM, Mian IS, Battista JR (2002). The IrrE protein of *Deinococcus radiodurans* R1 is a novel regulator of *recA* expression.. J Bacteriol.

[109] Hua Y, Narumi I, Gao G, Tian B, Satoh K (2003). PprI: a general switch responsible for extreme radioresistance of *Deinococcus radiodurans*.. Biochem Biophys Res Commun.

[110] Gao G, Le D, Huang L, Lu H, Narumi I (2006). Internal promoter characterization and expression of the *Deinococcus radiodurans pprI-folP* gene cluster.. FEMS Microbiol Lett.

[111] Carroll JD, Daly MJ, Minton KW (1996). Expression of *recA* in *Deinococcus radiodurans*.. J Bacteriol.

[112] Cheo DL, Bayles KW, Yasbin RE (1991). Cloning and characterization of DNA damage-inducible promoter regions from *Bacillus subtilis*.. J Bacteriol.

[113] Yasbin RE, Cheo D, Bayles KW (1991). The SOB system of *Bacillus subtilis*: a global regulon involved in DNA repair and differentiation.. Res Microbiol.

[114] Courcelle J, Khodursky A, Peter B, Brown PO, Hanawalt PC (2001). Comparative gene expression profiles following UV exposure in wild-type and SOS-deficient *Escherichia coli*.. Genetics.

[115] Fernandez De Henestrosa AR, Ogi T, Aoyagi S, Chafin D, Hayes JJ (2000). Identification of additional genes belonging to the LexA regulon in *Escherichia coli*.. Mol Microbiol.

[116] Narumi I, Satoh K, Kikuchi M, Funayama T, Yanagisawa T (2001). The LexA protein from *Deinococcus radiodurans* is not involved in RecA induction following gamma irradiation.. J Bacteriol.

[117] Little JW, Mount DW, Yanisch-Perron CR (1981). Purified LexA protein is a repressor of the *recA* and *lexA* genes.. Proc Natl Acad Sci U S A.

[118] Moseley BE, Mattingly A (1971). Repair of irradiation transforming deoxyribonucleic acid in wild type and a radiation-sensitive mutant of *Micrococcus radiodurans*.. J Bacteriol.

[119] Gutman PD, Yao HL, Minton KW (1991). Partial complementation of the UV sensitivity of *Deinococcus radiodurans* excision repair mutants by the cloned *denV* gene of bacteriophage T4.. Mutat Res.

[120] Minton KW (1996). Repair of ionizing-radiation damage in the radiation resistant bacterium *Deinococcus radiodurans*.. Mutat Res.

[121] Schlesinger DJ (2007). Role of RecA in DNA damage repair in *Deinococcus radiodurans*.. FEMS Microbiol Lett.

[122] Daly MJ, Minton KW (1995). Resistance to radiation.. Science.

[123] Eltsov M, Dubochet J (2005). Fine structure of the *Deinococcus radiodurans* nucleoid revealed by cryoelectron microscopy of vitreous sections.. J Bacteriol.

[124] Eltsov M, Dubochet J (2006). Study of the *Deinococcus radiodurans* nucleoid by cryoelectron microscopy of vitreous sections: Supplementary comments.. J Bacteriol.

[125] Gao G, Lu H, Yin L, Hua Y (2007). Ring-like nucleoid does not play a key role in radioresistance of *Deinococcus radiodurans*.. Sci China C Life Sci.

[126] Setlow DM, Duggan DE (1964). The resistance of *Micrococcus radiodurans* to ultraviolet radiation: Ultraviolet-induced lesions in the cell's DNA.. Biochim Biophys Acta.

[127] Huang L, Hua X, Lu H, Gao G, Tian B (2007). Three tandem HRDC domains have synergistic effect on the RecQ functions in *Deinococcus radiodurans*.. DNA Repair (Amst).

[128] Kim JI, Sharma AK, Abbott SN, Wood EA, Dwyer DW (2002). RecA Protein from the extremely radioresistant bacterium *Deinococcus radiodurans*: expression, purification, and characterization.. J Bacteriol.

[129] Narumi I, Satoh K, Kikuchi M, Funayama T, Kitayama S (1999). Molecular analysis of the *Deinococcus radiodurans recA* locus and identification of a mutation site in a DNA repair-deficient mutant, rec30.. Mutat Res.

[130] Bernstein DA, Eggington JM, Killoran MP, Misic AM, Cox MM (2004). Crystal structure of the *Deinococcus radiodurans* single-stranded DNA-binding protein suggests a mechanism for coping with DNA damage.. Proc Natl Acad Sci U S A.

[131] Bowater R, Doherty AJ (2006). Making ends meet: repairing breaks in bacterial DNA by non-homologous end-joining.. PLoS Genet.

[132] Englander J, Klein E, Brumfeld V, Sharma AK, Doherty AJ (2004). DNA toroids: framework for DNA repair in *Deinococcus radiodurans* and in germinating bacterial spores.. J Bacteriol.

[133] Kobayashi Y, Narumi I, Satoh K, Funayama T, Kikuchi M (2004). Radiation response mechanisms of the extremely radioresistant bacterium *Deinococcus radiodurans*.. Biol Sci Space.

[134] Lecointe F, Shevelev IV, Bailone A, Sommer S, Hubscher U (2004). Involvement of an X family DNA polymerase in double-stranded break repair in the radioresistant organism *Deinococcus radiodurans*.. Mol Microbiol.

[135] Levin-Zaidman S, Englander J, Shimoni E, Sharma AK, Minton KW (2003). Ringlike structure of the *Deinococcus radiodurans* genome: a key to radioresistance?. Science.

[136] Narumi I, Satoh K, Cui S, Funayama T, Kitayama S (2004). PprA: a novel protein from *Deinococcus radiodurans* that stimulates DNA ligation.. Mol Microbiol.

[137] Blasius M, Buob R, Shevelev IV, Hubscher U (2007). Enzymes involved in DNA ligation and end-healing in the radioresistant bacterium *Deinococcus radiodurans*.. BMC Mol Biol.

[138] Wright EG, Coates PJ (2006). Untargeted effects of ionizing radiation: implications for radiation pathology.. Mutat Res.

[139] Belyakov OV, Mitchell SA, Parikh D, Randers-Pehrson G, Marino SA (2005). Biological effects in unirradiated human tissue induced by radiation damage up to 1 mm away.. Proc Natl Acad Sci U S A.

[140] von Sonntag C (1987). The chemical basis of radiation biology..

[141] Du J, Gebicki JM (2004). Proteins are major initial cell targets of hydroxyl free radicals.. Int J Biochem Cell Biol.

[142] Kemner KM, Kelly SD, Lai B, Maser J, O'Loughlin E J (2004). Elemental and redox analysis of single bacterial cells by x-ray microbeam analysis.. Science.

[143] Heinz K, Marx A (2007). Lesion bypass activity of DNA polymerase A from the extremely radioresistant organism *Deinococcus radiodurans*.. J Biol Chem.

[144] Qiu X, Daly MJ, Vasilenko A, Omelchenko MV, Gaidamakova EK (2006). Transcriptome analysis applied to survival of *Shewanella oneidensis* MR-1 exposed to ionizing radiation.. J Bacteriol.

[145] Fong SS, Joyce AR, Palsson BO (2005). Parallel adaptive evolution cultures of *Escherichia coli* lead to convergent growth phenotypes with different gene expression states.. Genome Res.

[146] Koonin EV (2007). Chance and necessity in cellular response to challenge.. Molec Syst Biol: in press.

[147] Stern S, Dror T, Stolovicki E, Brenner N, Braun E (2007). Genome-wide transcriptional plasticity underlies cellular adaptation to novel changes.. Molec Syst Biol: in press.

[148] Albuquerque L, Simoes C, Nobre MF, Pino NM, Battista JR (2005). *Truepera radiovictrix* gen. nov., sp. nov., a new radiation resistant species and the proposal of *Trueperaceae* fam. nov.. FEMS Microbiol Lett.

[149] Detter JC, Jett JM, Lucas SM, Dalin E, Arellano AR (2002). Isothermal strand-displacement amplification applications for high-throughput genomics.. Genomics.

[150] Ewing B, Green P (1998). Base-calling of automated sequencer traces using phred. II. Error probabilities.. Genome Res.

[151] Ewing B, Hillier L, Wendl MC, Green P (1998). Base-calling of automated sequencer traces using phred. I. Accuracy assessment.. Genome Res.

[152] Gordon D, Abajian C, Green P (1998). Consed: a graphical tool for sequence finishing.. Genome Res.

[153] Tatusov RL, Natale DA, Garkavtsev IV, Tatusova TA, Shankavaram UT (2001). The COG database: new developments in phylogenetic classification of proteins from complete genomes.. Nucleic Acids Res.

[154] Edgar RC (2004). MUSCLE: multiple sequence alignment with high accuracy and high throughput.. Nucleic Acids Res.

[155] Makarova K, Slesarev A, Wolf Y, Sorokin A, Mirkin B (2006). Comparative genomics of the lactic acid bacteria.. Proc Natl Acad Sci U S A.

[156] Makarova KS, Wolf YI, Mekhedov SL, Mirkin BG, Koonin EV (2005). Ancestral paralogs and pseudoparalogs and their role in the emergence of the eukaryotic cell.. Nucleic Acids Res.

[157] Bordo D, Djinovic K, Bolognesi M (1994). Conserved patterns in the Cu,Zn superoxide dismutase family.. J Mol Biol.

[158] Singleton MR, Wentzell LM, Liu Y, West SC, Wigley DB (2002). Structure of the single-strand annealing domain of human RAD52 protein.. Proc Natl Acad Sci U S A.

[159] Bouchard JD, Moineau S (2004). Lactococcal phage genes involved in sensitivity to AbiK and their relation to single-strand annealing proteins.. J Bacteriol.

[160] Schneider TD, Stephens RM (1990). Sequence logos: a new way to display consensus sequences.. Nucleic Acids Res.

[161] Kitayama S, Kohoroku M, Takagi A, Itoh H (1997). Mutation of *D. radiodurans* in a gene homologous to *ruvB* of *E. coli*.. Mutat Res.

[162] Udupa KS, O'Cain PA, Mattimore V, Battista JR (1994). Novel ionizing radiation-sensitive mutants of *Deinococcus radiodurans*.. J Bacteriol.

[163] Battista JR, Park MJ, McLemore AE (2001). Inactivation of Two Homologues of Proteins Presumed to be Involved in the Desiccation Tolerance of Plants Sensitizes *Deinococcus radiodurans* R1 to Desiccation.. Cryobiology.

[164] Kitayama S, Narumi I, Kikuchi M, Watanabe H (2000). Mutation in recR gene of *Deinococcus radiodurans* and possible involvement of its product in the repair of DNA interstrand cross-links.. Mutat Res.

[165] Funayama T, Narumi I, Kikuchi M, Kitayama S, Watanabe H (1999). Identification and disruption analysis of the recN gene in the extremely radioresistant bacterium *Deinococcus radiodurans*.. Mutat Res.

[166] Gutman PD, Fuchs P, Ouyang L, Minton KW (1993). Identification, sequencing, and targeted mutagenesis of a DNA polymerase gene required for the extreme radioresistance of *Deinococcus radiodurans*.. J Bacteriol.

[167] Mattimore V, Battista JR (1996). Radioresistance of *Deinococcus radiodurans*: functions necessary to survive ionizing radiation are also necessary to survive prolonged desiccation.. J Bacteriol.

[168] Satoh K, Ohba H, Sghaier H, Narumi I (2006). Down-regulation of radioresistance by LexA2 in *Deinococcus radiodurans*.. Microbiology.

[169] Sheng D, Zheng Z, Tian B, Shen B, Hua Y (2004). LexA analog (dra0074) is a regulatory protein that is irrelevant to recA induction.. J Biochem (Tokyo).

[170] Huang LF, Zhang SW, Hua XT, Gao GJ, Hua YJ (2006). [Construction of the *recQ* double mutants and analysis of adversity in *Deinococcus radiodurans*].. Wei Sheng Wu Xue Bao.

