# Supplementary material for: Deinococcus geothermalis: The Pool of Extreme Radiation Resistance Genes Shrinks
Source: PLoS One. 2007 Sep 26;2(9):e955. doi: 10.1371/journal.pone.0000955 (PMC1978522; doi:10.1371/journal.pone.0000955)
Supplement: Figure S1 — Proposed evolutionary history of genome partitions in the Deinococcus-Thermus group. (0.08 MB DOC) [file pone.0000955.s001.doc]

**Figure S1**

**Figure S1.** Proposed evolutionary history of genome partitions in the *Deinococcus-Thermus* group. Homologous partitions are shown in the same color. The dashed circle (green-cyan) denotes a putative plasmid present in the *Deinococcus* ancestor that gave rise to one or both of the smallest partitions in *D. radiodurans* and *D. geothermalis*. In *D. radiodurans*: black, chromosome; pink, DR412; red, DR177; green, 46 kb plasmid. In *D. geothermalis*: black, chromosome; pink-red, DG574; cyan, DG206; *T. thermophilus*: black, chromosome; red, pTT27. See also *Deinococcus* plasmid comparisons in Table S1.
