# Supplementary material for: Deinococcus geothermalis: The Pool of Extreme Radiation Resistance Genes Shrinks
Source: PLoS One. 2007 Sep 26;2(9):e955. doi: 10.1371/journal.pone.0000955 (PMC1978522; doi:10.1371/journal.pone.0000955)
Supplement: Figure S2 — Genome dot plots for homologous genome partitions of D. radiodurans and D. geothermalis. (0.06 MB DOC) [file pone.0000955.s002.doc]

**Figure S2**

**A**

**B**

**Figure S2.** Genome dot plots for homologous genome partitions of *D. radiodurans* (vertical axis) and *D. geothermalis* (horizontal axis). Each dot represents the location of a pair of symmetrical best hits between the two genomes. The coordinates correspond to the assigned gene-numbers in the respective partition. **A**, chromosomes. **B**, megaplasmids (DR412/DG574).
