# Supplementary material for: Deinococcus geothermalis: The Pool of Extreme Radiation Resistance Genes Shrinks
Source: PLoS One. 2007 Sep 26;2(9):e955. doi: 10.1371/journal.pone.0000955 (PMC1978522; doi:10.1371/journal.pone.0000955)
Supplement: Figure S3 — Guanine quadruplet repeats in D. radiodurans. (0.03 MB DOC) [file pone.0000955.s003.doc]

**Figure S3**

**cctct GGGGTAAGGGGCGGGGGGATGGGG ccaaa [ 169911 ]**

**cctct GGGGATAGGGGTTTGGGGTTGGGG ccaaa [ 445880 ]**

**cctct GGGGTAAGGGGCTGGGGGATGGGG caaaa [ 976013 ]**

**agtct GGGGTAAGGGGCTGGGGGATGGGG caaaa [ 1106621 ]**

**cctct GGGGTAAGGGGCTGGGGGATGGGG ccaaa [ 1112434 ]**

**agtct GGGGTAAGGGGCTGGGGGATGGGG caaaa [ 1159594 ]**

**cctgt GGGGATAGGGGTTTGGGGTTGGGG tcaaa [ 1267480 ]**

**ccttt GGGGTAAGGGGCTGGGGGATGGGG ccaaa [ 1904432 ]**

**cctct GGGGAGAGGGGTTTGGGGTTGGGG ccaaa [ 2199713 ]**

**cctct GGGGTAGGGGGTTTGGGGGCGGGG caaaa [ 2279681 ]**

**ccttt GGGGATAGGGGTTTGGGGTTGGGG ctaaa [ 1718617 ]**

**ccttt GGGGTAAGGGGCTGGGGGATGGGG ctaaa [ 1771700 ]**

**Figure S3.** Guanine quadruplet repeats in *D. radiodurans*. Coordinates are shown in brackets for the *D. radiodurans* chromosome.
