# Supplementary material for: Deinococcus geothermalis: The Pool of Extreme Radiation Resistance Genes Shrinks
Source: PLoS One. 2007 Sep 26;2(9):e955. doi: 10.1371/journal.pone.0000955 (PMC1978522; doi:10.1371/journal.pone.0000955)
Supplement: Figure S4 — Verification of the presence of megaplasmid DG206 in D. geothermalis (DSM11300). (0.12 MB DOC) [file pone.0000955.s004.doc]

**Figure S4**

**Figure S4.** Verification of the presence of megaplasmid DG206 in *D. geothermalis* (DSM11300). **A**, Predicted DNA band-sizes of PCR product after cleavage with restriction endonuclease *Ban*II. Primers for PCR reaction with DSM11300 total DNA were chosen from the DG206 sequence acquired at JGI. Forward primer (Fp): 5’AGCGGCGTTGCCACGTTCTCC3’. Reverse primer (Rp): 5’CCGTGTCCGCCGCACCCAC3’. **B**, 3% (w/v) agarose gel (NuSieve). Lanes: 1, Mass Ruler DNA Ladder Low Range (MBI Fermentas); 2, PCR product from a DNA sample used for genome sequencing at JGI; 3, PCR product shown in lane 2 digested with *Ban*II; 4, PCR product from an independent DNA preparation of DSM11300 prepared at USUHS; 5, PCR product shown in lane 4 digested with *Ban*II; 6, Gene Ruler 50 bp DNA Ladder (MBI Fermentas).
