# Supplementary material for: Deinococcus geothermalis: The Pool of Extreme Radiation Resistance Genes Shrinks
Source: PLoS One. 2007 Sep 26;2(9):e955. doi: 10.1371/journal.pone.0000955 (PMC1978522; doi:10.1371/journal.pone.0000955)
Supplement: Figure S5 — Phylogenetic relationships of tdCOGs of the calcineurin-like phosphoesterase subfamily of COG0639 with proteins from other organisms represented by this COG. (0.06 MB DOC) [file pone.0000955.s005.doc]

**Figure S5**


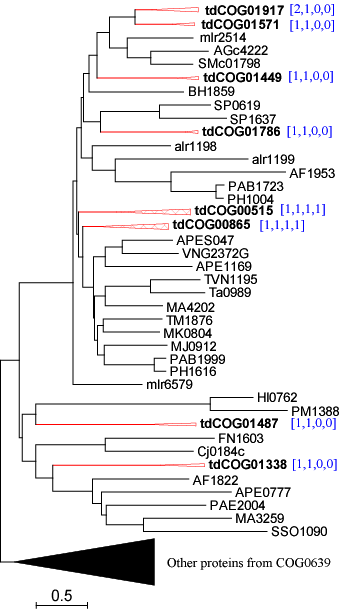


**Figure S5.** Phylogenetic relationships of tdCOGs of the calcineurin-like phosphoesterase subfamily of COG0639 with proteins from other organisms represented by this COG. All proteins of the corresponding tdCOGs were added to the proteins of COG0639 in other organisms and the multiple alignment was built using the MUSCLE program [S1].The neighbor-joining tree was reconstructed using the MEGA program [S2]. Each terminal node of the tree is labeled by the protein identifiers for other bacteria and archaea, or tdCOG numbers (for collapsed proteins of the corresponding tdCOG). The subtree which does not contain representatives of the tdCOGs is collapsed and labeled as “other proteins from COG0639”. The number of proteins for the corresponding tdCOG is shown in square blue brackets for species in the following order: *D. geothermalis*, *D. radiodurans*, and *T. thermophilis* strains HB27 and HB8. GI numbers: mlr2514 gi|13472274 ; AGc4222 gi|15889599 ; SMc01798 gi|15964990 ; BH1859 gi|15614422 ; SP0619 gi|15900527; SP1637 gi|15901473 ; alr1198 gi|17228693 ; alr1199 gi|17228694 ; AF1953 gi|11499535 ; PAB1723 gi|14521180 ; PH1004 gi|14590846 ; APES047 gi|14601304 ; VNG2372G gi|15791164 ; APE1169 gi|118431348 ; TVN1195 gi|13542026 ; Ta0989 gi|16082027 ; MA4202 gi|20092993 ; TM1876 gi|15644619 ; MK0804 gi|20094241 ; MJ0912 gi|15669102 ; PAB1999 gi|14520766 ; PH1616 gi|14591391 ; mlr6579 gi|13475494 ; HI0762 gi|16272703 ; PM1388 gi|15603253 ; FN1603 gi|19704924 ; Cj0184c gi|15791571 ; AF1822 gi|11499410 ; APE0777 gi|14600961 ; PAE2004 gi|18313027 ; MA3259 gi|20092075 ; SSO1090 gi|15897955.

**Supporting References**

[S1] Edgar RC (2004) MUSCLE: multiple sequence alignment with high accuracy and high throughput. Nucleic Acids Res 32: 1792-1797.

[S2] Tamura K, Dudley J, Nei M, Kumar S (2007) MEGA4: Molecular Evolutionary Genetics Analysis (MEGA) Software Version 4.0. [Mol Biol Evol.](javascript:AL_get(this, 'jour', 'Mol Biol Evol.');) May 7.
