# Supplementary material for: Deinococcus geothermalis: The Pool of Extreme Radiation Resistance Genes Shrinks
Source: PLoS One. 2007 Sep 26;2(9):e955. doi: 10.1371/journal.pone.0000955 (PMC1978522; doi:10.1371/journal.pone.0000955)
Supplement: Figure S6 — Structure of D. radiodurans homozygous mutants. (0.25 MB DOC) [file pone.0000955.s006.doc]

**Figure S6**

**Figure S6.** Structure of *D. radiodurans* homozygous mutants. **A**, DRB0100. **B**, DR2221. **C,** DR0105. **D,** DR0140. Within each panel: top left, predicted DNA band sizes of wild-type (ATCC BAA-816); bottom, predicted disrupted sequence following cleavage with the indicated restriction endonuclease; right, Southern blot analysis of indicated mutant using diagnostic ^32^P-labeled probes (wavy lines). ATG/GTG, start of gene. TGA, end of gene. Genes were disrupted by tandem-duplication insertion as described previously [S3] using pCR2.1 (Invitrogen, CA). Abbreviations: L, DNA size markers (kb); wt, wild-type (ATCC BAA-816); m, mutant.

**Supporting Reference**

[S3] Markillie LM, Varnum SM, Hradecky P, Wong KK (1999) Targeted mutagenesis by duplication insertion in the radioresistant bacterium *Deinococcus radiodurans*: radiation sensitivities of catalase (*katA*) and superoxide dismutase (*sodA*) mutants. J Bacteriol 181: 666-669.
