# Supplementary material for: Deinococcus geothermalis: The Pool of Extreme Radiation Resistance Genes Shrinks
Source: PLoS One. 2007 Sep 26;2(9):e955. doi: 10.1371/journal.pone.0000955 (PMC1978522; doi:10.1371/journal.pone.0000955)
Supplement: Figure S7 — The ESDSA model does not fully explain the early formation of covalently closed circular (ccc) derivatives of tandem duplications in irradiated D. radiodurans. (0.08 MB DOC) [file pone.0000955.s007.doc]

**Figure S7**

**Figure S7.** The ESDSA model does not fully explain the early formation of covalently closed circular (ccc) derivatives of tandem duplications in irradiated *D. radiodurans*. Very early in the recovery from IR, *D. radiodurans* DSB fragments containing duplication insertions of the configuration *X****AB****^1^CD****AB****^2^EFGH* yield ccc DNA molecules (monomers) (***AB****CD*) [S4]. In the test system developed by Daly and Minton [S4], segments **AB**^1^ and **AB**^2^ were identical sequences (4 kb each) in the same orientation separated by 18 kb of unique chromosomal DNA (*CD*). Following irradiation (17.5 kGy), ccc ***AB****CD* (22 kb) molecules were formed rapidly by a *recA*-independent mechanism, designated single-strand annealing (SSA). **A**, Circularization by SSA of a DSB fragment containing a tandem duplication. Following irradiation, there is exonucleolytic degradation of the 5’→3’ strands allowing for annealing of the remaining single strands. Nonhomologous ends are removed and gaps are filled in, using the opposite strand as a template, to complete the SSA reaction [S4]. **B**, In contrast, ESDSA [S5] might yield linear fragments with long 3’ single-stranded ends if the displaced strand does not contain single strand breaks (SSBs). However, since IR typically generates 40 times more SSBs than DSBs, ESDSA would be expected to encounter a SSB on the displaced strand, which might be converted to a ccc DNA molecule by a mechanism similar to that shown in panel A.

**Supporting References**

[S4] Daly MJ, Minton KW (1996) An alternative pathway of recombination of chromosomal fragments precedes recA-dependent recombination in the radioresistant bacterium *Deinococcus radioduran*s. J Bacteriol 178: 4461-4471.

[S5] Zahradka K, Slade D, Bailone A, Sommer S, Averbeck D, et al. (2006) Reassembly of shattered chromosomes in *Deinococcus radiodurans*. Nature 443: 569-573.
