# Supplementary material for: Deinococcus geothermalis: The Pool of Extreme Radiation Resistance Genes Shrinks
Source: PLoS One. 2007 Sep 26;2(9):e955. doi: 10.1371/journal.pone.0000955 (PMC1978522; doi:10.1371/journal.pone.0000955)
Supplement: Figure S8 — Multiple alignment comparisons for RecA proteins of the Thermus-Deinococcus group with selected representatives of other bacteria. (0.05 MB DOC) [file pone.0000955.s008.doc]

**Figure S8**

* 20 * 40 * 60 * 80 *

**58177549** : GSHMSKDATKEISAPTDAKERSKAIETAMSQIEKAFGKGSIMKLGAESK--LDVQVVSTGSLSLDLALGVGGIPRGRITEIYGPESGGKTTLALA : 93

94986237 : ---MSKDNPKDFGTPSDSKERLKAIETAMTQIEKAFGKGSIMRLGAESK--LDVQAVSTGSLSLDLALGVGGIPRGRITEIYGPESGGKTTLALS : 90

1350567 : ----------------MEENKRKSLENALKTIEKEFGKGAVMRLGEMPK--LQVDVIPTGSLGLDLALGIGGIPRGRVTEIFGPESGGKTTLALT : 77

47606740 : ----------------MDESKRKALENALKAIEKEFGKGAVMRLGEMPK--QQVDVIPTGSLALDLALGIGGIPRGRIVEIYGPESGGKTTLALT : 77

# # #

**55670286** : -----------GSHMAIDENKQKALAAALGQIEKQFGKGSIMRLGEDRS--MDVETISTGSLSLDIALGAGGLPMGRIVEIYGPESSGKTTLTLQ : 82

132233 : ----------------MDENKKRALAAALGQIERQFGKGAVMRMGDHER--QAIPAISTGSLGLDIALGIGGLPKGRIVEIYGPESSGKTTLTLS : 77

1172891 : ----------------MDENKQKALAAALGQIEKQFGKGSIMRLGDNRA--MDVETISTGSLSLDIALGAGGLPMGRIVEIFGPESSGKTTLTLE : 77

116333847 : ----------------MADERQAALDKALKKIEKDFGKGSIMRLGDNSN--LEVETVPSGSLALDVALGVGGYPRGRIVEIYGPESSGKTTVALH : 77

83589927 : ------------MQRVVINEKQRALEMALSQIERHFGKGSIMRLGETGA-RLNVEAISTGALPLDLALGVGGLPRGRVIEIFGPESSGKTTVALH : 82

50403565 : ----------------MTQEREKAIELALSQIEKQFGKGAIMRLGADEA-LPDVAAIPTGSLSLDLALGVGGVPRGRIIEIYGPESSGKTTLALH : 78

108759616 : --------------MAVNQEKEKAIELAMSAVERQFGKGSIMRLGNDEPMMRDVQAIPTGSISLDIALGVGGVPKGRIIEIFGPESSGKTTLCLH : 81

20178056 : ----MTSQAALKLVAKEEGDKQRALEAALAQIDRAFGKGSVMKLGEKGK--VEIESVSTGSLGLDIALGIGGLPKGRIVEVYGPESSGKTTLALH : 89

20178054 : -----MAQNSLRLVEDKAVDKSKALDAALSQIERAFGKGSIMRLGANEQ-VVEIETVPTGSLGLDIALGVGGLPRGRIIEIYGPESSGKTTLALH : 89

417633 : -----MAQNSLRLVEDKSVDKSKALEAALSQIERSFGKGSIMKLGSNEN-VVEVETVSTGSLSLDIALGIGGLPKGRIIEIYGPESSGKTTLALQ : 89

100 * 120 * 140 * 160 * 180 *

**58177549** : IVAQAQKAGGTCAFIDAEHALDPVYARALGVNTDELLVSQPDNGEQALEIMELLVRSGAIDVVVVDSVAALTPRAEIEGDMGDSLPGLQARLMSQ : 188

94986237 : VIAQAQRAGGTCAFIDAEHALDPVYARSLGVNTDELLVSQPDNGEQALEIMELLVRSGAIDVVVVDSVAALTPRAEIEGEMGDSLPGLQARLMSQ : 185

1350567 : IIAQAQKGGGVAAFVDAEHALDPLYAKKLGVDVQELLVSQPDTGEQALEIVELLARSGAVDVIVVDSVAALVPKAEIEGEMGDQHVGLQARLMSQ : 172

47606740 : IIAQAQRRGGVAAFVDAEHALDPLYAQRLGVQVEDLLVSQPDTGEQALEIVELLARSGAVDVIVVDSVAALVPRAEIEGEMGDQHVGLQARLMSQ : 172

#

**55670286** : VIAAAQREGKTCAFIDAEHALDPIYARKLGVDIDNLLCSQPDTGEQALEICDALARSGAVDVIVVDSVAALTPKAEIEGEIGDSHMGLAARMMSQ : 177

132233 : VIAEAQKQGATCAFVDAEHALDPDYAGKLGVNVDDLLVSQPDTGEQALEITDMLVRSNAVDVIIVDSVAALVPKAEIEGEMGDAHVGLQARLMSQ : 172

1172891 : LIAAAQREGKTCAFIDAEHALDPVYAKKLGVNIDELLVSQPDTGEQALEICDALARSGAVDVIVVDSVAALTPKAEIEGEMGDSHMGLQARMLSQ : 172

116333847 : AVAEVQKRGGTAAYIDAENALDPAYATALGVNIDDLLLSQPDTGEQGLQIADALISSGAIDIVVVDSVAALVPRAEIEGEMGDAHVGLQARLMSQ : 172

83589927 : VIAEAQRAGGTAAFIDAEHALDPVYAHNLGVDTDNLLVSQPDTGEQALEIAEALVRSGAIDVIVIDSVAALVPRAELEGEMGDAHVGLQARLMSQ : 177

50403565 : IAAEAQKMGGIAAFVDAEHALDIGYARKLGVKTDDLLVSQPDTGEQALEIAEMLVRSGAVDVLVIDSVAALVPKAEIEGEMGDSHMGLQARLMSQ : 173

108759616 : IVAEAQKRGGICGYVDAEHALDVGYARKLGVRTDDLLLSQPDTGEQALEIAEMLVRSGAIDVLVVDSVAALVPKAELEGEMGDAHMGVQARLMSQ : 176

20178056 : VVAEVQKAGGTAAFVDAEHALDPSYAYKLGVNLDNLLVSQPDNGEQALEITDTLVRSGAVDIVVVDSVAALTPKAEIEGEMGDSLPGLQARLMSQ : 184

20178054 : TVAEAQKKGGICAFVDAEHALDPVYARKLGVDLENLLISQPDTGEQALEICDTLVRSGAIDVLVVDSVAALTPRAEIEGEMGDSLPGLQARLMSQ : 184

417633 : TIAEAQKKGGICAFVDAEHALDPVYARKLGVDLQSLLISQPDTGEQALEITDTLVRSGAVDVLVIDSVAALTPRAEIEGEMGDSLPGLQARLMSQ : 184

200 * 220 * 240 * 260 * 280

**58177549** : ALRKLTAILSKTGTAAIFINQVREKIGVMYGNPETTTGGRALKFYASVRLDVRKIGQPTKVGNDAVANTVKIKTVKNKVAAPFKEVELALVYGKG : 283

94986237 : ALRKLTAILSKTGTAAIFINQVREKIGVMYGNPETTTGGRALKFYASVRLDVRKIGQPVKLGNDAVGNTVKVKTVKNKVAPPFKEVELTLLYGKG : 280

1350567 : ALRKLTAVLSKSNTAAIFINQVREKVGVMYGNPETTPGGRALKFYSSVRLDVRKSGQPIKVGNEAVGIKVKVKVVKNKLAPPFREAELEIYFGRG : 267

47606740 : ALRKLTAVLAKSNTAAIFINQVREKVGVTYGNPETTPGGRALKFYASVRLDVRKSGQPIKVGNEAVGVKVRVKVVKNKLAPPFREAELEIYFGRG : 267

# # ## #

**55670286** : AMRKLAGNLKQSNTLLIFINQIRMKIGVMFGNPETTTGGNALKFYASVRLDIRRIGA-VKEGENVVGSETRVKVVKNKIAAPFKQAEFQILYGEG : 271

132233 : ALRKITGNIKNANCLVIFINQIRMKIGVMFGNPETTTGGNALKFYASVRLDIRRTGA-VKEGDEVVGSETRVKVVKNKVSPPFRQAEFQILYGKG : 266

1172891 : AMRKLTGNLKQSNCMCIFINQIRMKIGVMFGNPETTTGGNALKFYASVRLDIRRTGA-IKEGEEVVGNETRIKVVKNKIAAPFKEANTQIMYGQG : 266

116333847 : ALRKLSGTINKTKTIALFINQIREKVGVMFGNPETTPGGRALKFYATVRLEVRRAEQ-IKDGTDVIGNRTRIKVVKNKVAPPFKRAEVDIMYGQG : 266

83589927 : ALRKLAGIISKSRTVAIFINQLREKVGVLFGNPETTPGGRALKFYASVRLDVRKVEQ-LKAGTEIVGNRTRVKVVKNKVAPPFRQAEFDIIYGRG : 271

50403565 : ALRKLTGIISKSNCCVIFINQIRMKIGVMFGNPETTTGGNALKFYASVRLDIRKIAS-LKQGQDVIGSRTKVKVVKNKVAPPFKEVEFDIYYGEG : 267

108759616 : ALRKLTGTIAKSQTCVIFINQIRMKIGVMFGNPETTTGGNALKFYASQRLDIRRIGA-IKNGDNVVGSRTRVKVVKNKVAPPFKEVEFDIMYGTG : 270

20178056 : ALRKLTASINKANTIVIFINQIRHKIGVMYGSPETTTGGNALKFYASVRLDIRRTGS-VKARDEIVGNNVRVKVVKNKVAPPFREVEFDIMYGEG : 278

20178054 : ALRKLTASISRSNTMVIFINQIRMKIGVMFGSPETTTGGNALKFYASVRLDIRRIGS-VKDRDEVVGNQTRVKVVKNKLAPPFKVVEFDIMYGEG : 278

417633 : ALRKLTASISKSKCMVIFINQIRMKIGVMFGSPETTTGGNALKFYASVRLDIRRIGA-VKEREEVVGNQTRVKVVKNKMAPPFKQVEFDIMYGEG : 278

* 300 * 320 * 340 * 360 * 380

**58177549** : FDQLSDLVGLAADMDIIKKAGSFYSYGDERIGQGKEKTIAYIAERPEMEQEIRDRVMAAIRAGNAGEAPALAPAPAAPEAAEA------------ : 366

94986237 : FDQLSDLVTLAADMDIIKKAGSFYSYGEERIGQGKEKAIAYIAERPELEQEIRDRVLAAIKEGRD----PIAAVPETPALAE------------- : 358

1350567 : LDPVMDLVNVAVAAGVIEKAGSWFSYGEHRLGQGKEKAAEYLRERPELLEEIRAKVLERADKV------VLAAGEEEGE---------------- : 340

47606740 : LDPVADLVNVAVAAGVIEKAGSWFSYGELRLGQGKEKAAEALRERPELLEEIRTKVLERAGEV------VLAAGEDEGE---------------- : 340

#

**55670286** : INFYGELVDLGVKEKLIEKAGAWYSYKGEKIGQGKANATAWLKDNPETAKEIEKKVRELLLSNPNS---TPDFSVDDSEGVAETNEDF------- : 356

132233 : IYRTGEIIDLGVQLGLVEKSGAWYSYQGSKIGQGKANAAKYLEDNPEIGSVLEKTIRDQL---------LAKSGPVKADAEEVADAEAD------ : 346

1172891 : FNREGELIDLGVKHKMVEKSGAWYSYNGDKIGQGKANACKYLKENPEIAKTLDKKLREML-LNPENMQLIAETSSAADDVEFGAVPEEF------ : 354

116333847 : ISQTGELLDMAVEKDIVDKSGSWYSYGEDRIGQGRENAKQYLADHPDMMAEVNQRVRAAYGVGDEE---AAATKATETKTDAPKDKDKGKTKAKD : 358

83589927 : IDREGCLLDMGTELDIIKKSGAWYSLGEDRLGQGREAAKDFLREHPDLAAALETKIREKAGLIN-----FTAGKEDATSGED------------- : 348

50403565 : ISREGDILDLAVEKGIVDKSGAWFSYGGDRIGQGRENSRLFLKERPELVNEIEGKVYDVAGI-------PRKGAKEAA----------------- : 338

108759616 : ISREGDLIDLASNENIVEKSGSWFSFNGERIGQGRENVKEYLREHPEIAKDIEGRVLEKYGIGKSGA--PVAAAPDESAPAEGGSEKRGRVKAVK : 363

20178056 : ISKLGEVIDLGVKAGIIDKAGSWFSYGSQRIGQGRDNVREFLKNNPDVAADIEKAVRKSSQKIEEE---LLVGGPEEGEED-------------- : 356

20178054 : VSKTGELVDLGVKAGVVEKSGAWFSYNSQRLGQGRENAKLFLRDNPDTAREIELALRQNAGLIAEKF--LENGGSEGGDDGFEDEAGAM------ : 365

417633 : VSKTGELVDLGVKAGIVEKSGAWFSYNSQRLGQGRENAKTFLRDNPDTANEIELALRQNAGLIADRF--LQNGGPDAGEGDDGSDEG-------- : 363

**Figure S8.** Multiple alignment comparisons for RecA proteins of the *Thermus-Deinococcus* group with selected representatives of other bacteria. The alignments were built using the MUSCLE program [S1]. Protein sequences are labeled by their GI numbers as follows: 58177549 - RecA, *D. radiodurans* (Pdb:1XP8); 94986237 - RecA, *D. geothermalis*; 1350567 - RecA, *Thermus aquaticus*; 47606740 - RecA, *T. thermophilus*; 55670286 - RecA, *Escherichia coli* (Pdb:1U94); 132233 - RecA, *Pseudomonas aeruginosa*; 1172891 - RecA, *Vibrio cholerae*; 116333847 - RecA, *Lactobacillus brevis*; 83589927 - RecA, *Moorella thermoacetica*; 50403565 - RecA, *Geobacter sulfurreducens*; 108759616 - RecA, *Myxococcus xanthus*; 20178056 - RecA, *Caulobacter crescentus*; 20178054 - RecA, *Mesorhizobium loti*; 417633 - RecA, *Agrobacterium tumefaciens*. Two proteins for which crystal structures are available are shown in red. Amino acid positions that were found previously by Rajan and Bell [S6] to be different for *D. radiodurans* and *E. coli* are shown in cyan and green; cyan for moderately conserved positions, and green for strongly conserved positions. The pound symbol (#) indicates amino acids with similar chemical properties in the *Deinococcus* and *Thermus* lineages.

**Supporting References**

[S1] Edgar RC (2004) MUSCLE: multiple sequence alignment with high accuracy and high throughput. Nucleic Acids Res 32: 1792-1797.

[S6] Rajan R, Bell CE (2004) Crystal structure of RecA from *Deinococcus radiodurans*: insights into the structural basis of extreme radioresistance. J Mol Biol 344: 951-963.
