# Supplementary material for: Deinococcus geothermalis: The Pool of Extreme Radiation Resistance Genes Shrinks
Source: PLoS One. 2007 Sep 26;2(9):e955. doi: 10.1371/journal.pone.0000955 (PMC1978522; doi:10.1371/journal.pone.0000955)
Supplement: Figure S9 — Chrome azurol S agar plate assay for siderophore production. (0.13 MB DOC) [file pone.0000955.s009.doc]

**Figure S9**


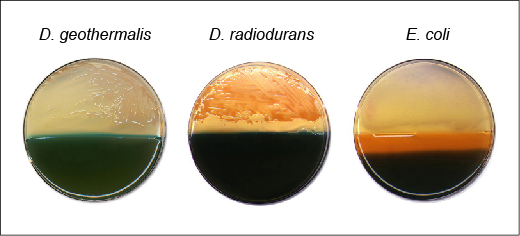


**Figure S9**. Chrome azurol S agar plate assay for siderophore production. The indicated strains were tested for their ability to secrete siderophores as described by others [S7]. *D. geothermalis* (negative), *D. radiodurans* (negative) and *E. coli* (positive). The color-change of azurol from blue to red occurs in the presence of siderophores.

**Supporting Reference**

[S7] [Milagres AM, Machuca A, Napoleao D (1999)](http://www.ncbi.nlm.nih.gov/sites/entrez?Db=pubmed&Cmd=ShowDetailView&TermToSearch=10395458&ordinalpos=5&itool=EntrezSystem2.PEntrez.Pubmed.Pubmed_ResultsPanel.Pubmed_RVDocSum) Detection of siderophore production from several fungi and bacteria by a modification of chrome azurol S (CAS) agar plate assay.
J Microbiol Methods 37: 1-6.
