# Supplementary material for: Deinococcus geothermalis: The Pool of Extreme Radiation Resistance Genes Shrinks
Source: PLoS One. 2007 Sep 26;2(9):e955. doi: 10.1371/journal.pone.0000955 (PMC1978522; doi:10.1371/journal.pone.0000955)
Supplement: Figure S10 — Whereas the nramp gene of D. radiodurans is essential, the fur gene is dispensable. (0.13 MB DOC) [file pone.0000955.s010.doc]

**Figure S10**


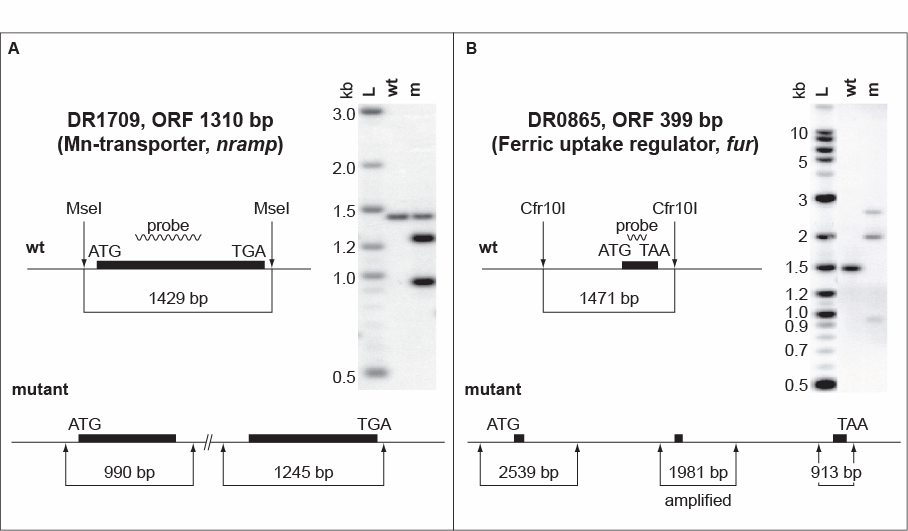


**Figure S10.** Whereas the *nramp* gene of *D. radiodurans* is essential, the *fur* gene is dispensable. Structure of *D. radiodurans* mutants. **A,** DR1709 (*nramp*) (heterozygous). Note, the diagnostic wild-type 1429 bp band remains in the mutant. **B,** DR0865 (*fur*) (homozygous). Note, the wild-type 1471 bp band is absent. Within each panel: top left, predicted DNA band sizes of wild-type (ATCC BAA-816); bottom, predicted disrupted sequence following cleavage with the indicated restriction endonuclease; right, Southern blot analysis of indicated mutant using diagnostic ^32^P-labeled probes (wavy lines). ATG, start of gene. TGA/TAA, end of gene. Genes were disrupted by tandem-duplication insertion as described previously [S3] using pCR2.1 (Invitrogen, CA). Abbreviations: L, DNA size markers (kb); wt, wild-type (ATCC BAA-816); m, mutant.

**Supporting Reference**

[S3] Markillie LM, Varnum SM, Hradecky P, Wong KK (1999) Targeted mutagenesis by duplication insertion in the radioresistant bacterium *Deinococcus radiodurans*: radiation sensitivities of catalase (*katA*) and superoxide dismutase (*sodA*) mutants. J Bacteriol 181: 666-669.
