# Supplementary material for: Deinococcus geothermalis: The Pool of Extreme Radiation Resistance Genes Shrinks
Source: PLoS One. 2007 Sep 26;2(9):e955. doi: 10.1371/journal.pone.0000955 (PMC1978522; doi:10.1371/journal.pone.0000955)
Supplement: Table S1 — Homology between the D. radiodurans and D. geothermalis megaplasmids. (0.04 MB DOC) [file pone.0000955.s011.doc]

**Table S1.** Homology between the *D. radiodurans* and *D. geothermalis* megaplasmids

|  | **DR chromosome** | **DR412** | **DR177** | **DR plasmid** |
| --- | --- | --- | --- | --- |
| **DG chromosome** | 1731^A^ (1650.5)^B^ | 101^A^ (163.9) ^B^ | 21^A^ (38.0)^B^ | 4^A^ (4.6)^B^ |
| **DG574** | 51^A^ (131.5)^B^ | 76^A^ (13.1)^B^ | 20^A^ (3.0)^B^ | 1^A^ (0.4)^B^ |
| ^C^ **P(χ^2^) = 1.1x10^-104^** | | | | |

^A^The observed number of orthologous proteins encoded in genome partitions of *D. radiodurans* (DR) and *D. geothermalis* (DG). ^B^In parentheses, the expected number of orthologs under the assumption of independent orthologous relationships with genome location. The significant excess of orthologs encoded in the *D. radiodurans* megaplasmids DR412 and DR177, and in the *D. geothermalis* megaplasmid DG574 indicates homologous relationships between DG574 and the two *D. radiodurans* megaplasmids. ^C^(χ^2^) indicates probability of the deviation of the observed values from their expectations according to the χ^2^ statistics.
