# Supplementary material for: Deinococcus geothermalis: The Pool of Extreme Radiation Resistance Genes Shrinks
Source: PLoS One. 2007 Sep 26;2(9):e955. doi: 10.1371/journal.pone.0000955 (PMC1978522; doi:10.1371/journal.pone.0000955)
Supplement: Table S3 — Lineage specific expansion of selected families in D. geothermalis (DG), D. radiodurans (DR), T. thermophilus HB27 (TT27), and T. thermophilus HB8 (TT8). (0.05 MB DOC) [file pone.0000955.s013.doc]

**Table S3.** Lineage specific expansion of selected families in *D. geothermalis* (DG), *D. radiodurans* (DR), *T. thermophilus* HB27 (TT27), and *T. thermophilus* HB8 (TT8)

| ^A^**Cluster** | **Number of Representatives in DG** | **Number of Representatives in DR** | **Number of Representatives in TT27** | **Number of Representatives in TT8** | **COG**  **Numbers** | **Protein Name** |
| --- | --- | --- | --- | --- | --- | --- |
| tdCOG00395 | 2 | 6 | 1 | 1 | COG00494 | NUDIX hydrolase |
| tdCOG01008 | 5 | 2 | 1 | 1 | COG00640 | Transcriptional regulator, ArsR family |
| tdCOG02522 | 2 | 5 | 1 | 1 | COG02340 | SCP/PR1 domain |
| tdCOG01508 | 2 | 7 | 0 | 0 | COG00454 | GCN5-related N-acetyltransferase |
| tdCOG01431 | 1 | 5 | 0 | 0 | COG00454 | GCN5-related N-acetyltransferase |
| tdCOG01391 | 2 | 4 | 0 | 0 | COG00563 | P-loop ATPase related to DNA topology modulation kinase FlaR |
| tdCOG02792 | 2 | 2 | 0 | 0 |  | Yfit/DinB family protein |
| tdCOG02705 | 1 | 3 | 0 | 0 | COG01073 | alpha/beta hydrolase fold |
| tdCOG01474 | 1 | 3 | 0 | 0 | COG00494 | NUDIX hydrolase |
| tdCOG02675 | 2 | 2 | 0 | 0 | COG00657 | alpha/beta superfamily hydrolase |
| tdCOG01839 | 1 | 3 | 0 | 0 | COG01670 | GCN5-related N-acetyltransferase |
| tdCOG01399 | 3 | 1 | 0 | 0 | COG01051 | NUDIX hydrolase |

^A^Clusters of orthologous groups of proteins (COGs) for *Deinococcus* and *Thermus* (tdCOGs)
