# Supplementary material for: Deinococcus geothermalis: The Pool of Extreme Radiation Resistance Genes Shrinks
Source: PLoS One. 2007 Sep 26;2(9):e955. doi: 10.1371/journal.pone.0000955 (PMC1978522; doi:10.1371/journal.pone.0000955)
Supplement: Table S4 — Protein families expanded in D. geothermalis. (0.05 MB DOC) [file pone.0000955.s014.doc]

**Table S4.** Protein families expanded in *D. geothermalis*

| **Description** | **COG Numbers** | **Number of Representatives in DG** | **Number of Representatives in DR** | **Number of Representatives in TT (HB27)** |
| --- | --- | --- | --- | --- |
| Predicted oxidoreductases (related to aryl-alcohol dehydrogenases) | COG0667 | 12 | 4 | 1 |
| Glycosidases | COG0366 | 7 | 6 | 3 |
| Xylanase/chitin deacetylase | COG0726 | 6 | 3 | 1 |
| Sugar kinases | COG0524 | 6 | 5 | 3 |
| Sugar (pentulose and hexulose) kinases | COG1070 | 4 | 0 | 0 |
| Beta-xylosidase | COG3507 | 2 | 0 | 0 |
| ABC-type sugar transport systems, ATPase components | COG3839 | 2 | 1 | 3 |
| ABC-type sugar transport systems, permease components | COG1175 | 12 | 2 | 6 |
| ABC-type sugar transport system, periplasmic component | COG1653 | 11 | 1 | 8 |
| ABC-type sugar transport system, permease component | COG0395 | 11 | 1 | 6 |
| AraC-type DNA-binding domain-containing proteins | COG2207 | 3 | 0 | 0 |
| IclR–like transcriptional regulator | COG1414 | 7 | 3 | 2 |
| Transcriptional regulator/sugar kinase | COG1940 | 5 | 2 | 2 |
| Glycosyltransferase | COG0438 | 11 | 8 | 7 |
| Cation transport ATPase | COG2217 | 6 | 3 | 3 |
