# Supplementary material for: Deinococcus geothermalis: The Pool of Extreme Radiation Resistance Genes Shrinks
Source: PLoS One. 2007 Sep 26;2(9):e955. doi: 10.1371/journal.pone.0000955 (PMC1978522; doi:10.1371/journal.pone.0000955)
Supplement: Table S5 — Protein families expanded in D. radiodurans. (0.07 MB DOC) [file pone.0000955.s015.doc]

***Table S5*** *Protein families expanded in D. radiodurans*

| **Description** | **COG Numbers** | **Number of Representatives in DR** | **Number of Representatives in DG** | **Number of Representatives in TT(HB27)** |
| --- | --- | --- | --- | --- |
| **Widespread families expanded in DR** | | | | |
| Nudix (MutT-like) phosphohydrolases | COG0494 COG1051 | 17  5 | 7  7 | 5  5 |
| Lipase-like alpha/beta hydrolase | COG0596 COG0400 COG1073 COG1075 COG0657 | 10  1  8  2  4 | 8  1  4  0  3 | 6  1  1  0  0 |
| Subtilisin-like protease | COG1404 | 10 | 7 | 2 |
| Methyltransferase | COG0500 | 14 | 6 | 5 |
| DNA modification methyltransferase | COG1002 | 5 | 1 | 1 |
| Acetyltrasferases GNAT family | COG0454 COG0497 COG1610 | 26  10  11 | 11  8  8 | 2  3  3 |
| DinB/YfiT family | COG2318; no COG | 3  10 | 2  6 | 1  0 |
| PadR-like transcriptional regulators | COG1695 | 9 | 1 | 0 |
| AcrR-like transcriptional regulators | COG1309 | 15 | 7 | 4 |
| Roadblock/LC7 domain | COG2018 | 4 | 1 | 2 |
| MOSC sulfur-carrier domains | COG2258 | 4 | 2 | 1 |
| KatE-like catalase | COG0753 | 3 | 1 | 0 |
| FlaR like kinases | COG0593 | 5 | 3 | 1 |
| Adenylate kinase  family | COG0645 | 4 | 0 | 0 |
| McrA endonuclease | COG1403 | 5 | 2 | 1 |
| TerZ family | COG2310 | 7 | 0 | 0 |
| WD-40 repeats | COG1520 | 6 | 2 | 1 |
| PR1 family | COG2340 | 5 | 2 | 1 |
| DR0104-like transcriptional regulators | - | 5 | 0 | 0 |
| **Unique DR families** | | | | |
| GRXGG repeats containing protein | **-** | DR0082, DR2593, DR1748 | Dgeo_0413,  Dgeo_0507 | No,  homologs in *Symbiobacterium thermophilum* |
| Alpha/beta proteins, tryptophan-rich | **-** | DR2532, DR2457 | - | No |
| Proteins with GXTXXXG and CXPXXXC motifs (DR0871 has duplication of the domain) | **-** | DR0871, DR1920, DR2360 | - | No |
| Secreted alpha/beta proteins with a single conserved domain | - | DR1251, DR1319, DR1545 | - | No |
| Predominantly alpha-helical proteins | **-** | DR0481, DR1195, DR1301 | Dgeo_0865 | No |
| Predominantly alpha-helical proteins | **-** | DR0387, DR2038+DR2039 | - | No |
| Predicted metabolic regulator containing V4R domain | - | DR2179, DR1611 | - | No |
