# Supplementary material for: Deinococcus geothermalis: The Pool of Extreme Radiation Resistance Genes Shrinks
Source: PLoS One. 2007 Sep 26;2(9):e955. doi: 10.1371/journal.pone.0000955 (PMC1978522; doi:10.1371/journal.pone.0000955)
Supplement: Table S6 — Gene context and motifs of predicted cytoplasmic proteins shared by two Deinococcus species, but for which homologs outside the lineage do not exist. (0.17 MB DOC) [file pone.0000955.s016.doc]

**Table S6.** Gene context and motifs of predicted cytoplasmic* proteins shared by two *Deinococcus* species, but for which homologs

outside the lineage do not exist

| **tdCOG number**^A^ | **Orthologs**^A^ **in DG** | Orthologs^A^ in DR | **Protein Description,**  **(COG number)** | **Comments** |
| --- | --- | --- | --- | --- |
| tdCOG01264 | Dgeo_0029 | DR0017 | Predicted transcriptional regulator, HTH domain of Fis family (COG3877). Other proteins from this family have a Zn-ribbon domain, indicating a possible DNA-binding function | This operon and homologs of Dgeo_0031 (which are Dgeo_1707, DR1557) are implicated in some specific processes related to DNA or RNA repair/replication or modification. Specifically, because Fis-related proteins are known to be involved in such processes in other bacteria [S8], and because of potential co-regulation (divergent operon) with Dgeo_0032, a VacB-type ribonuclease. |
| tdCOG01265 | **Dgeo_0030** | **DR0018** | Uncharacterized protein |
| tdCOG01266 | **Dgeo_0031** | **DR0019** | Uncharacterized protein |
| tdCOG01631 | Dgeo_1706 | DR1556 | Periplasmic sensor signal transduction histidine kinase with GAF domain (COG4585) | Homologs of Dgeo_1707 (Dgeo_0031/DR0019) are associated with RNA nucleases, suggesting some role in RNA (or DNA) modification or repair. This operon may be involved in regulation of these pathways. |
| tdCOG01632 | **Dgeo_1707** | **DR1557** | Uncharacterized protein |
| tdCOG01633 | Dgeo_1708 | DR1558 | Two component transcriptional regulator (REC and HTH domains), LuxR family (COG2197) |
| tdCOG01666 | **Dgeo_0142** | **DR1697** | Uncharacterized protein |  |
| tdCOG01346 | **Dgeo_0209** | **DR0338** | Uncharacterized protein |  |
| tdCOG01376 | **Dgeo_320** | **DR0437** | Uncharacterized protein | Possibly, these genes are co-regulated with a conserved cluster (Dgeo_317-Dgeo_319 and orthologs in *D. radiodurans*) of genes involved in respiratory chain assembly or functioning. |
| tdCOG02716 | **Dgeo_0191** | **DR2230** | Uncharacterized protein | Probably, involved in system dedicated to catabolic/cell cleaning. |
| tdCOG00996 | Dgeo_0192 | DRA0031 | dTDP-glucose pyrophosphorylase related nucleotidyl transferase (COG1209) |
| tdCOG00247 | Dgeo_0193 | DR0654 | Alpha/beta hydrolase family enzyme (COG0596) |
| tdCOG01453 | **Dgeo_0194** | **DR0780** | Uncharacterized protein | Might be co-regulated with the operon above Dgeo_0029 - Dgeo_0031 (divergent operons). |
| tdCOG01261 | **Dgeo_0047** | **DR0003** | Uncharacterized protein, DdrC gene | The mutant is sensitive to radiation and the DR0003 is induced after irradiation [S9]. |
| tdCOG01279 | **Dgeo_0295** | **DR0070** | Uncharacterized protein, DdrB gene | The mutant is sensitive to radiation and DR0070 is induced after irradiation [S9]. |
| tdCOG02722 | **Dgeo_0113** | **DR2378** | Uncharacterized protein | DR2378 is an apparent homolog of DR0331. DR2378 is also a close homolog of Dgeo_0042/DRA0006, which is located in an operon together with a Zn-dependent catabolic enzyme alcohol dehydrogenase (DRA0005). |
| tdCOG01823 | Dgeo_0114 | DR2377 | Membrane-associated cyclase/dehydrase family protein (COG5637) |
| tdCOG01810 | **Dgeo_0383** | **DR2314** | Uncharacterized protein | Possible operon in *D. geothermalis*. |
| tdCOG02715 | **Dgeo_0384** | **DR2229** | Uncharacterized protein |
| tdCOG01273 | **Dgeo_0418** | **DR0047** | Uncharacterized protein | Possibly, involved in RNA metabolism. It might be co-regulated with a Zn-ribbon-containing protein DR0048 and a RNA methyltransferase containing a PUA domain (DR0049). |
| tdCOG01742 | **Dgeo_0440** | **DR2001** | Uncharacterized protein | In *D. geothermalis*, this gene is co-localized with Dgeo_440, a SARP family transcriptional regulator which is involved in the regulation of antibiotic production in *Streptomyces* species [S10]. In *D. radiodurans*, it is probably co-regulated with DR2001, a DinB/YfiT family protein. Thus, the product of this gene might be involved in cell-cleaning or stress response. |
| tdCOG01351 | **Dgeo_0477** | **DR0920** | Uncharacterized protein |  |
| tdCOG02681 | **Dgeo_0462** | **DR0355** | Uncharacterized protein |  |
| tdCOG01314 | **Dgeo_0482** | **DR0212** | Uncharacterized protein | Has a potential metal-binding motif CXXCXXHXH. In both species, this gene is co-localized with a membrane protein of the SanA family, involved in antibiotic resistance [S11]. |
| tdCOG02689 | **Dgeo_0501** | **DR1331** | Uncharacterized protein |  |
| tdCOG02800 | Dgeo_0510 | DRA0366 and  DRA0365 | Uncharacterized protein | Has homologs in other bacteria. In *D. radiodurans*, it is co-localized with membrane biogenesis enzymes (DRA0367, UDP-galactopyranose mutase; and DRA0368, Lipopolysaccharide glycosyltransferase). |
| tdCOG02677 | **Dgeo_0511** | **DR0869** | Uncharacterized protein |
| tdCOG02702 | **Dgeo_0542** | **DR1693** | Uncharacterized protein |  |
| tdCOG00812 | **Dgeo_0879** | **DR2156** | Uncharacterized protein | Dgeo_0879/DR2156 have orthologs in *Thermus*. |
| tdCOG01772 | **Dgeo_0880** | **DR2157** | Uncharacterized protein |
| tdCOG01773 | **Dgeo_0881** | **DR2158** | Uncharacterized protein |
| tdCOG01646 | **Dgeo_0935** | **DR1607** | Uncharacterized protein | Supporting a role in secondary metabolism or cell-cleaning pathways in *D. radiodurans*, the protein is probably co-regulated with DR1608, a DinB/YfiT family protein; and DR1609, a 2-keto-4-pentenoate hydratase-related enzyme. |
| tdCOG02671 | **Dgeo_1022** | **DR0734** | Uncharacterized protein |  |
| tdCOG02234 | Dgeo_1030 | DR1778 | 3-Isopropylmalate dehydratase large subunit (COG0065) | Leucine biosynthesis subunits. |
| tdCOG01689 | **Dgeo_1031** | **DR1781 and**  **DR1780** | Uncharacterized protein |
| tdCOG01690 | Dgeo_1032 | DR1784 | 3-Isopropylmalate dehydratase, small subunit (COG0065) |
| tdCOG00673 | Dgeo_1033 | DR1785 | 3-Isopropylmalate dehydrogenase(COG0473) |
| tdCOG01566 | **Dgeo_1085** | **DR1245** | Uncharacterized protein | Cell wall degradation subunits. |
| tdCOG01465 | Dgeo_1086 | DR0848 | Cell-wall associated protease, NlpD-like (COG0739) |
| tdCOG00471 | Dgeo_1087 | DR1246 | Peptidase M24 (COG0006) |
| tdCOG01628 | **Dgeo_1167** | **DR1539** | Uncharacterized protein | Metal-binding motif HXXXH, probably a metal-dependent hydrolase. |
| tdCOG01605 | **Dgeo_1220** | **DR1416** | Uncharacterized protein |  |
| tdCOG01256 | **Dgeo_1360** | **DRC0030** | Uncharacterized protein |  |
| tdCOG01524 | **Dgeo_1382** | **DR1121** | Uncharacterized protein | Possibly related to energy-conversion processes. |
| tdCOG01523 | Dgeo_1383 | DR1120 | Acetate or butyrate kinase (COG3426) |
| tdCOG01612 | **Dgeo_1393** | **DR1432** | Uncharacterized protein |  |
| tdCOG01728 | **Dgeo_1399** | **DR1896** | Uncharacterized protein |  |
| tdCOG01691 | **Dgeo_1429** | **DR1786** | Uncharacterized protein | In *D. radiodurans*, it is co-localized with alanine dehydrogenase (DR1785), an enzyme for alanine catabolism. Indicating a related function, GntR-like regulators apparently can regulate amino acid catabolism-related processes [S12]. |
| tdCOG01692 | Dgeo_1430 | DR1043 | transcriptional regulator, GntR family (COG1167) |
| tdCOG01693 | **Dgeo_1431** | **DR1788** | Uncharacterized protein |  |
| tdCOG01522 | **Dgeo_1437** | **DR1116** | Uncharacterized protein |  |
| tdCOG01707 | **Dgeo_1465** | **DR1831** | Uncharacterized protein |  |
| tdCOG01706 | **Dgeo_1466** | **DR1830** | Uncharacterized protein |
| tdCOG01647 | **Dgeo_1472** | **DR1615** | Uncharacterized protein |  |
| tdCOG01456 | **Dgeo_1478** | **DR0795** | Uncharacterized protein |  |
| tdCOG01755 | **Dgeo_1527** | **DR2077** | Uncharacterized protein | Possible cell-cleaning/stress response-related system. |
| tdCOG00781 | Dgeo_1528 | DR2076 | Beta-lactamase-like protein (COG0491) |
|  | Dgeo_1529 |  | Calcineurin-like phosphoesterase (COG0622) |
| tdCOG01424 | **Dgeo_1639** | **DR0637** | Uncharacterized protein |  |
| tdCOG01425 | Dgeo_1640 | DR0638 | Uncharacterized protein, putative membrane protein, no homologs |
| tdCOG01711 | **Dgeo_1651** | **DR1840** | Uncharacterized protein |  |
| tdCOG01319 | **Dgeo_1778** | **DR0239** | Uncharacterized protein |  |
| tdCOG02710 | **Dgeo_1783** | **DR2020** | Uncharacterized protein | Contains the metal-binding motif CXXC. |
| tdCOG02796 | **Dgeo_1814** | **DR0877** | Uncharacterized protein | A G-rich protein. |
| tdCOG02678 | **Dgeo_1823** | **DR0887** | Uncharacterized protein |  |
| tdCOG02709 | **Dgeo_1824** | **DR1994** | Uncharacterized protein |  |
| tdCOG02043 | Dgeo_1883 | DR1960 | Acetyl-CoA C-acetyltransferase (COG0183) | Possible membrane biogenesis operon. |
| tdCOG02706 | Dgeo_1884 | DR1961 | Ankyrin repeat protein (COG0666) |
| tdCOG01738 | **Dgeo_1885** | **DR1962** | Uncharacterized protein |
| tdCOG01416 | **Dgeo_1997** | **DR0600** | Uncharacterized protein |  |
| tdCOG01445 | **Dgeo_2024** | **DR0746** | Uncharacterized protein | Possible protease associated component. |
| tdCOG00278 | Dgeo_2025 | DR0745 | peptidase S1/S6, chymotrypsin/Hap (COG0265) |
| tdCOG02714 | **Dgeo_2044** | **DR2207** | Uncharacterized protein |  |
| tdCOG02672 | **Dgeo_2088** | **DR0800** | Uncharacterized protein |  |
| tdCOG01734 | **Dgeo_2113** | **DR1936** | Uncharacterized protein | Proline-rich, internal repeats. |
| tdCOG01344 | **Dgeo_2186** | **DR0326** | Uncharacterized protein, DdrD gene | The mutant is sensitive to IR and DR0326 is induced after irradiation [S9]. |
| tdCOG02717 | **Dgeo_2192** | **DR2237** | Uncharacterized protein |  |
| tdCOG02707 | **Dgeo_2200** | **DR1987** | Uncharacterized protein |  |
| tdCOG00066 | Dgeo_2266 | DR0123 | Phosphoribosylanthranilate isomerase (COG0135) | Possibly, involved in aromatic acid metabolism. |
| tdCOG01295 | **Dgeo_2267** | **DR0124** | Uncharacterized protein |
| tdCOG01925 | **Dgeo_2285** | **DRA0141** | Uncharacterized protein |  |
| tdCOG02249 | Dgeo_2320 | DR0849 | Uncharacterized protein | Possibly, involved in ubiquinone metabolism. |
| tdCOG01466 | **Dgeo_2321** | **DR0850** | Uncharacterized protein |
| tdCOG00319 | Dgeo_2322 | DR0851 | 4-hydroxybenzoate polyprenyltransferase (COG0382) |
| tdCOG02728 | **Dgeo_2325** | **DR2558** | Uncharacterized protein |  |
| tdCOG01960 | Dgeo_2417 | DRA0222 | NAD-dependent epimerase/dehydratase (COG0451) | Probable catechol degradation pathway components. |
| tdCOG01029 | Dgeo_2416 | DRA0220 | 5-Carboxymethyl-2-hydroxymuconate semialdehyde dehydrogenase (COG1012) |
|  | Dgeo_2415 | DR0109 | Lactoylglutathione lyase-related protein (COG0346) |
| tdCOG01959 | **Dgeo_2414** | **DRA0219** | Uncharacterized protein |
| tdCOG01033 | Dgeo_2413 | DRA0226 | 4-Hydroxyphenylacetate degradation bifunctional isomerase/decarboxylase (COG0179) |
| tdCOG01980 | **Dgeo_2628** | **DRA0346** | DNA damage repair protein PprA | The corresponding mutant is sensitive to IR. DRA0346 is induced after irradiation [S9]. *In vitro*, it preferentially bound double-stranded DNA carrying strand breaks, inhibited *E. coli* exonuclease III activity, and stimulated the DNA end-joining reaction catalysed by ATP-dependent and NAD-dependent DNA ligases [S13]. |

^*^Rare tdCOGs (109) predicted to be secreted or membrane proteins were not considered here, because such proteins have not previously been strongly implicated in radiation or desiccation resistance.

^A^The conserved tdCOG and the corresponding protein for the set of interest (see main text) are shown in red and bold typeface, respectively. Other tdCOGs which are in a designated predicted operon are in regular font.

**Supporting References**

[S8] Yuan HS, Finkel SE, Feng JA, Kaczor-Grzeskowiak M, Johnson RC, Dickerson RE, et al (1991) The molecular structure of wild-type and a mutant Fis protein: relationship between mutational changes and recombinational enhancer function or DNA binding. [Proc Natl Acad Sci U S A](javascript:AL_get(this, 'jour', 'Proc Natl Acad Sci U S A.');) 88: 9558-9562.

[S9] Tanaka M, Earl AM, Howell HA, Park MJ, Eisen JA, et al. (2004) Analysis of *Deinococcus radiodurans*'s transcriptional response to ionizing radiation and desiccation reveals novel proteins that contribute to extreme radioresistance. Genetics 168: 21-33.

[S10] Bate N, Stratigopoulos G, Cundliffe E (2002) Differential roles of two SARP-encoding regulatory genes during tylosin biosynthesis. [Mol Microbiol](javascript:AL_get(this, 'jour', 'Mol Microbiol.');) 43: 449-458.

[S11] Rida S, Caillet J, Alix JH (1996) Amplification of a novel gene, *sanA*, abolishes a vancomycin-sensitive defect in *Escherichia coli*. [J Bacteriol](javascript:AL_get(this, 'jour', 'J Bacteriol.');) 178: 94-102.

[S12] Gerischer U (2002) Specific and global regulation of genes associated with the degradation of aromatic compounds in bacteria [J Mol Microbiol Biotechnol](javascript:AL_get(this, 'jour', 'J Mol Microbiol Biotechnol.');) 4: 111-121.

[S13] Narumi I, Satoh K, Cui S, Funayama T, Kitayama S, et al. (2004) PprA: a novel protein from *Deinococcus radiodurans* that stimulates DNA ligation. Mol Microbiol 54: 278-285.
