# Supplementary material for: Deinococcus geothermalis: The Pool of Extreme Radiation Resistance Genes Shrinks
Source: PLoS One. 2007 Sep 26;2(9):e955. doi: 10.1371/journal.pone.0000955 (PMC1978522; doi:10.1371/journal.pone.0000955)
Supplement: Table S7 — Stress response-related genes in D. radiodurans (DR), D. geothermalis (DG) and T. thermophilus (TT). (0.23 MB DOC) [file pone.0000955.s017.doc]

***Table S7.*** *Stress response-related genes in D. radiodurans (DR), D. geothermalis (DG) and T. thermophilus (TT)*

| **Type of Stress** | **Protein Description and Comments** | **Gene name** | **DR gene_ID** | **DG gene_ID** | **TT gene_ID** | | **COG** |
| --- | --- | --- | --- | --- | --- | --- | --- |
| Heat/ general | Heat Shock Protein 10, molecular chaperone | GroL | DR0607 | Dgeo_2231 | TTC1714 | | COG0459 |
| Heat/ general | Heat Shock Protein 20, molecular chaperone | GrpE | DR0128 | Dgeo_2077 | TTC1126 | | COG0576 |
| Heat/ general | Heat Shock Protein 60, molecular chaperone | GroS | DR0606 | Dgeo_2230 | TTC1713 | | COG0234 |
| Heat/ general | Heat Shock Protein 70, molecular chaperone | DnaK | DR0129 | Dgeo_2076 | TTC1127 | | COG0443 |
| Heat/ general | Hsp70 chaperone co-factor | DnaJ | DR0126; DR1424 | Dgeo_2078 Dgeo_0451 | TTC1812 | | COG2214  COG0484 |
| Heat/ general | Small heat-shock protein | IbpA/ IbpB | DR1114 DR1691 | Dgeo_0505 Dgeo_0859 | TTC1116 TTC1120 TTC1257 TTC0203 | | COG0071 |
| Heat | Related to heat shock protein, HSLJ; DR1940 contains 3 repeats of this domain | HSLJ | DR2056 DR1940 | - | - | | COG3187 |
| General | ATPase subunit of Clp protease | ClpA/ ClpB | DR0588 DR1046 DR1117 | Dgeo_1475 Dgeo_1210 Dgeo_1215  Dgeo_2867 | TTC1123 | | COG0542 |
| General | ATPase subunit of Clp protease | ClpX | DR1973; DR0202  (+4 paralogs) | Dgeo_2152 Dgeo_0702 (+2 paralogs) | TTC0251 | | COG1219  COG0563 |
| General | ATP-dependent protease with chaperone activity | ClpP | DR1972 | Dgeo_2151 | TTC0250 | | COG0740 |
| General | ATP-dependent Lon serine protease | Lon | DR1974 DR0349  DR2189 | Dgeo_2153 Dgeo_0427 Dgeo_1487 | TTC0418  TTC0746 | | COG0466  COG2802 |
| General | ATP-dependent serine protease | Sms | DR1105 | Dgeo_1212 | TTC0173 | | COG1066 |
| General | Do serine protease, with regulatory PDZ domain | HtrA | DR0327 DR0745 DR1599 DR1756 DR0984 DR0300 | Dgeo_2185 Dgeo_2025  Dgeo_1495  Dgeo_0676 Dgeo_0552 Dgeo_0103 | TTC0417 TTC0956  TTC1905 | | COG0265 |
| General | Tail-specific periplasmic serine protease | Prc | DR1308 DR1491 DR1551 | Dgeo_0277 Dgeo_1216 Dgeo_1479 | TTC0929 | | COG0793 |
| General | Membrane-associated Zn-dependent protease I | YaeL | DR1507 | Dgeo_1043 | TTC0503 | | COG0750 |
| General | ATP-dependent Zn protease | FtsH | DR0583 DR1020 DRA0290 | Dgeo_2075  Dgeo_1832  Dgeo_2182 | TTC0035 TTC1128 | | COG0465 |
| General | Predicted Zn-dependent proteases (possible chaperones) | HtpX | DR1904  DR0194 | Dgeo_1282 | -  TTC0540 | | COG0501  COG2738 |
| General | Membrane chaperone | SugE | DR1004 DR1005 | Dgeo_1957  Dgeo_1956  Dgeo_2170 | TTC0565 | | COG2076 |
| General | Diadenosine tetraphosphate (Ap4A) hydrolase, HIT family, cell cycle regulation | Hit | DR1621 | Dgeo_1329  Dgeo_2064 | TTC1253 | | COG0537 |
| General | Zn-binding (lipo)protein of the ABC type Zn transport system (surface adhesin A) | YebL | DR2523 | Dgeo_0534 | TTC0227 | | COG0803 |
| General | GTPase, protease modulator | HflX | DR0139 DR0646 | Dgeo_2006  Dgeo_1825 | TTC0198 | | COG2262 |
| Unconfirmed | Fibronectin-binding protein, function unknown | BS_yloA | DR0559 | Dgeo_1423 | TTC0243 | | COG1293 |
| General | General stress protein, related to thioredoxin | BS_ytxJ | DR1832 | Dgeo_1464 |  | | No COG |
| General | Protease I, related to general stress protein 18, ThiJ superfamily protein | ThiJ | DR1199 DR0491 | Dgeo_0863 | TTC1088 TTC1585 | | COG0693 |
| General | Universal stress protein, nucleotide-binding | UspA | DR2363 DR2132 | Dgeo_0155  Dgeo_1279  Dgeo_2742 | TTC0539 TTC1692 TTC1794 TTC1633 | | COG0589 |
| Starvation | Guanosine polyphoshate (ppGpp) pyrophosphohydrolase/synthetase; No RelA counterpart like in Gram+ bacteria | SpoT | DR1838 | Dgeo_1308 | TTC1355 | | COG0317 |
| Unconfirmed | Histone-like DNA-binding protein | HupA | DRA0065 | Dgeo_0175  Dgeo_2501 | TTC0984 | | COG0776 |
| Unconfirmed | Haemoglobin-like flavoprotein | Hmp | DRA0243 | - | - | | No COG |
| Starvation | ppGpp regulated growth inhibitor | MazF | DR0417 DR0662 | Dgeo_1937 | - | | COG2337 |
| Starvation | Regulatory protein, *MazF* antagonist | MazE | DR0416 | Dgeo_1936 | - | | COG2336 |
| Unconfirmed | Phosphatase of ppGpp | Ppx | DRA0185 | - | TTC0636 | | COG0248 |
| Starvation | Starvation inducible DNA-binding protein | Dps | DR2263 DRB0092 | Dgeo_0281 | - | | COG0783 |
| Osmotic | Large conductance mechanosensitive channel | Mscl | DR2422 | Dgeo_0305 | TTC0261 | | COG1970 |
| Osmotic | Membrane protein | Yggb | DR1995 DR0211 | Dgeo_1833 | TTC1353 | | COG0668 |
| Osmotic | Osmosensitive K+ channel Histidine kinase sensor domain | KdpD | DRB0088 | Dgeo_0389  Dgeo_2852 | - | | COG2205 |
| Osmotic | Potassium uptake system, NAD-binding component | TrkA | DR1666 | Dgeo_1584 | TTC0810 | | COG0569 |
| Osmotic | Potassium uptake system component | TrkH/ TrkG | DR1667 DR1668 | Dgeo_1583 | TTC0809 | | COG0168 |
| Osmotic | Proline/ glycine betaine ABC-type transport, permease subunit | ProW | DRA0138 DRA0136 | Dgeo_0171  Dgeo_0173 | - | | COG1174 |
| Osmotic | Proline/ glycine betaine ABC-type transport, ATPase subunit | ProV | DRA0137 | Dgeo_0172 | - | | COG1125 |
| Osmotic | Proline/ glycine betaine ABC-type transport, periplasmic binding subunit | YehZ | DRA0135 | Dgeo_0174 | - | | COG1732 |
| Phage | Phage shock protein A, controls membrane integrity | PspA | DR1473 | Dgeo_0996 | TTC1616 | | COG1842 |
| Alkaline | Alkaline shock protein, function unknown | BS_yloU/ BS_yqhY | DR2068 DR0389 | Dgeo_0697  Dgeo_1721 | TTC0757  TTC1771 | | COG1302 |
| Cold | Cold shock protein, OB fold nucleic acid binding protein | Csp | DR0907 | Dgeo_0638  Dgeo_1006 | TTC1626 TTC1811 | | COG1278 |
| Unconfirmed | Competence damage protein, mitomycin-induced, function unknown | CinA | DR2838 | Dgeo_2136 | TTC1468 | | COG1058/COG1546 |
| Oxidative | Catalase; DRA0259 has C-terminal proteinase I-like domain | KatE | DR1998 DRA0259 | Dgeo_2728 | - | | COG0753 |
| Oxidative | Catalase; Eukaryotic type, presumably acquired from nitrogen–fixing bacteria | KatA S.pombe | DRA0146 | - | - | | COG0753 |
| Oxidative | Peroxidase; Yet present only in plant Polyporaceae sp. | NA | DRA0145 | - | - | | COG2837 |
| Oxidative | Superoxide dismutase Mn or Fe dependent | SodA | DR1279 | Dgeo_0830 | TTC0189 | | COG0605 |
| Oxidative | Superoxide dismutase Cu/Zn dependent | SodC | DR1546 DRA0202  DR0644 | -  Dgeo_0284 | - | | COG2032 |
| Unconfirmed | Ferric uptake regulation protein | Fur | DR0865 | Dgeo_2141  Dgeo_0519  Dgeo_2727 | TTC0925  TTC1639  TTC1730 | | COG0735 |
| Oxidative | Antioxidant type thioredoxin fold protein | Bcp | DR0846 DR1208  DR1209 | Dgeo_2729  Dgeo_0990 | TTC0933  TTP0168 | | COG1225 |
| Oxidative | Protein involved in alkylperoxide and oxidative stress response, osmotically induced protein | OsmC | DR1538 DR1857 | Dgeo_0526  Dgeo_0446 | TTC1261 | | COG1764 |
| Oxidative | Protein involved in alkylperoxide and oxidative stress response, osmotically induced protein | YhfA | DR1177 | Dgeo_1268 | TTC1007 | | COG1765 |
| Oxidative | Peptide methionine sulfoxide reductase PMSR | MsrA | DR1849 | Dgeo_0843 | TTP0095 | | COG0225 |
| Oxidative/ detoxication | Thiol-alkyl hydroperoxide reductases | AhpC | DR2242 DR1209 | Dgeo_0122  Dgeo_0990  Dgeo_2729 | - | | COG0450 |
| Oxidative/ detoxication | Thioredoxin reductase/alkyl hydroperoxide reductase | Ahpf/ TrxB | DR1982 DR2623 DR0412 DRB0033 | Dgeo_2772  Dgeo_1013  Dgeo_1576  Dgeo_2331  Dgeo_0975 | TTC0003 TTC0096 TTC0853 TTC1555  - | | COG0492  COG2072 |
| Oxidative/ detoxication | Glutaredoxin | GrxA | DR2085 DRA0072 | Dgeo_1508  Dgeo_2583 | - | | COG0695 |
| Detoxication | Cytochrome P450 (uses O_2_) | BS_cypA or TerA of Alcalige-nes | DR2473 DR2538 DR1723 DRA0186 DRC0041 DRC0001 | Dgeo_0944  Dgeo_0143 | TTP0059 | | COG2124 |
| Detoxication | Function unknown, involved in tellurium resistance response in *Alcaligenes* | TerB of Alcalige-nes | DR2220 | -- | - | | COG3793 |
| Detoxication | Function unknown, membrane protein | TerC of Alcalige-nes | DR2226; DR1187 DRB0131 | Dgeo_0787  Dgeo_2235 | -  TTC0142 TTC0750 | | COG2899  COG0861 |
| Detoxication | Toxic anion resistance protein , possibly tellurite resistance | BS_yceH | DR1127 | Dgeo_0931 | - | | COG3853 |
| Toxins/ general | Chemical damaging agent resistance; In *Bacillus subtilis*, it is involved in low temperature and salt stress responses, TerZ/TerD-like | BS_Scp2 | DR2225 DR2221 DR2224 DR2223 DRA0057 DR2217 | - | - | | COG2310 |
| Toxins | Arsenate oxidoreductase (arsC-like Rodanese protein) | ArsC | DRA0123DR0136 | Dgeo_0756  Dgeo_2548  Dgeo_2768  Dgeo_2770  Dgeo_0403 | TTC1502 TTC0816 | | COG0394  COG1393 |
| Desiccation | Desiccation protectant, LEA14 family | NA | DR1372 | Dgeo_1551 | - | | No COG |
| Desiccation | Desiccation-related protein from *Craterostigma plantagineum*. To date, found only in plants. | NA | DRB0118 | Dgeo_0097 | TTP0170 | | No COG |
| Desiccation | LEA76 family desiccation resistance protein | NA | DR0105 DR1172 | Dgeo_1473 | -  - | | No COG |
| Drugs | Erythromycin esterase | BS_ybfO | DRA0345 DR2257 | - | - | | COG2312 |
| Drugs | BacA bacitracin resistance protein, undecaprenol kinase | BacA | DR0454 | Dgeo_1051 | TTC1814 | | COG1968 |
| Drugs | Streptomycin resistance protein, streptomycin phosphotransferase | StrA of Strepto-myces | DR0455 | - | - | | COG3570 |
| Drugs | Antibiotic (aminoglycoside); Kinase family protein | BS_ycbJ | DR0066; DRA0194DR0394; DR0669 | Dgeo_2256  Dgeo_2397  Dgeo_1662  Dgeo_2485 | - | | COG3231  COG3173  COG2334  no COG |
| Drugs | 5-Nitroimidazole antibiotic resistance protein; distantly related to pyridoxamine phosphate oxidase, PDXH | NimABCD of Bacteroides | DR0842 | - | - | | COG3467 |
| Drugs | Function unknown, involved in multidrug resistance | BS_bmrU | DR2234 DR1363 DR1560 | Dgeo_2009  Dgeo_1118  Dgeo_1293  Dgeo_2853 | TTC1957 | | COG1597 |
| Drugs | Thiophen and furan oxidation, predicted GTPase | ThdR | DR1016 | Dgeo_0478 | TTC0562 | | COG0486 |
| Drugs | tunicamycin resistance protein, predicted ATPase | BS_tmrB | DR1419 | - | - | | No COG |
| Drugs | Beta-lactamase | BS_penP | DRA0241 DR0433 (+DR1985) | Dgeo_0316  Dgeo_1291 | TTC0404 | | COG2367 |
| Drugs | Function unknown, lactam utilization protein | YbgL | DRA0284 | Dgeo_1803 | TTP0137 | | COG1540 |
| Drugs | Lactoylgluthation lyase, phosphomicin resistance protein | GloA | DR1695 DR2022 DR2104 DR2208 DR0109 DRA0224 DR1341 DR0670 | Dgeo_1712  Dgeo_1790  Dgeo_0360  Dgeo_1981  Dgeo_2419  Dgeo_0941  Dgeo_2415 | TTC0024 TTC0596_2 TTC1190 TTC1694 | | COG0346 |
| Drugs | Induced by Vancomycin in *Enterococcus faecalis* | BS_yoaR | DR1619 DR0009 DR0025 | Dgeo_1593  Dgeo_2332 | TTC1933 | | COG2720 |
| Drugs | aminoglycosid N3-acetyltransferase. Present in many other bacteria. | BS_yokD | DR2034 DR0599 | Dgeo_1202 | - | | COG2746 |
| Drugs | Function unknown, homologs of microcine C7 resistance protein MccF | BS_yocD | DR2000 | - | - | | COG1619 |
| Drugs | 2-nitropropane dioxygenase | BS_yrpB | DR2545 | Dgeo_1494 | TTC1901 | | COG2070 |
| Drugs | phosphinothricin aminoacetyltransferase | BS_ywnH | DR1182 |  | - | | COG1247 |
| **Genes that are absent in *D. radiodurans*** | | | | | | | |
| Starvation | Carbon starvation-induced protein, membrane | CstA | - | - | - | COG1966 | |
| General | ATPase subunit of clp photolytic system | ClpY (HslU) | - | - | TTC0264 | COG1220 | |
| Oxidative | Glutathione peroxidase | BtuE/ BS_bsaA | - | - | - | COG0386 | |
| Oxidative | Catalase (peroxidase I) | KatG | - | - | - | COG0376 | |
| Oxidative | Mn-containing catalase |  | - | - | TTC1872 | COG3546 | |
| Heat/ general | Heat Shock Protein 90, molecular chaperone | HtpG | - | - | - | COG0326 | |
| Heat/ general | *DnaK* suppressor protein | DksA | - | - | - | COG1734 | |
| Heat/ general | Transcriptional regulator of heat shock genes | HrcA | - | - | TTC1948 | COG1420 | |
| Acid | unknown | YajQ | - | - | - | COG1666 | |
| Osmotic | Trehalose-6-phosphate synthase | OtsA | - | Dgeo_0059 | - | COG0380 | |
| Osmotic | Trehalose-6-phosphatase | OtsB | - | Dgeo_0060 | - | COG1877 | |
