# Supplementary material for: Deinococcus geothermalis: The Pool of Extreme Radiation Resistance Genes Shrinks
Source: PLoS One. 2007 Sep 26;2(9):e955. doi: 10.1371/journal.pone.0000955 (PMC1978522; doi:10.1371/journal.pone.0000955)
Supplement: Table S8 — Genes coding for replication, repair and recombination functions in E. coli, D. radiodurans and T. thermophilus. (0.15 MB DOC) [file pone.0000955.s018.doc]

***Table S8. Genes coding for replication, repair and recombination functions^A^ in E. coli, D. radiodurans and T. thermophilus***

| **Pathway^A,B^** | **Protein Description and Comments^C^** | **EC** | **DR** | **TT** | **DG** | **COG**  **number** |
| --- | --- | --- | --- | --- | --- | --- |
| mMM  (tentative) | Adenine-specific DNA  methylase | YhdJ | DRC0020 | TTC0041 TTC1880 | - | COG0863 |
| DR | O-6-methylguanine DNA methyltransferase | Ogt, YbaZ | -  DR0428 | TT1199  - | -  Dgeo_2101 | COG0350  COG3695 |
| DR | 8-oxo-dGTPase. D.r. encodes additional 17 paralogs; only some predicted to function in repair | MutT | DR0261 | TTC0091 | Dgeo_1930 | COG0494 |
| DR, BER | 3-methyladenine DNA glycosylase II; DR2584 is of eukaryotic type | AlkA | DR2584  DR2074 | TTC1654 | Dgeo_0107  Dgeo_1660 | COG0122  COG2094 |
| BER, MMY | 8-oxoguanine DNA glycosylase & AP-lyase, A-G mismatch DNA glycosylase | MutY | DR2285 | TTC1535 | Dgeo_0019 | COG1194 |
| BER | Endonuclease III & thymine glycol DNA glycosylase; | Nth | DR2438 DR0289 DR0928 | TTC1892 | Dgeo_2290  Dgeo_0248  Dgeo_0785 | COG0177 |
| BER | Formamidopyrimidine & 8-oxoguanine DNA glycosylase | MutM/Fpg | DR0493 | TTC1454 | Dgeo_0442 | COG0266 |
| BER | Endonuclease V | Nfi (YjaF) | DR2162 | TTC0982 | - | COG1515 |
| BER | DNA polymerase I | PolA_1_2 | DR1707 | TTC0690 | Dgeo_1666 | COG0258/  COG0749 |
| BER | Uracil DNA glycosylase; DR0689 is a likely horizontal transfer from a eukaryote or a eukaryotic virus | Ung | DR0689  DR1663 | -  - | Dgeo_2059  Dgeo_0448 | COG0692  No COG |
| BER | G/T mismatch-specific thymine DNA glycosylase, distantly related to DR1751; Present as a domain of many multidomain proteins in many eukaryotes | Mug (ygjF) | DR0715 | - | Dgeo_1718  Dgeo_2568 | COG3663 |
| BER | Uracil DNA glycosylase | - | DR1751 | TTC0366 TTC0784 | Dgeo_1556 | COG1573 |
| BER | Exodeoxyribonuclease III | XthA | DR0354 | - | Dgeo_0461  Dgeo_2484 | COG0708 |
| NER, BER | Predicted ATP-dependent protease | Sms (RadA) | DR1105 | TTC0173 | Dgeo_1212 | COG1066 |
| NER | Transcription repair coupling factor; helicase | Mfd | DR1532 | TTC0533 | Dgeo_0545 | COG1197 |
| NER | ATPase, DNA binding | UvrA | DR1771 DRA0188 | TTC1075 | Dgeo_0694 | COG0178 |
| NER | Helicase | UvrB | DR2275 | TTC1531 | Dgeo_1890 | COG0556 |
| NER | Nuclease | UvrC | DR1354 | TTC1182 | Dgeo_1124 | COG0322 |
| NER, mMM, SOS | helicase II; initiates unwinding from a nick; DR1572 has a frameshift | UvrD,  BS_yvgS | DR1775  DR1572 | TTP0191 TTC0638 TTC1062  - | Dgeo_0868  - | COG0210  COG3973 |
| mMM, VSP | predicted ATPase | MutL | DR1696 | TTC0959 | Dgeo_1538 | COG0323 |
| mMM, VSP | ATPase; DR1039 has a frameshift | MutS | DR1976  DR1039 | TTC1282 | Dgeo_0899  Dgeo_1537 | COG1193  COG0249 |
| MM | Exonuclease VII, large subunit | XseA/ nec7 | DR0186 | - | Dgeo_0148 | COG1570 |
| MM | Exonuclease VII, small subunit | XseB | DR2586 | - | Dgeo_0027 | COG1722 |
| RER | Exonuclease subunit, Predicted ATPase | SbcC | DR1922 | TTC0922 | Dgeo_0823 | COG0419 |
| RER | Exonuclease | SbcD | DR1921 | TTC0923 | Dgeo_0824 | COG0420 |
| RER, SOS | Recombinase; ssDNA-dependent ATPase, activator of LexA autoproteolysis | RecA | DR2340 | TTC1466 | Dgeo_2138 | COG0468 |
| RER | Helicase/exonuclease; Contains three additional N-terminal helix-hairpin-helix DNA-binding modules; closely related to RecD from B.subtilis and Chlamydia | RecD | DR1902 | - | Dgeo_0826 | COG0507 |
| RER | Predicted ATPase; required for daughter-strand gap repair | RecF | DR1089 | TTC1721 | Dgeo_1620 | COG1195 |
| RER | Holliday junction-specific DNA helicase; branch migration inducer | RecG | DR1916 | TTC0902 | Dgeo_1139 | COG1200 |
| RER | Single-stranded DNA-specific 5’→3’ exonuclease | RecJ | DR1126 | TTC0803 | Dgeo_1599 | COG0608 |
| RER | Predicted ATPase | RecN | DR1477 | TTC1161 | Dgeo_1194 | COG0497 |
| RER | Required for daughter-strand gap repair | RecO | DR0819 | TTC0258 | Dgeo_0855 | COG1381 |
| RER | Helicase; suppressor of illegitimate recombination | RecQ | DR1289  DR2444 | TTP0128 | Dgeo_1226  Dgeo_0021 | COG0514  No COG |
| RER | Required for daughter-strand gap repair | RecR | DR0198 | TTC1236 | Dgeo_1513 | COG0353 |
| RER | Holliday-junction-binding subunit of the RuvABC resolvasome | RuvA | DR1274 | TTC1696 | Dgeo_0726 | COG0632 |
| RER | Helicase subunit of the RuvABC resolvasome | RuvB | DR0596 | TTC0038 | Dgeo_0404 | COG2255 |
| RER | Endonuclease subunit of the RuvABC resolvasome | RuvC | DR0440 | TTC0725 | Dgeo_0327 | COG0817 |
| MP | Polymerase subunit of the DNA polymerase III holoenzyme | DnaE | DR0507 | TTC1806 | Dgeo_0255 | COG0587 |
| MP | 3’-5’ exonuclease subunit of the DNA polymerase III holoenzyme | DnaQ | DR0856 | TTP0128_1 | Dgeo_0157  Dgeo_1818  Dgeo_2764 FS  Dgeo_2765 FS | COG0847 |
| MP | DNA ligase | LigA  yicF | DR2069 | TTC0732 | Dgeo_0696 | COG0272 |
| MP | Single-strand binding protein; D. radiodurans R1 has three incomplete ORFs corresponding to different fragments of the SSB | Ssb | DR0099 | TTC1741 | Dgeo_0165 | COG0629 |
| SOS | Transcriptional regulator, repressor of the SOS regulon, autoprotease | LexA | DRA0344  DRA0074 | -  - | Dgeo_1366 | COG1974  No COG |
| VSP  (tentative) | Uncharacterized proteins related to vsr | YcjD | DR0221  DR2566 | -  - | -  Dgeo_0873 | COG2852  No COG |
| ? | Uncharacterized family of presumably metal-dependent enzymes | Bs_DinB | 13 homologs (only 3 in COG) | - | 8 homologs | COG2318 |
| DR | xantosine triphosphate pyrophosphatase, prevents 6-N-hydroxylaminopurin mutagenesis | HAM1/YggV | DR0179 | TTC1290 | Dgeo_2209 | COG0127 |
| NER | UV-endonuclease; Activity was characterized in Neurospora | Uve1/BS_YwjD | DR1819 | TTP0052 | Dgeo_1819 | COG4294 |
| NER | DNA or RNA helicase of superfamily II; also predicted nuclease; Contains an additional McrA nuclease domain | YejH/rad25 | DRA0131_1_2 | - | - | COG1061 |
| ? | Topoisomerase IB | - | DR0690 | - | Dgeo_2058 | COG3569 |
| ? | 3'->5' nuclease; Related to baculoviral DNA polymerase exonuclease domain | - | DR1721 | - | - | No COG |
| ? | Ro RNA binding protein; Ribonucleoproteins complexed with several small RNA molecules. Involved in UV-resistance in Deinococcus | - | DR1262 | - | - | No COG |
| ? | Predicted nuclease and Zinc finger domain containing protein. An ortholog is present in Pseudomonas aeruginosa | - | DR1757 | - | - | No COG |
| ? | Mrr-like nuclease;  Restriction endonuclease | -  Mrr | DR1877 DR0508 DR0587 | -  - | -  -  - | COG1787 COG1715 |
| ? | Single strand DNA-binding protein; protects 3’ ends from nuclease degradation | DdrA | DR0423 | TTC1923 | Dgeo_0977 | COG4712 |

^A^Based largely on Makarova et al [S14] with modifications.

^B^Abbreviations of DNA repair pathways: DR- direct damage reversal; BER – base excision repair; NER – nucleotide excision repair; mMM – methylation-dependent mismatch repair; MMY – MutY-dependent mismatch repair; VSP – very short patch mismatch repair; RER – recombinational repair, SOS – SOS repair; MP – multiple pathways; putative, unconfirmed repair pathways are designated by a question mark.

^C^The gene names are from *E. coli*, whenever an *E. coli* ortholog exists, or from *B. subtilis* (with the prefix BS_).

**Supporting Reference**

[S14] Makarova KS, Aravind L, Wolf YI, Tatusov RL, Minton KW, et al. (2001) Genome of the extremely radiation-resistant bacterium *Deinococcus radiodurans* viewed from the perspective of comparative genomics. Microbiol Mol Biol Rev 65: 44-79.
