# Supplementary material for: Deinococcus geothermalis: The Pool of Extreme Radiation Resistance Genes Shrinks
Source: PLoS One. 2007 Sep 26;2(9):e955. doi: 10.1371/journal.pone.0000955 (PMC1978522; doi:10.1371/journal.pone.0000955)
Supplement: Table S9 — Manganese- and iron-related homeostasis genes. (0.08 MB DOC) [file pone.0000955.s019.doc]

**Table S9.** Manganese- and iron-related homeostasis genes

| **Mn-transport and regulation related systems** | | | | | | |
| --- | --- | --- | --- | --- | --- | --- |
| **COG number** | **COG description** | ***D. radiodurans***  **^A^D_10_, ^B^15 kGy** | ***D. geothermalis***  **^A^D_10_, ^B^15 kGy** | ***S. oneidensis***  **^A^D_10_, ^C^0.07 kGy** | ***E. coli***  **^A^D_10_, ^C^0.7 kGy** | **Comments** |
| COG1914 | NRAMP family transporter | DR1709 | Dgeo_0709 | - | mntA | Mn-homeostasis: transport. |
| COG1121 | ABC-type Mn/Zn transport systems, ATPase component | DR2284 | Dgeo_0533 | - | znuC | Mn/Zn-homeostasis: transport. |
| COG1108 | ABC-type Mn/Zn transport systems, permease component | DR2283 | Dgeo_0532 | SO0566 | znuB | Mn/Zn-homeostasis: transport. |
| COG0803 | ABC-type Mn/Zn transport systems, perplasmic component | DR2523 | Dgeo_0534 | SO0565 | - | Mn/Zn-homeostasis: transport. |
| COG1321 | Mn-dependent transcriptional regulator TroR | DR2539 | Dgeo_2268 | - | ybiQ | Mn-homeostasis: regulation. |
| **Fe-transport and regulation related systems** | | | | | | |
| COG3486 | Lysine/ornithine N-monooxygenase | - | - | SO3030 | - | Fe-homeostasis: siderophore biosynthesis. Alcaligin biosynthesis. |
| No COG | Siderophore biosynthesis protein | - | - | SO3031 | - | Fe-homeostasis: siderophore biosynthesis. Alcaligin biosynthesis. |
| COG4264 | Siderophore synthetase component | - | - | SO3032 | - | Fe-homeostasis: siderophore biosynthesis. Alcaligin biosynthesis. |
| COG1528 | Ferritin-like protein, Ftn | - | - | SO0139 | ftn, ftnB | Fe-homeostasis: iron storage. |
| COG1629 | Outer membrane receptor proteins, mostly Fe transport | - | - | SO0630, SO0737, SO2162(domain), SO1156, SO1482, SO1580, SO2427, SO2523, SO2715, SO3514, SO4077 | b1995_1, b1998, fhuA, yncD | Fe-homeostasis: transport. |
| COG4771 | Outer membrane receptor for ferrienterochelin and colicins | - | - | SO4516, SO4523, SO2162(domain), SO2907, SO0719, SO0798, SO2004, SO3669 | cirA, fepA | Fe-homeostasis: transport. |
| COG0810 | Periplasmic protein TonB, links inner and outer membranes | - | - | SO1828, SO3670, SO4043, SO4564 | tonB | Fe-homeostasis: transport. TonB-ExbBD complex provides energy for transfer of ferrisiderophores to the periplasm via the outer membrane. |
| COG4772 | Outer membrane receptor for Fe(III)-dicitrate | - | - | SO1102 | fecA | Fe-homeostasis: transport. |
| COG4773 | Outer membrane receptor for ferric coprogen and ferric-rhodotorulic acid | - | - | SO3033 | fhuE | Fe-homeostasis: transport. |
| COG0783 | DNA-binding ferritin-like protein (oxidative damage protectant), Dps | DR2263, DRB0092 | Dgeo_0281 | SO1158 | dps | Fe-homeostasis: iron storage. Protects DNA from oxidative damage and induced by OxyR. |
| COG1120 | ABC-type cobalamin/Fe(III)-siderophores transport systems, ATPase components | DR2590, DRB0121 | Dgeo_0023,  Dgeo_2759 | SO1033 | fecE, fepC, fhuC | Fe-homeostasis: transport. |
| COG4559 | ABC-type hemin transport system, ATPase component | DRB0016 | Dgeo_0126 | SO3675 | - | Fe-homeostasis: transport. |
| COG4594 | ABC-type hemin transport system, ATPase component | - | Dgeo_2757 | - | fecB |  |
| COG0614 | ABC-type Fe(III)-hydroxamate transport system, periplasmic component | DR0462, DR1373, DR2588, DRB0007, DRB0125 | Dgeo_0025,  Dgeo_2754,  Dgeo_2753,  Dgeo_0657,  Dgeo_1370,  Dgeo_2363 | SO3709 | fhuD, yadT | Fe-homeostasis: transport. |
| COG0609 | ABC-type Fe(III)-siderophore transport system, permease component | DR2589, DRB0015, DRB0122, DRB0123 | Dgeo_2758,  Dgeo_2756,  Dgeo_0024,  Dgeo_0125 | SO1034, SO3674 | fecC, fecD, fepD, fhuB | Fe-homeostasis: transport. |
| COG2375 | Siderophore-interacting protein | DRB0017, DRB0124 | Dgeo_0127 | - | yqjH | Fe-homeostasis: transport. |
| COG1918 | Fe(II) transport system protein A | DR1220 | Dgeo_0895 | SO1783 | feoA | Fe-homeostasis: Fe(II) transport system (active under anaerobic conditions). |
| COG0370 | Fe(II) transport system protein B | DR1219 | Dgeo_0894 | SO1784 | feoB | Fe-homeostasis: Fe(II) transport system (active under anaerobic conditions). |
| COG0735 | Fe(II)/Zn(II) uptake regulation proteins | DR0865 | Dgeo_0519, Dgeo_2141, Dgeo_2727 | SO1937 | fur, zur | Fe-homeostasis: regulation. DR0865 is similar to zur of *B. subtilus*. |

**^A^**D_10_, 10% survival value; **^B^**Figure 1, main text; **^C^**Reference [S15].

**Supporting Reference:** [S15] Daly MJ, Gaidamakova EK, Matrosova VY, Vasilenko A, Zhai M, et al. (2004) Accumulation of Mn(II) in *Deinococcus radiodurans* facilitates gamma-radiation resistance. Science 306: 1025-1028.
