# Supplementary material for: Deinococcus geothermalis: The Pool of Extreme Radiation Resistance Genes Shrinks
Source: PLoS One. 2007 Sep 26;2(9):e955. doi: 10.1371/journal.pone.0000955 (PMC1978522; doi:10.1371/journal.pone.0000955)
Supplement: Table S10 — Parsimony pattern rules for reconstruction of evolutionary events in the Deinococcus/Thermus lineage. (0.11 MB DOC) [file pone.0000955.s020.doc]

**Author Summary**

For fifty years, the bacterium *Deinococcus radiodurans* has been the subject of extensive investigations aimed at solving the mystery of how this organism survives immense doses of gamma rays. So far, whole genome comparisons between *D. radiodurans* and radiation sensitive bacteria have failed to produce a clear picture of the evolution of this resistance phenotype. Most surprisingly, over the last decade, many of the unique *D. radiodurans* genes that were strongly implicated in resistance by their high levels of induction following irradiation have turned out to be unrelated to its survival. This paradox is not yet resolved and has given rise to numerous distinct models of DNA repair in these bacteria. We have acquired the whole-genome sequence of a second extremely radiation resistant bacterium, *Deinococcus geothermalis*, and report a comparison with *D. radiodurans*. Based on those comparisons, we substantially revised down the number of genetic determinants potentially involved in recovery from radiation and desiccation, identified a putative regulator and a palindromic binding site for genes which comprise a distinct *Deinococcus* radiation response regulon, and considered the impact of those findings on the prevailing models of extreme radiation resistance.
